# QIIME2 pipeline for ITS2-based nemabiome sequencing in veterinary species and the importance of analysis parameters

**DOI:** 10.1186/s13071-025-07184-1

**Published:** 2025-12-17

**Authors:** Jeba R. J. Jesudoss Chelladurai, Theresa A. Quintana, Aloysius Abraham

**Affiliations:** 1 Department of Pathobiology, College of Veterinary Medicine, Auburn University, Auburn, AL USA; 2 Scott-Ritchey Research Center, College of Veterinary Medicine, Auburn University, Auburn, AL USA

**Keywords:** Nematoda, Animals, High-throughput nucleotide sequencing, Software, Ribosomal DNA, Computational biology, Workflow

## Abstract

**Background:**

Deep amplicon sequencing of nematode internal transcribed spacer 2 (ITS2), also referred to as the “nemabiome,” has been increasingly used in veterinary hosts to study gastrointestinal nematodes. While post-sequencing bioinformatic pipelines such as DADA2 and mothur have been optimized, most researchers typically use the DADA2 pipeline in R. For optimal performance, DADA2 needs parameter tuning, which is hard for novices.

**Methods:**

In this study, we present an implementation of the DADA2 pipeline within QIIME2 for nemabiome analysis and compare its performance against the commonly used R-based DADA2 pipeline. To evaluate performance against samples with known composition, we generated simulated nemabiome datasets representing canine, ruminant, and equine nematode communities. We also tested the pipelines using publicly available datasets from ten veterinary host species. For both pipelines, we evaluated differences in amplified sequence variant (ASV) generation, taxonomic classification, and diversity metrics. We also tested different Idtaxa parameter settings within the R DADA2 pipeline (classification threshold and bootstrap iterations) to understand its effects on nemabiome outcomes.

**Results:**

While both pipelines showed minor discrepancies in relative abundance estimates, with minimal parameter optimization, QIIME2 outputs were closer to ground truth in simulated datasets. QIIME2 using the scikit Bayes classifier produced fewer unclassified taxa and more consistent species-level identifications compared with R DADA2’s Idtaxa, particularly in complex communities. Community-level differences in beta diversity were primarily driven by differences in taxonomic assignment. Parameter testing revealed that lower classification thresholds in R DADA2 reduced the number of unclassified taxa but increased the risk of misclassification, highlighting the need for careful parameter selection and reporting.

**Conclusions:**

With minimal parameter tuning, QIIME2 outperformed the R pipeline in taxonomic resolution, and improved reproducibility by provenance tracking. Our findings emphasize how bioinformatics pipeline choices impact nemabiome outputs including the number of species detected, ranks of abundant taxa, and alpha and beta diversities. We provide a reproducible and user-friendly QIIME2 workflow suitable for researchers seeking standardized analyses of ITS2 nemabiome data.

**Graphical Abstract:**

**Figure:**
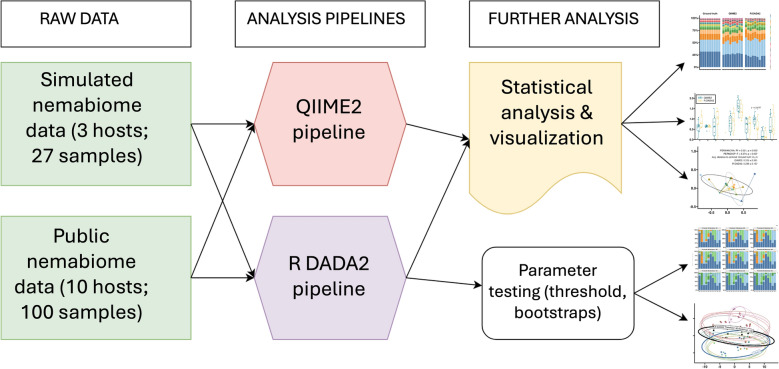

**Supplementary Information:**

The online version contains supplementary material available at 10.1186/s13071-025-07184-1.

## Background

Next generation sequencing technologies in various formats have revolutionized our ability to assess communities of microbes and parasites that adversely affect the health of animals and humans. One of the most frequently used applications of short-read sequencing technology provided by the Illumina platform is targeted deep sequencing of PCR amplicons of specific genomic regions. Deep amplicon sequencing of bacterial and archaeal 16S rDNA is commonly used in microbiology to examine the “microbiome.” For parasites, deep amplicon sequencing of the 18S rRNA gene was the first developed method [1], followed by the mitochondrial cytochrome c oxidase 1 (*cox1*) gene [2]. The internal transcribed spacer 2 (ITS2) region was first used to study nematodes in red kangaroos [3] and cattle [4]. In terrestrial animal species, the ITS2 of Clade V nematodes has increasingly been used to examine the gastrointestinal “nemabiome” of several domestic and wild animal hosts.

ITS2-based nemabiome studies are often used to investigate the composition and relative abundance of nematode communities [4] and to improve species level identification of anthelmintic resistant nematodes [5]. Typically, a nemabiome experiment in terrestrial animal hosts begins at sample collection, followed by parasitological preprocessing such as parasite quantification and larval culture, isolation, DNA extraction, PCR amplification, sequencing on an Illumina instrument, and bioinformatic analysis.

The third larval stage (L3) is the parasite stage generally used in nemabiome studies, although eggs [6, 7] and first larval stage (L1) larvae [7] have been shown to produce similar results. Variations in experimental procedures used by different authors have been reviewed [8]. Frequently used procedures for ITS2 are publicly available at nemabiome.ca [9].

The typical post-sequencing bioinformatic workflow to obtain compositional nemabiome results from Illumina data includes (*i*) demultiplexing raw sequences (FASTQ format) obtained from the sequencer, often performed by the sequencing facility; (*ii*) adapter and primer removal; (*iii*) trimming, error learning, and error-correction (also known as denoising); (iv) dereplicating, that is, collapsing denoised sequences into unique sequences; and finally (v) assigning taxonomies to the sequences on the basis of similarity to sequences in a reference database. Depending on the software used, unique dereplicated sequences are known as amplified sequence variants (ASVs) (in DADA2) or operational taxonomic units (OTUs) (in mothur). The chain of interconnected computational steps from raw data to the generation of tables of unique sequences (ASVs/OTUs), taxa and counts is referred to as a “pipeline.” Further statistical tests and visualizations may later be carried out downstream to answer experimental questions under study.

Pipelines for microbiome analyses are abundant [10], but DADA2 implemented in R is the most frequently used in ITS2 nemabiome analyses [8]. DADA2, first implemented in 2016, is an excellent denoising and dereplication software [11], and is applied after adapter trimming preprocessing of raw data. Alternatives that have been tested for ITS2 nemabiomes include mothur [4, 12] and SCATA [13], and were found to detect relative abundance in a similar manner. However, there is a strong preference for the DADA2 workflow in R among nemabiome researchers. In addition, DADA2 run in R and mothur on the command line are publicly available at nemabiome.ca [9].

A challenge faced by novices using DADA2 in R is the need for previous computational knowledge [13], especially for the optimization of parameters for their datasets [14]. We observed that while DADA2 is also extensively used by microbiome researchers, it was commonly used within the QIIME2 pipeline [15]. QIIME2 provides several advantages, enabling end-to-end analysis that is replicable, repeatable, and transparent. In addition, it automatically tracks metadata and provides data provenance. To our knowledge, there is currently no implementation of ITS2 nemabiome analysis using the QIIME2 platform. Older versions of QIIME1 have been used to analyze the 18S nemabiome in rats [1] and QIIME2 for 18S from marine nematodes [16].

In this study, we implemented a QIIME2-based workflow for ITS2-based nemabiome requiring minimal parameter optimization from the user, and compared it to R DADA2 with the parameters recommended on nemabiome.ca. We tested both pipelines on synthetic simulated nemabiome data and publicly available nemabiome data from published studies derived from ten animal hosts. We analyzed species abundance, diversity, and compositional changes between the two pipelines. In addition, we determined the effect of threshold and bootstrap parameters in the taxonomic classification step of the R DADA2 pipeline.

## Methods

### Simulated data

Simulated mock communities of ITS2 metagenomes were created using ART-illumina v2016.06.05 [17] using selected sequences from the Nematode ITS2 database v1.0.0 [18] as reference. To create reference multi-FASTA files for ART-Illumina, unique taxa for each host group (canines, ruminants, and equines) in the Nematode ITS2 database v1.0.0 were identified and filtered from the Nematode ITS2 database using custom R scripts. The sequences represented 4 nematode species specific to canines, 16 to ruminants, and 22 to equines. Owing to short sequence lengths, *Strongylus* spp. were excluded by ART-Illumina. For each host group, we generated nine simulated mock samples as 250 base pair (bp) paired end amplicon sequences in FASTQ format, with an average DNA fragment size of 300 ± 2 standard deviations with “amplicon” setting in ART-illumina. Parameters used in simulation including varying depth of sequencing coverage (500×, 1000×, or 1500×) and seeds (random or a specific number) are shown in Supplementary Table S1. Additional indel parameters included a first read insertion rate of 0.00009, a second read insertion rate of 0.00015, a first read deletion rate of 0.00011, and a second read deletion rate of 0.00023 (default values). Read metrics of simulated data is shown in Table 1. Since ART-Illumina included the identity of each simulated read in the FASTQ header, the species composition and relative abundance of each simulated set was known and hereafter referred to as the “ground truth” (Supplementary Table S2). Generated FASTQ files and FASTA files used as input have been deposited to Zenodo 10.5281/zenodo.17019940.

**Table 1:** Simulated data metrics and parameters used in processing. Each sample had FASTQ files of different file sizes owing to differences in the simulated depth of sequencing despite the same relative abundance

Host	Size of raw data	Total number of paired reads (in nine samples)	Qiime 2 parameters*	Time for QIIME2 analysis	DADA2 parameters (nemabiome.ca default)^	Time for DADA2 analysis
Simulated canine	81 Mb	270,000	Trim 20, 20 Trunc 220, 220	3 min 39 s	maxEE (fwd:2, rev:5), truncQ = 2; maxMismatch = 1; threshold = 60, bootstraps = 100	12 min 33 s
Simulated ruminant	726 Mb	1,768,000	Trim 20, 20 Trunc 220, 220	6 min 32 s	maxEE (fwd:2, rev:5), truncQ = 2; maxMismatch = 1; threshold = 60, bootstraps = 100	57 min 03 s
Simulated equine	243 Mb	585,000	Trim 20, 20 Trunc 220, 220	3 min 40 s	maxEE (fwd:2, rev:5), truncQ = 2; maxMismatch = 1; threshold = 60, bootstraps = 100	29 min 49 s

*Trim parameters were supplied to code lines p-trim-left-f, p-trim-left-r, p-trunc-len-f, and p-trunc-len-r, respectively, in the qiime dada2 function

^ nemabiome.ca (accessed 7 May 2025) [9]. Parameters for filterAndTrim (maximum error rate and quality truncation), mergePairs (maxMismatch), and IdTaxa (threshold and bootstraps) are provided. Other parameters included nbases = 1× 10^8^ in the learnError function and method = “consensus” in removeBimeraDenovo

### Data from published studies available on NCBI SRA

From previous nemabiome studies, publicly available data was sourced from NCBI’s Sequence Read Archive. Ten samples from each study originating from ten animal hosts—bison, camels, cattle, sheep, goats, horses, pigs, dogs, moose, and kangaroos (100 samples total) were obtained (Table 2). Samples were chosen at random when > 10 samples were present in the original study. All samples had been sequenced using Illumina technology using the primers NC1 and NC2.

**Table 2:** Data metrics and parameters used for data from NCBI GenBank

Host	NCBI SRA dataset accession	NCBI BioProject accession	Size of raw data	Number of paired reads (in selected samples)	Qiime2 DADA2 parameters*	Time for QIIME2 analysis	DADA2 parameters (nemabiome.ca default)^	Time for DADA2 analysis	References
Bison	SRP513219	PRJNA1122240	235 Mb	834,279	Trim 30, 30 Trunc 230, 220	11 min 24 s	maxEE (fwd:2, rev:5), truncQ = 2; maxMismatch = 1; threshold = 60, bootstraps = 100	13 min 04 s	[58]
Camel	SRP584358	PRJNA1261439	249 Mb	836,991	Trim 30, 30 Trunc 250, 210	8 min 42 s	maxEE (fwd:2, rev:5), truncQ = 2; maxMismatch = 1; threshold = 60, bootstraps = 100	16 min 36 s	[59]
Cattle	SRP423439	PRJNA936713	27 Mb	162,405	Trim 30, 30 Trunc 230, 220	4 min 8 s	maxEE (fwd:2, rev:5), truncQ = 2; maxMismatch = 1; threshold = 60, bootstraps = 100	4 min 44 s	[60]
Dog	SRP527832	PRJNA1149426	112 Mb	500,913	Trim 30, 30 Trunc 230, 220	3 min 46 s	maxEE (fwd:2, rev:5), truncQ = 2; maxMismatch = 1; threshold = 60, bootstraps = 100	11 min 27 s	[61]
Goat	SRP423439	PRJNA936713	28 Mb	148,724	Trim 0, 0 Trunc 230, 220	3 min 29 s	maxEE (fwd:2, rev:5), truncQ = 2; maxMismatch = 1; threshold = 60, bootstraps = 100	4 min 57 s	[60]
Horse	SRP548036	PRJNA1190982	152 Mb	349,817	Trim 30, 30 Trunc 230, 220	5 min 42 s	maxEE (fwd:2, rev:5), truncQ = 2; maxMismatch = 1; threshold = 60, bootstraps = 100	6 min 42 s	[50]
Kangaroo	SRP053339	PRJNA274870	1.7 GB	5,128,218	Trim 30, 30 Trunc 200, 200	60 min 55 s	maxEE (fwd:2, rev:5), truncQ = 2; maxMismatch = 1; threshold = 60, bootstraps = 100	72 min 54 s	[3]
Moose	SRP385147	PRJNA856286	323 Mb	1,126,781	Trim 30, 30 Trunc 230, 220	12 min 31 s	maxEE (fwd:2, rev:5), truncQ = 2; maxMismatch = 1; threshold = 60, bootstraps = 100	12 min 57 s	[51]
Pigs	SRP507293	PRJNA1111032	112 Mb	390,622	Trim 30, 30 Trunc 230, 220	4 min 44 s	maxEE (fwd:2, rev:5), truncQ = 2; maxMismatch = 1; threshold = 60, bootstraps = 100	7 min 56 s	[48]
Sheep	SRP287443	PRJNA669542	89 Mb	343,542	Trim 0, 0 Trunc 230, 215	5 min 32 s	maxEE (fwd:2, rev:5), truncQ = 2; maxMismatch = 1; threshold = 60, bootstraps = 100	7 min 19 s	[62]

*Trim parameters were supplied to code lines p-trim-left-f, p-trim-left-r, p-trunc-len-f, and p-trunc-len-r, respectively, in the qiime dada2 function. This step follows adapter removal and trimming using cutadapt

^ nemabiome.ca (accessed 7 May 2025) [9]. Parameters for filterAndTrim (maximum error rate and quality truncation), mergePairs (maxMismatch), and IdTaxa (threshold and bootstraps) are provided. Other parameters included nbases = 1 × 10^8^ in the learnError function and method = “consensus” in removeBimeraDenovo

### QIIME2 pipeline with DADA2

The QIIME2 pipeline [15, 19] was implemented on the high-performance computing (HPC) system at the Alabama Supercomputer Center. All QIIME2 analyses were performed in v2024.10. Synthetic data obtained from ART-Illumina were uploaded to the server with OnDemand [20]. Data from published studies were downloaded using the SRA-Toolkit v3.0.0 [21]. A novel QIIME2 ITS2 database was created using the taxonomic and fasta files from Nematode_ITS2_1.0.0 [18] using the *feature-classifier* [22] with the *naive-bayes* algorithm. FASTQ files were imported using *qiime tools* and *qiime demux* as paired-end data (phred + 33 encoding). Adapters and primers were trimmed using *qiime cutadapt* [23]. Phred quality scores and quality distribution of the reads after adapter trimming were visualized using Qiime2view to estimate the quality trimming parameters for the next step. Read quality trimming parameters were estimated as the sequence base number at which the average phred score was approximately 20 or higher depending on the dataset (trimming parameters are shown in Table 2). Sequences were denoised and dereplicated using *qiime dada2* [11] with trimming parameters previously estimated. QIIME2 parameters used for synthetic data are listed in Table 1 and parameters used for NCBI SRA data are listed in Table 2. Abundance estimates were derived using *qiime feature-table*. Taxonomic classification was performed using the *qiime feature-classifier* which uses the scikit-learn multinomial naïve Bayes machine-learning classifier [22, 24] and results tabulated with *qiime metadata*. A maximum likelihood phylogenetic tree was inferred with *qiime phylogeny*. Abundance, taxonomy, and phylogenetic tree files were downloaded from the server and further analyses were performed on a personal computer. The pipeline was run with script files using a portable batch system (PBS) queue requesting 6 GB of total RAM and three CPU cores (CPU: 2.0 GHz AMD EPYC 7713 Milan) and computational time was calculated from the beginning of *qiime import* to the end of *qiime phylogeny*. Script files (code) used for QIIME2 analysis have been deposited in the Zenodo repository (10.5281/zenodo.17019940). This pipeline is hereafter referred to as “QIIME2.”

### DADA2 pipeline in R

The DADA2 pipeline was applied to the same dataset on the Beocat Research Cluster at Kansas State University. Briefly, FASTQ files were downloaded using command line scripts with SRA-Toolkit v3.0.3 [21]. Adapters were trimmed with *cutadapt* v4.9 [23] run with Python3. Adapter-trimmed files were imported into RStudio on the server running R v4.2.1 and analyzed with the *dada2* pipeline using the analysis parameters recommended on nemabiome.ca [9]. This included using *dada2* functions to assess quality of the adapter trimmed reads (plotQualityProfile), filtering and trimming ends of the reads (filterAndTrim), estimating read errors (learnErrors), denoising reads (dada), merging paired reads (mergePairs), summarizing (makeSequenceTable), and removing chimeras (removeBimeraDenovo). DADA2 parameters used for simulated data are listed in Table 1 and parameters used for NCBI SRA data are listed in Table 2. A taxonomy classifier was trained with *Decipher* v2.26.0 and fasta files from Nematode_ITS2_1.0.0 [18]. Taxonomy was assigned to ASV sequences with the *Idtaxa* function of *Decipher* with a threshold of 60 and 100 bootstraps, as recommended on nemabiome.ca. Abundance, taxonomy, and fasta files representing amplified sequence variants (ASVs) were downloaded from the server and further analyses were performed on a personal computer. A maximum likelihood phylogenetic tree was constructed from the DADA2 sequences representing ASVs with *Decipher* v3.2.0, *phangorn* v2.12.1, *ape* v5.8.1, and *Biostrings* v2.74.1. The pipeline was run with R notebook files requesting 6 GB of total RAM allocated across three CPU cores (CPU: Intel Xeon 5-series/Gold) and computational time was calculated from adapter trimmed file import to the end of *Idtaxa* function; computational time taken by *cutadapt* was added. This pipeline is hereafter referred to as “R DADA2.”

### Effect of parameters in Decipher’s Idtaxa

To understand the effect of threshold and bootstraps used in the *Idtaxa* function on the relative abundance outputs, bootstrap parameters of 100, 200, and 500 were each tested with a threshold of 40–90 in the taxonomy assignments. This resulted in a taxonomic file for each combination of threshold and bootstraps, that could be combined with OTU outputs from R DADA2 as described below.

### Statistical analysis

For statistical analysis and visualization, QIIME2 results were imported to R v4.4.2 using *qiime2R* v0.99.6. DADA2 results were imported to R using *phyloseq* v1.50.0 [25]. Relative abundances for each sample were calculated with *tidyverse* v2.0.0 functions [26]. Barcharts, scatterplots, and boxplots were created using *ggplot2* v3.5.2, *patchwork* v1.3.0, *ggthemes* v5.1.0, and *ggprism* v1.0.5. Alpha diversity measure for each sample was evaluated using the Shannon index with *vegan* v2.7.1 [27] from relative abundances. Dunn’s tests (post hoc pairwise multiple comparisons following Kruskal–Wallis) to compare relative abundances and alpha diversity were performed using *rstatix* v0.7.2 and *ggpubr* v0.6.0. Aitchison distances were calculated using *zcompositions* v1.5.0.4 and *compositions* v2.0–8. Principal coordinate analysis (PCoA) with Aitchison distance was performed to compare beta diversity of species composition between the two pipelines and known ground truth using *compositions* v2.0–8 and *stats* v4.4.2 and plotted with *ggplot2*. For compositional data, Aitchison distance is preferred for beta diversity as it balances dominant and rare taxa [28], unlike Bray–Curtis, which is skewed by abundant species [29]. Permutational multivariate analysis of variance (PERMANOVA) and permutational analysis of multivariate dispersions (PERMDISP) were calculated to assess between-group and within-group variability of the dispersion with *vegan* v2.7.1. The null hypothesis for PERMANOVA was that there was no difference in mean community compositions between analysis pipeline outputs. The null hypothesis for PERMDISP was that average within-group dispersions (variability) was equal across both pipelines (and the ground truth in the simulated dataset).

## Results

### Computed metrics and pipeline comparisons in simulated data

We generated a total of 2,623,000 synthetic sequencing reads to simulate raw nemabiome data from nematode parasite communities (Table 1). These communities were of increasing complexity, including 4 species infectious to canines, 16 to ruminants, and 22 to equines. Each raw data file had different simulated depths of sequencing, leading to different file sizes, despite the same relative abundance for each host. The processing time for each analysis is reported. With the same computational power, the QIIME2 pipeline processed data faster than the R DADA2 pipeline.

**Fig. 1:**
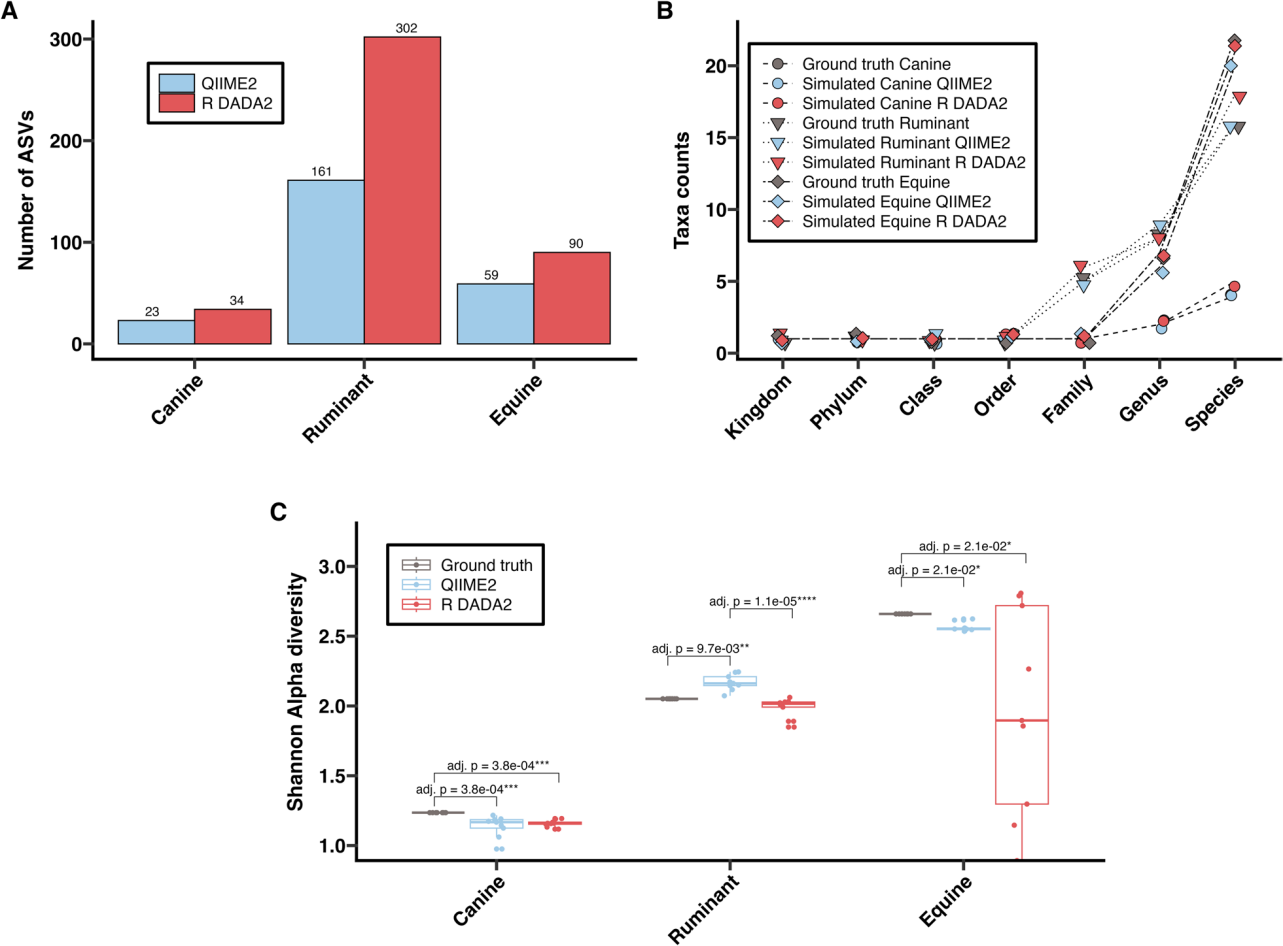
Comparisons of nemabiome metrics computed for three simulated datasets (nine samples each) following processing using the QIIME2 and R DADA2 pipelines. **A** Number of amplified sequence variants (ASVs) generated. **B** Taxa counts at different taxonomic levels. **C** Shannon alpha diversity index calculated with relative abundances; significance based on Dunn’s post-hoc tests (FDR-corrected *p*-values)

ASV counts (Fig. 1A) were consistently higher in R DADA2 output across all simulated datasets. Taxa classification sensitivities across taxonomic levels, following analysis with QIIME2 and R DADA2 compared with the ground truth, are presented in Fig. 1B and Supplementary Table S3. For all three simulated datasets, both pipelines showed agreement with the ground truth at higher taxonomic levels (kingdom, phylum, class, and order). But classification discrepancies were observed at lower levels, driven mainly by “unclassified” taxa present in R DADA2 outputs (detailed below). Shannon alpha diversity index was significantly lower in both pipelines compared with the ground truth for the simulated canine (*P* ≤ 0.001) and simulated equine (*P* ≤ 0.05) datasets, with no significant differences between pipelines. However, in the simulated ruminant dataset, Shannon diversity was significantly higher in QIIME2 compared with both the ground truth and R DADA2 (Fig. 1C).

### Relative abundance comparisons in simulated data

**Fig. 2:**
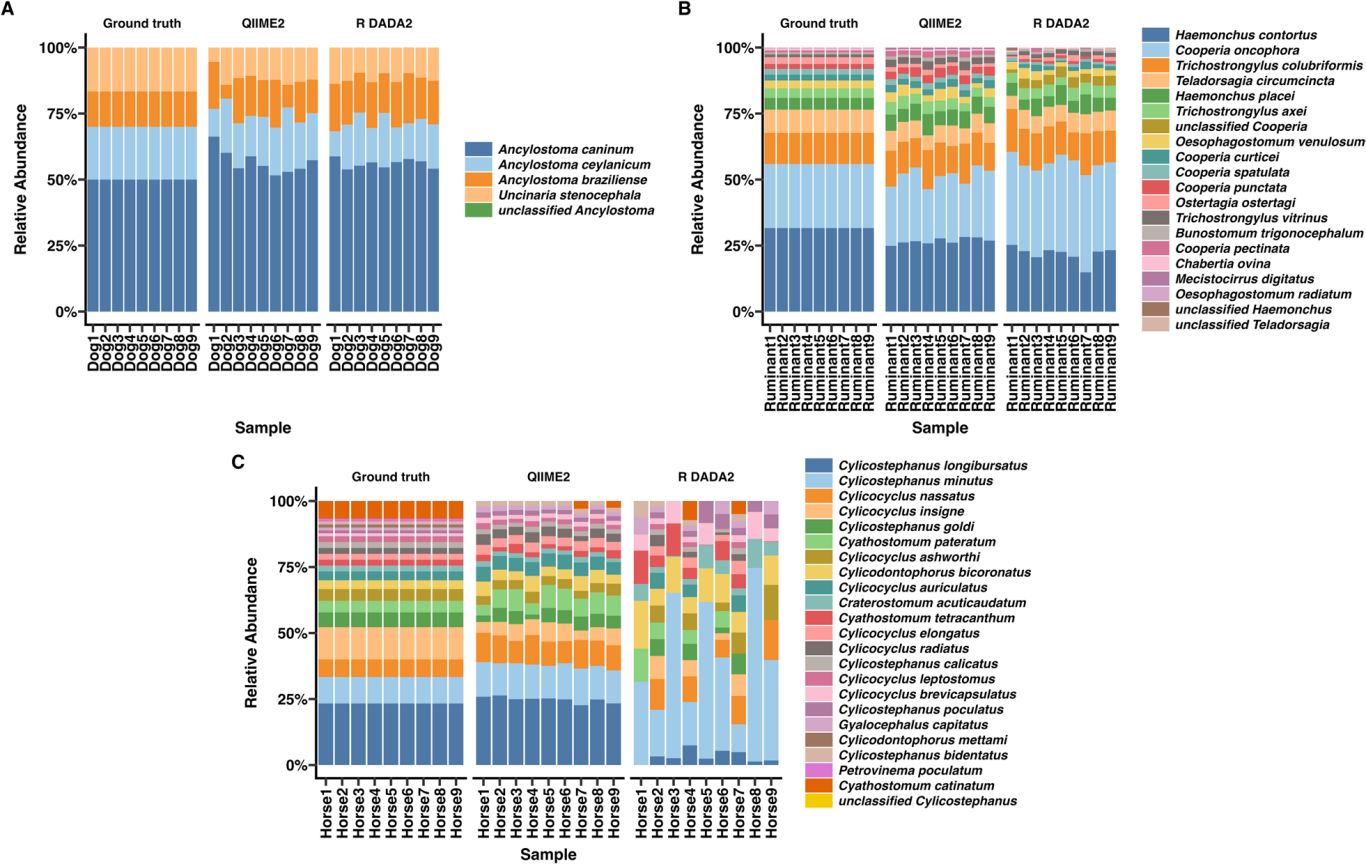
Nemabiome profiles for simulated nemabiome datasets. Relative species abundance in each simulated sample from (**A**) canines, (**B**) ruminants, and (**C**) equines, comparing ground truth with results from QIIME2 and R DADA2 pipelines is shown. Each bar represents a sample and colors represent the nematode species present

Relative abundance estimates varied by nematode species across the simulated datasets compared with ground truth values (Fig. 2). Relative abundances generated by each pipeline for each sample is shown in Supplementary Tables S4–6 and pair-wise species-level comparisons are shown in Supplementary Figs. S1–S3.

In the simulated canine nemabiome, QIIME2 estimates closely matched the ground truth for *Ancylostoma ceylanicum* and *A. braziliense* (Fig. 2A; Supplementary Fig. S1). R DADA2 estimates deviated from the ground truth for all four species. Only R DADA2 output contained unclassified *Ancylostoma*. Pairwise comparisons (Kruskal–Wallis with Dunn post hoc test) revealed that QIIME2 significantly overestimated *A. caninum* (adj. *P* ≤ 0.001) and underestimated *U. stenocephala* (adj. *P* ≤ 0.001) when compared with the ground truth. R DADA2 significantly overestimated *A. caninum* (adj. *P* ≤ 0.001) and *A. braziliense* (adj. *P* ≤ 0.01) and underestimated *A. ceylanicum* (adj. *P* ≤ 0.05) and *U. stenocephala* (adj. *P* ≤ 0.001) when compared with the ground truth.

In the simulated ruminant nemabiome, QIIME2 estimates were similar to the ground truth for 6 of 16 species (*Cooperia oncophora*, *Trichostrongylus colubriformis*, *T. axei*, *Oesophagostomum venulosum*, *C. curticei*, and *C. spatulata*); R DADA2 matched for five species (*O. venulosum*, *C. curticei*, *T. vitrinus*, *Bunostomum trigonocephalum*, and *C. pectinata*) (Fig. 2B; Supplementary Fig. S2). Only R DADA2 output contained unclassified *Strongylida*, *Cooperia*, *Haemonchus*, and *Teladorsagia*. QIIME2 identified *Mecistocirrus* and *M. digitatus*, which were absent from the ground truth. *O. radiatum*, present in the ground truth, was not detected by either pipeline. Pairwise comparisons (Kruskal–Wallis with Dunn post hoc test) revealed that compared with the ground truth, QIIME2 significantly overestimated *H. placei*, *C. punctata*, *T. vitrinus*, *B. trigonocephalum*, and *C. pectinata* (adj. *P* ≤ 0.05–0.0001), and underestimated *H. contortus*, *T. circumcincta*, *Ostertagia ostertagi*, and *C. ovina* (adj. *P* ≤ 0.05–0.001). R DADA2 significantly overestimated *C. oncophora* , *T. colubriformis*, *H. placei*, and *T. axei* (adj. *P* ≤ 0.05–0.001) and underestimated *H. contortus*, *T. circumcincta*, *C. spatulata*, *C. punctata*, *O. ostertagi*, and *C. ovina* (adj. *P* ≤ 0.05–0.0001).

In the simulated equine nemabiome, QIIME2 estimates were similar to the ground truth for 5 of the 22 species (*Cylicostephanus longibursatus*, *Cyathostomum pateratum*, *C. tetracanthum*, *Cylicocyclus ashworthi*, and *C. radiatus*). R DADA2 estimates were similar for nine species (*C. nassatus*, *C. ashworthi*, *C. auriculatus*, *C. elongatus*, *C. radiatus*, *C. pateratum*, *C. tetracanthum*, *C. bidentatus*, and *Craterostomum acuticaudatum*) (Fig. 2C; Supplementary Fig. S3). Only R DADA2 outputs contained unclassified *Cylicostephanus*. Neither pipelines could detect *Cylicodontophorus mettami* and *Petrovinema poculatum*, which were present in the ground truth. Pairwise comparisons (Kruskal–Wallis with Dunn post hoc test) revealed that compared with the ground truth, QIIME2 significant overestimated relative abundances of *C. minutus*, *Cylicocyclus nassatus*, *C. bicoronatus*, *C. auriculatus*, *C. elongatus*, *C. brevicapsulatus*, *C. poculatus*, *Gyalocephalus capitatus*, and *C. bidentatus* (adj. *P* ≤ 0.05–0.01), and underestimated *C. insigne*, *C. goldi*, *C. calicatus*, *C. acuticaudatum*, *C. leptostomus*, and *Cyathostomum catinatum* (adj. *P* ≤ 0.05–0.001). In comparison with the ground truth, R DADA2 overestimated *C. minutus*, *C. bicoronatus*, *C. brevicapsulatus*, *C. poculatus*, and *G. capitatus* (adj. *P* ≤ 0.05–0.0001) and underestimated *C. longibursatus*, *C. insigne*, *C. goldi*, *C. calicatus*, *C. leptostomus*, and *C. catinatum* (adj. *P* ≤ 0.05–0.0001).

### Beta diversity ordination analysis in simulated data

**Fig. 3:**
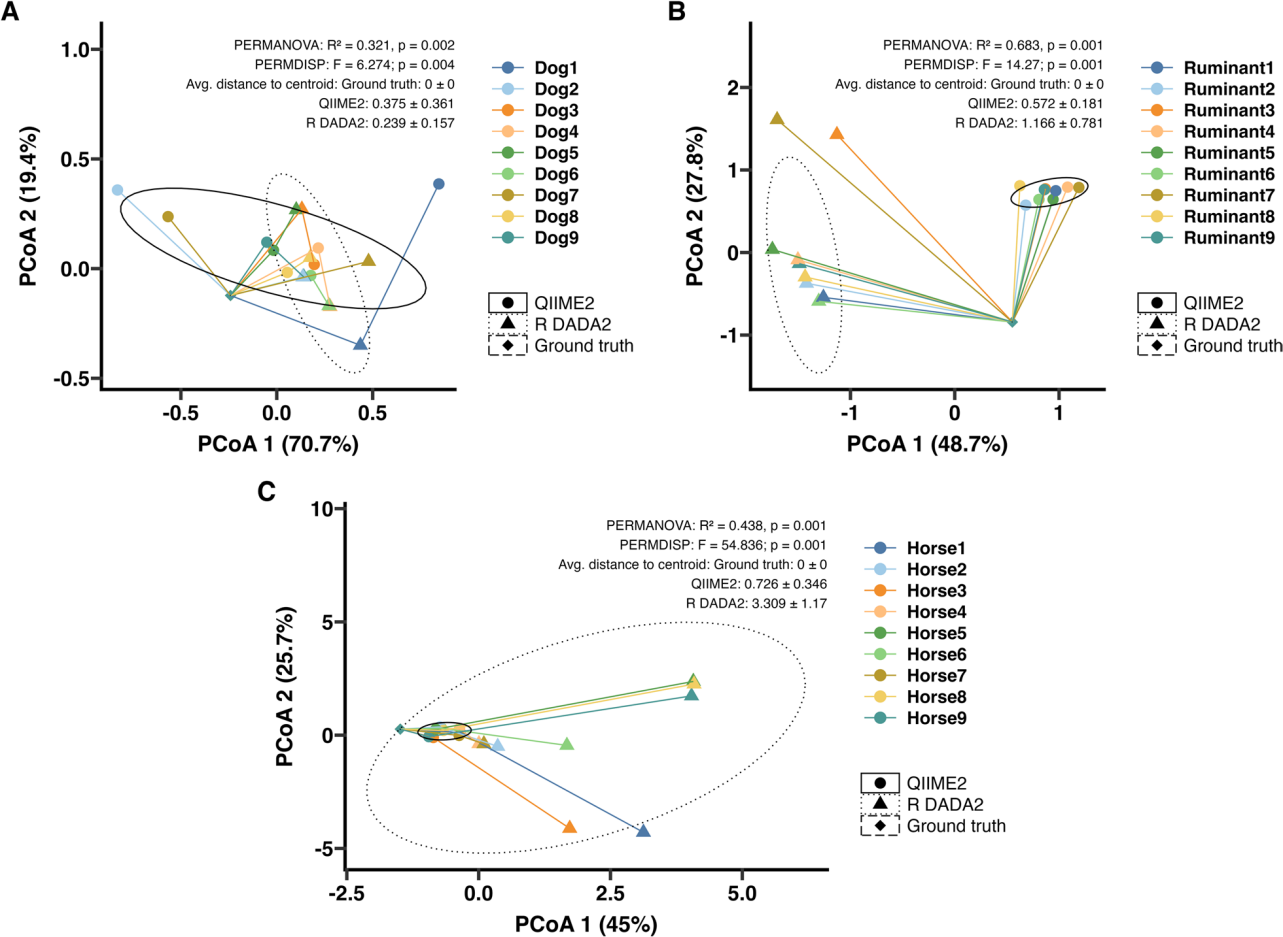
Beta diversity analysis for the simulated nemabiome datasets. Principal coordinates analysis (PCoA) plots with Aitchison distances in simulated datasets from (**A**) canines, (**B**) ruminants, and (**C**) equines, comparing ground truth with results from QIIME2 and R DADA2 pipelines is shown. Statistical values from PERMANOVA, PERMDISP, and average distance to the centroid (mean ± SD) are shown

In all three simulated nemabiome datasets, PCoA with Aitchison distances (Fig. 3) revealed significant differences in community structure between ground truth, QIIME2, and R DADA2 outputs (PERMANOVA *P* ≤ 0.005). However, these differences were driven by dispersion differences within each pipeline output (PERMDISP *P* ≤ 0.01), indicating that PERMANOVA values may not represent true compositional shifts. Interestingly, QIIME2 outputs were most dispersed for the simulated canine nemabiome (Fig. 3A), but R DADA2 outputs were most dispersed for the simulated ruminant and equine nemabiomes (Fig. 3B, C). QIIME2 outputs were more tightly clustered for the simulated ruminant and equine nemabiomes.

### Comparison of QIIME2 and R DADA2 for publicly available SRA data

Ten samples from each from ten veterinary host nemabiomes (*n* = 100 total) (Table 2) were analyzed using both the QIIME2 and R DADA2 pipelines. Bioproject and SRA accession numbers of the datasets are shown in Table 2. Accessions of the individual samples are shown in Figs. 5 and 6. Time taken for the analysis of each dataset by the R DADA2 pipeline was longer than by the QIIME2 pipeline. Larger datasets such as the kangaroo nemabiome required longer computational time in both pipelines.

**Fig. 4:**
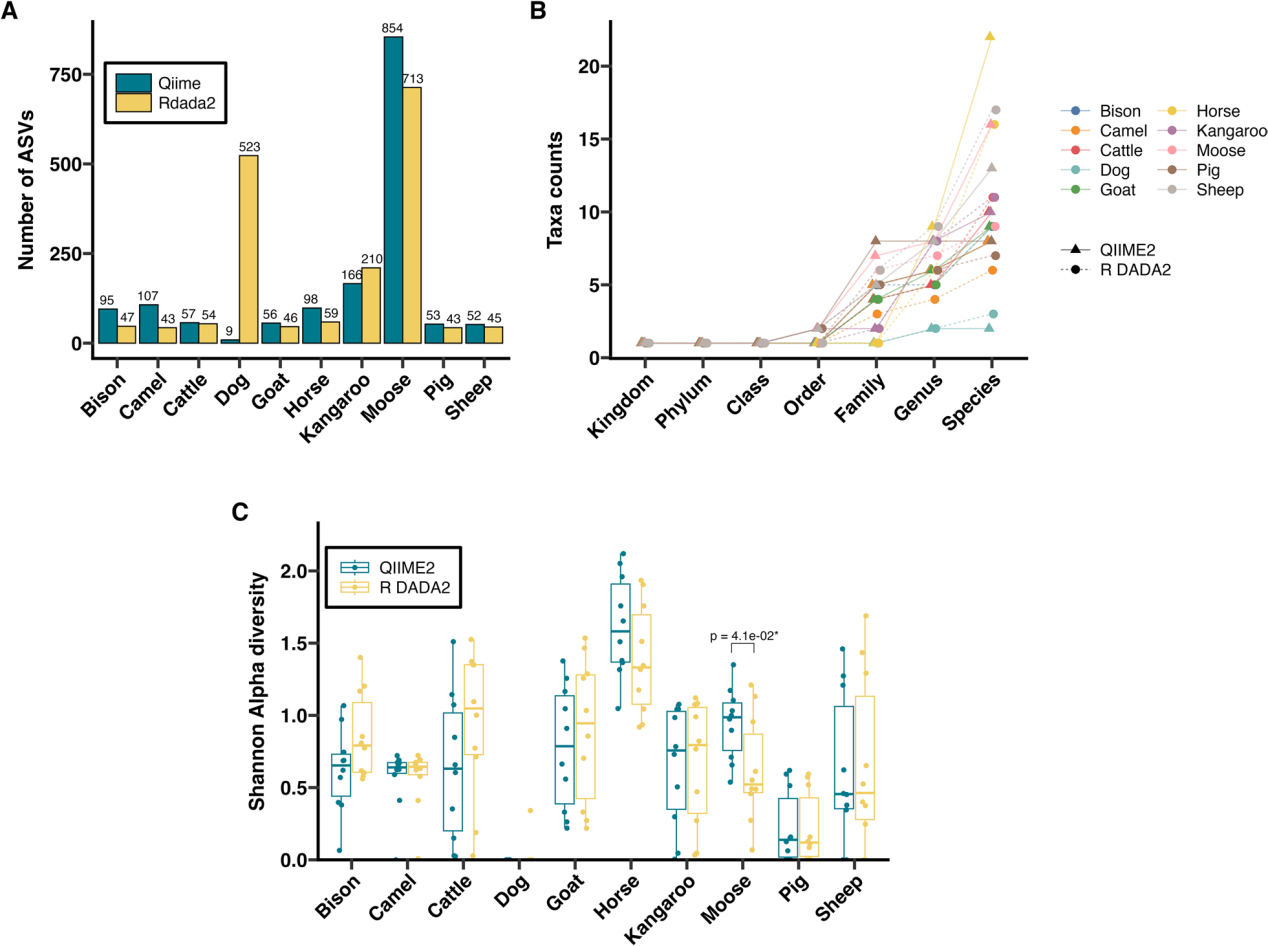
Comparisons of nemabiome metrics computed for NCBI SRA datasets following processing using the QIIME2 and R DADA2 pipelines. **A** Number of amplified sequence variants (ASVs) generated. **B** Taxa counts at different taxonomic levels. **C** Shannon alpha diversity index calculated with relative abundances; significance based on Dunn’s post-hoc tests (FDR-corrected *p*-values)

The number of ASVs generated by each pipeline is shown in Fig. 4A. Interestingly, QIIME2 produced higher ASV counts in eight of the ten host species compared with R DADA2. Taxa counts at various taxonomic levels are presented in Fig. 4B and Supplementary Table S7. While taxa counts were generally consistent at higher taxonomic levels, notable differences were observed at the genus and species levels, particularly in complex communities such as the horse and moose nemabiomes. These differences at the lower taxa levels were driven by “unclassified” taxa, which was exclusive to the R DADA2 pipeline outputs. Shannon alpha diversity for each species was similar for both pipelines in nine hosts (Fig. 4C). In the moose nemabiome, significant differences in alpha diversity were observed between the outputs of the pipelines (Kruskal–Wallis with Dunn post hoc test, *P* ≤ 0.05).

### Relative abundance comparisons in publicly available SRA data

**Fig. 5:**
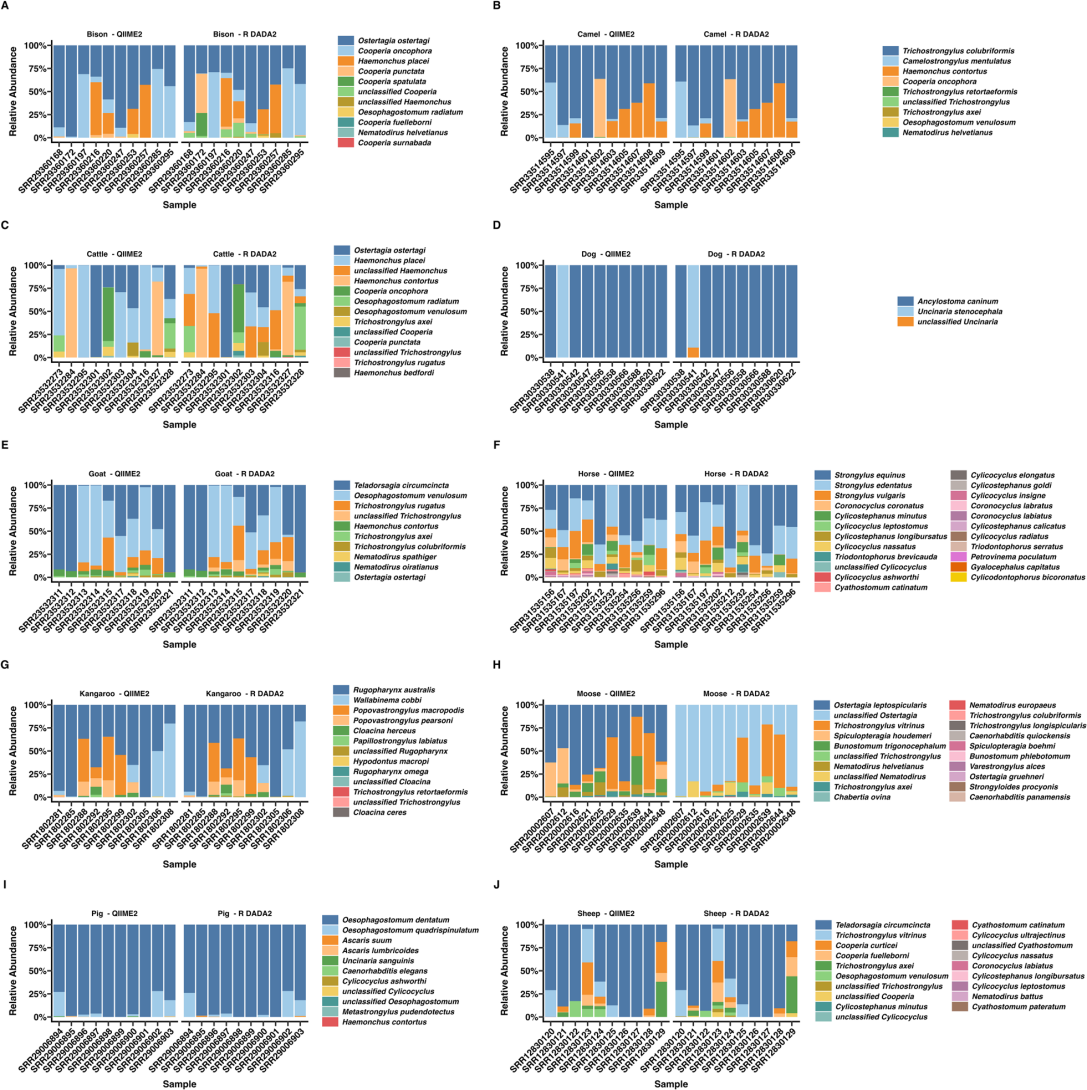
Nemabiome profiles for NCBI SRA datasets analyzed by two pipelines. Relative species abundance in each sample from (**A**) bison, (**B**) camels, (**C**) cattle, (**D**) dogs, (**E**) goats, (**F**) horses, (**G**) kangaroo, (**H**) moose, (**I**) pigs, and (**J**) sheep, comparing results from QIIME2 and R DADA2 pipelines. Each bar represents a sample, indicated on the *x*-axis by SRA accession number, and the colors represent the nematode species present

Relative abundance of nematode species in the outputs of both pipelines across host species is shown in Fig. 5. Interestingly, overall estimates from QIIME2 and R DADA2 were visually similar, except for the moose nemabiome (Fig. 5H), in which clear differences in classification were seen. Pairwise comparisons could be conducted only when nematode species were identified by both pipelines. Comparisons revealed no statistically significant differences for any host species (Kruskal–Wallis with Dunn post hoc test, *P* > 0.05) (Supplementary Figs. S4–S13). Since “unclassified” taxa were not present in QIIME2 outputs, pairwise comparisons could not be conducted for those taxa.

Some notable differences were observed in specific host nemabiomes. In the bison nemabiome (Fig. 5A), QIIME2 detected low levels of *C. fuelleborni* (≤ 0.02% in four samples) and *C. surnabada* (0.01% in three samples), which were absent in R DADA2. Conversely, R DADA2 reported substantially higher abundances of *C. punctata* (42.5%) and *C. spatulata* (24.85%) in sample SRR29360172, both absent in QIIME2. In addition, R DADA2 identified unclassified *Cooperia* (≤ 15.1% in seven samples) and unclassified *Haemonchus* (≤ 4.84% in three samples), which were not detected by QIIME2. In the camel nemabiome (Fig. 5B), QIIME2 uniquely reported low levels of *Nematodirus helvetianus* (≤ 0.04% in three samples), *O. venulosum* (0.22% in one sample), and *T. retortaeformis* (0.61% in one sample). R DADA2 identified unclassified *Trichostrongylus* (≤ 0.64%) in all ten samples, but were not found in QIIME2 outputs. In the cattle nemabiome (Fig. 5C), QIIME2 tended to overestimate *H. placei* in some samples, though mean differences were not statistically significant (*P* > 0.05). QIIME2 also reported *H. bedfordi* (0.05%) and *T. rugatus* (0.32%) in single samples, not detected in R DADA2 outputs. R DADA2 identified unclassified *Haemonchus* (≤ 47.9% in all samples), unclassified *Cooperia* (≤ 4.93%), and unclassified *Trichostrongylus* (0.59% in one sample), none of which were detected by QIIME2. In the dog nemabiome (Fig. 5D), relative abundances were similar across both pipelines. R DADA2 detected 10.74% of unclassified *Uncinaria* in one sample, which was not reported by QIIME2.

In the goat nemabiome (Fig. 5E), QIIME2 uniquely identified *O. ostertagi* (0.03%) in one sample, while R DADA2 reported unclassified *Trichostrongylus* (≤ 12.38%) in seven samples (absent in the QIIME2 output). In the horse nemabiome (Fig. 5F), QIIME2 reported multiple species not detected by R DADA2, including *C. catinatum* (≤ 4% in eight samples), *C. ashworthi* (≤ 3.76% in seven samples), *C. calicatus* (≤ 0.91% in nine samples), *C. radiatus* (≤ 0.89% in three samples), *C. bicoronatus* (≤ 0.09% in two samples), *G. capitatus* (≤ 0.07% in two samples), and *P. poculatum* (0.23% in one sample). R DADA2 uniquely detected unclassified *Cylicocyclus* (≤ 5.57% in four samples). In the kangaroo nemabiome (Fig. 5G), QIIME2 identified *Cloacina ceres* (0.0006%) and *T. retortaeformis* (0.14%) in single samples, while R DADA2 reported unclassified *Rugopharynx* (≤ 1.17% in nine samples), *Cloacina* (≤ 0.41% in two samples), and *Trichostrongylus* (0.11% in one sample), all of which were absent from QIIME2 output. In the moose nemabiome (Fig. 5H), R DADA2 reported significant levels of unclassified *Ostertagia,*, which had been classified as *O. leptospicularis* by QIIME2. QIIME2 also detected *B. trigonocephalum* (≤ 31% in all samples), *B. phlebotomum* (≤ 0.24% in four samples), *Spiculopteragia houdemeri* (≤ 38.23% in nine samples), *S. boehmi* (0.13% in one sample), *N. helvetianus* (≤ 13.9% in nine samples*), Caenorhabditis quiockensis* (< 0.21% in four samples), *C. panamensis* (0.02% in one sample), *O. gruehneri* (≤ 0.03% in two samples), *Strongyloides procyonis* (0.03% in one sample), and *T. longispicularis* (≤ 0.32% in two samples). In the pig nemabiome (Fig. 5I), QIIME2 detected *Ascaris lumbricoides* (≤ 0.91% in five samples), *C. ashworthi* (0.04% in one sample), *H. contortus* (0.03% in one sample), and *U. sanguinis* (≤ 0.78% in three samples), all absent from R DADA2 outputs. R DADA2 identified *A. suum* (≤ 1.28% in three samples), unclassified *Cylicocyclus* (0.05% in one sample), and unclassified *Oesophagostomum* (≤ 0.08% in three samples) not reported by QIIME2. In the sheep nemabiome (Fig. 5J), QIIME2 uniquely detected *C. pateratum* (0.02%) and *C. leptostomus* (0.07%) in single samples. R DADA2 reported *C. catinatum* (0.05% in one sample), *N. battus* (0.01% in one sample), unclassified *Cooperia* (≤ 5.19% in three samples), *Cyathostomum* (0.04% in one sample), *Cylicocyclus* (0.05% in one sample), and *Trichostrongylus* (≤ 3.59% in three samples), all absent from QIIME2 outputs.

### Beta diversity ordination analysis in SRA data

**Fig. 6:**
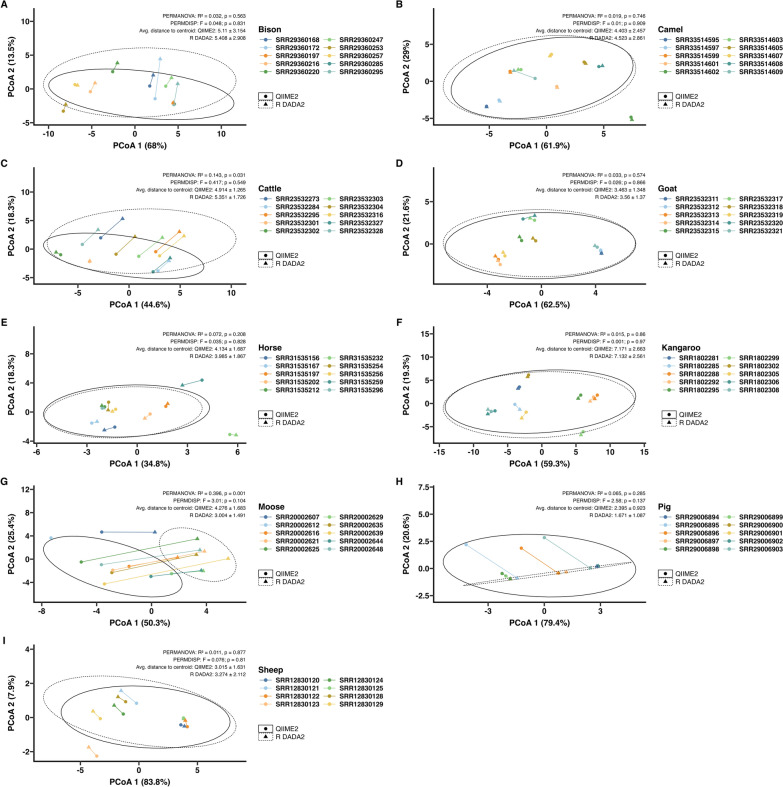
Sample-level beta diversity analysis for NCBI SRA datasets analyzed by two pipelines. Principal coordinates analysis (PCoA) plots with Aitchison distances calculated at the sample level from (**A**) bison, (**B**) camel, (**C**) cattle, (**D**) goat, (**E**) horse, (**F**) kangaroo, (**G**) moose, (**h**) pig, and (**i**) sheep, comparing results from QIIME2 and R DADA2 pipelines is shown. Ellipses represent the 90% confidence intervals. Statistical values from PERMANOVA, PERMDISP, and average distance to the centroid (mean ± SD) are shown

Principal coordinates analysis (PCoA) on the basis of Aitchison distance was performed to assess differences in community composition between the QIIME2 and R DADA2 pipelines (Fig. 6). PCoA was not performed on the dog nemabiome owing to low species richness (*n* = 2). PCoA plots for bison, camel, goat, horse, kangaroo, pig, and sheep (Fig. 6A, B, D–F, H, I) showed no distinct clustering by analysis pipeline. These observations were supported by PERMANOVA (*P* > 0.05) and PERMDISP (*P* > 0.05), indicating no significant differences in community composition or dispersion. In contrast, the cattle nemabiome (Fig. 6C) exhibited partial separation by pipeline. PERMANOVA indicated a statistically significant difference between pipelines (*R*^2^ = 0.143, *P* = 0.023), while PERMDISP showed no significant difference in community dispersion (*F* = 0.417, *P* = 0.558). Similarly, the moose nemabiome (Fig. 6G) demonstrated clear clustering by pipeline (PERMANOVA *R*^2^ = 0.396, *P* = 0.001). PERMDISP results (*F* = 3.01, *P* = 0.1) indicated this difference was not due to within-community dispersion.

### Effect of Idtaxa parameters in R DADA2

**Fig. 7:**
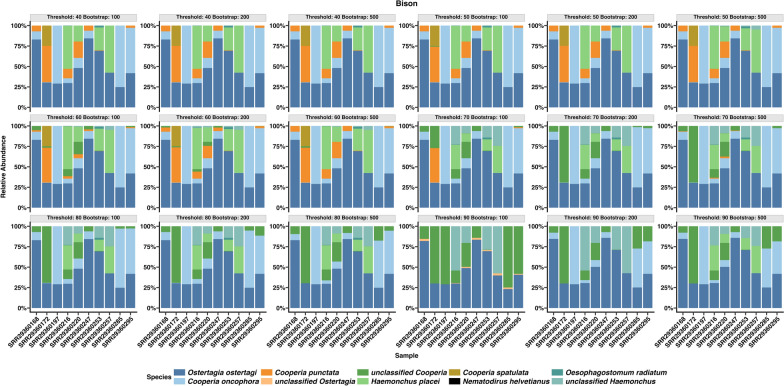
Effect of threshold and bootstrap settings in R DADA2 on relative abundance estimation in bison nemabiome communities

Idtaxa parameters in R DADA2 were tested to assess their impact on the relative abundance of nematode species, including the effect of classification thresholds ranging from 40% to 90% and bootstrap values of 100, 200, and 500. Threshold had a significant effect on taxonomic classification. In the bison nemabiome (Fig. 7), lower thresholds (40–60%) enabled more species-level classifications with fewer unclassified taxa. However, increasing the threshold to 70% led to a rise in unclassified *Cooperia*. Similar trends were observed in other hosts (barplots by samples: Supplementary Figs. S14–S22. Boxplots by species: Supplementary Figs. S23–S32), though the threshold at which unclassified taxa appeared varied. This threshold ranged between 60% for the dog nemabiome (unclassified *Uncinaria* appeared) to ~90% for the horse nemabiome (a marked reduction in the genus *Strongylus* while *Coronocyclus*, *Cylicostephanus*, and *Triodontophorus* spp. became more prominent). In kangaroos, increasing the threshold to 80% resulted in several misclassifications and “NA”s were obtained at a threshold of 90%.

**Fig. 8:**
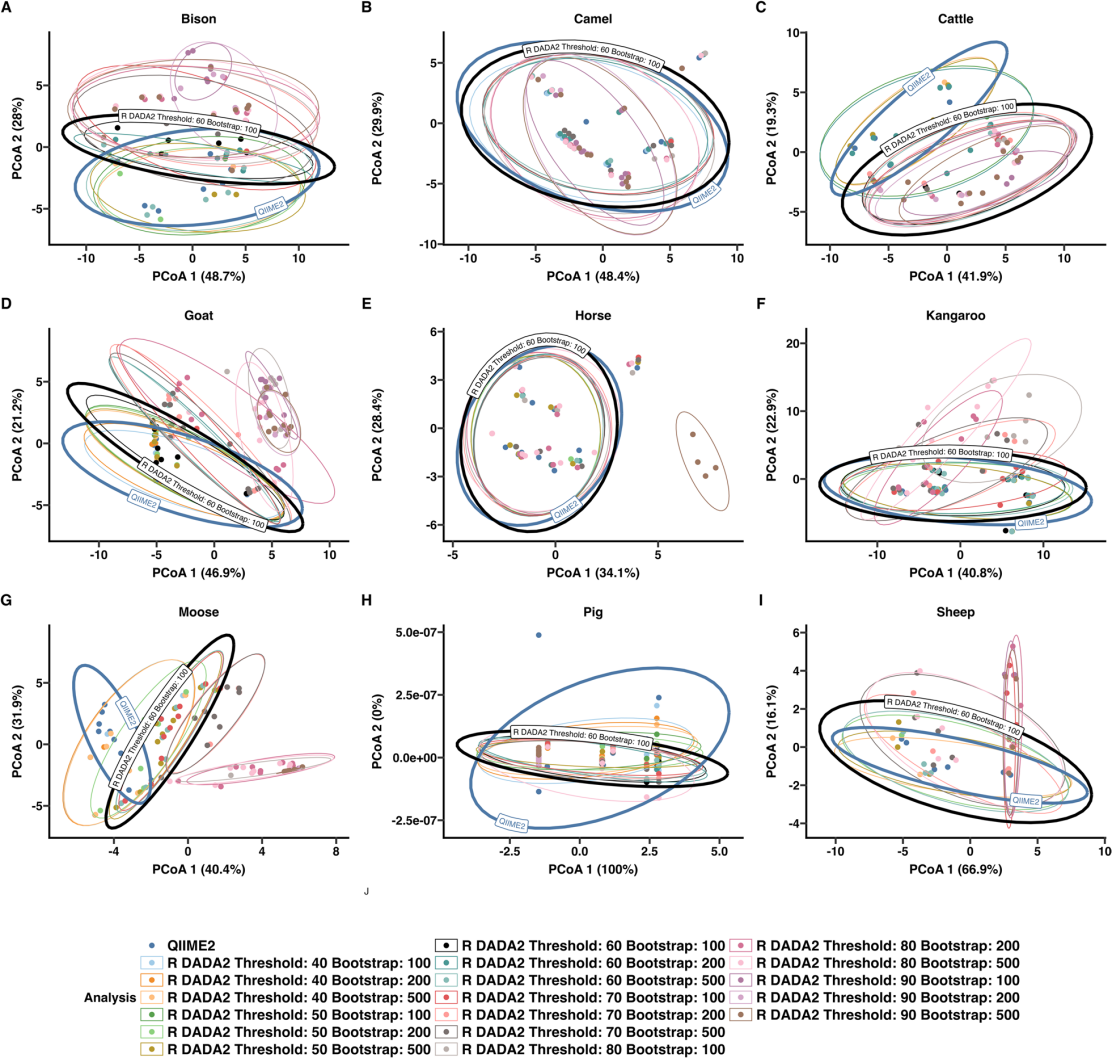
Sample-level beta diversity analysis to assess the effect of threshold and bootstrap settings in R DADA2 compared with QIIME2. Principal coordinates analysis (PCoA) plots with Aitchison distances calculated at the sample level from (**A**) bison, (**B**) camel, (**C**) cattle, (**D**) goat, (**E**) horse, (**F**) kangaroo, (**G**) moose, (**H**) pig, and (**I**) sheep. Ellipses represent the 90% confidence interval with QIIME2 and the standard R DADA2 setting

PCoA plots based on Aitchison distance (Fig. 8) revealed that changes in classification thresholds changed overall community composition. High thresholds in complex nemabiome communities such as in bison, camel, cattle, kangaroos, moose, sheep, and goats caused dramatic shifts in the structure of the community, while there were minor shifts in structure in the horse and pig nemabiome. Bootstrap values had an overall lesser effect on community structure.

## Discussion

DNA metabarcoding by PCR amplification followed by high-throughput sequencing is becoming a widely adopted technique to understand nematode community structure in animal hosts. Post-sequencing analyses need careful implementation to capture the intricacies of the studied population [10, 30]. Two major requirements of the bioinformatics tools used in deep amplicon sequencing processing are (*i*) correctly assigning taxonomic identification to amplicon sequences on the basis of the provided database and (*ii*) providing accurate abundance counts for the assigned taxa. The pipeline used in bioinformatic analysis has a direct impact on the relative abundance and diversity metrics computed [31–34]. For metabarcoding of Clade V nematodes, the ITS2 region is commonly targeted and often processed using DADA2 [11] in R, which often requires parameter optimization. However, this is a steep learning curve for novice users, since ground truth is unknown in animal samples. In this study, we present an implementation of DADA2 using the QIIME2 platform in Linux for ITS2 nemabiome data, using default settings with dataset-specific trimming lengths. We demonstrate its use in both simulated and publicly available (NCBI SRA) datasets from ten veterinary hosts, and compare its performance with a DADA2 implementation in R using parameter settings commonly applied in parasitology. QIIME2 offers a modular, reproducible workflow with built-in data provenance, making it a valuable tool for standardizing nemabiome analyses [19].

Inherent biological variation at the species-level and individual worm-level leads to interspecific and intraspecific variations in sequences in nematodes [35]. This is well characterized for the ITS2 region of Clade V nematodes [36, 37]. In addition, Illumina sequencing introduces non-random errors, toward the ends of reads, driven primarily by sample handling, library preparation method, sequencing library quality, library size selection, choice of primers, sequencing chemistry (including adapter sequences), and instrument settings [38, 39]. These errors must be addressed in the experimental design and/or data analysis [40]. Software such as DADA2, called denoisers, correct sequencing errors to distinguish biological variation and noise [41]. DADA2 uses error models learned from the sequencing run/dataset provided to resolve amplicon sequence variants (ASVs) [11].

In the DADA2 pipeline, several parameters allow large amounts of flexibility in the analysis but may significantly influence ASV output. Important parameters include filtering thresholds (maximum error rate, quality truncation), error learning (number of bases used), read merging (maximum mismatch allowed), chimera removal method, and taxonomic classification settings (Idtaxa threshold, bootstrap). Common defaults in nemabiome analyses for R DADA2 [9] include maxEE (fwd 2, rev 5), truncQ of 2, nbases of 1e8, maxMismatch of 1, and consensus chimera removal. In our implementation, QIIME2’s DADA2 uses similar defaults (maxEE fwd 2 rev 2, truncQ 2) but a lower nbases (1e6) and stricter merging (max_merge_mismatch 0). In addition, we provided QIIME2 with 5′ and 3′ trimming lengths based on quality profiles after using Qiime2View to visualize sequence quality after adapter trimming. For taxonomy, QIIME2 uses a naïve Bayes classifier [22, 24], demonstrating high accuracy under default conditions, while R DADA’s popular implementation uses Idtaxa. Both pipelines output ASV read counts, sequences, and taxonomic classifications. Our QIIME2 pipeline additionally provides a phylogenetic tree. In our hands, the QIIME2 pipeline demonstrated faster processing times than R DADA2. The faster processing time may be a consideration depending on computation power costs.

Several factors complicate the interpretation of nemabiome data. Similar to microbiome data [42], nemabiome outputs are compositional. Abundance values are relative, non-negative, and constrained by sequencing output [42, 43]. In addition, ribosomal gene (rDNA) copy numbers and intergenic spacers such as ITS2, vary across parasitic nematode species. While correction factors have been proposed for certain host species such as cattle, bison [4], and sheep [7], they are not universally available for all hosts. Further, no information is available for parasitic nematodes about potential selection pressures on rRNA genes, as observed in *C. elegans* [44], or intra-individual ITS variation reported in plant-parasitic nematodes [45]. Thus, it is extremely important for researchers to understand that nemabiome analyses do not provide absolute abundances. Relative abundance estimates of nematode species in each sample is the fraction of reads classified as that species out of the total reads for the sample that pass quality control within the pipeline.

Bioinformatic pipelines are often tested with simulated datasets [46]. In this study, we generated simulated nemabiome datasets representing simple (canine) and complex (ruminant, equine) communities. The 27 simulated samples served as the first mock nematode datasets for evaluating QIIME2 and R DADA2 pipelines. In these datasets, R DADA2 consistently generated more ASVs and a higher number of “unclassified” species than QIIME2. A major reason for these differences between QIIME2 and R DADA2 is that the algorithms for classification (scikit Bayes versus Idtaxa) are different. Currently, there are no directly interchangeable implementations of these classifiers between QIIME2 and R to make direct comparisons. At its core, the Idtaxa algorithm utilizes machine learning to reduce over-classification errors, leading to withholding classification from sequences not represented in the reference database training set [47], QIIME2’s scikit naïve Bayes algorithm uses probabilistic models based on *k*-mers, to balance precision and recall, leading to classifying more sequences into taxa [22]. In this study, both pipelines showed over- or underestimation of relative abundance at the species level, but differences from ground truth were more frequent with R DADA2 (Fig. 2; Supplementary Figs. S1–S3). These differences affected overall community structure, with QIIME2 outputs being on average closer to ground truth (Fig. 3). In studies using nemabiome metabarcoding for ecological inferences or surveillance, such discrepancies may alter fundamental biological conclusions drawn in the study, especially when ground truth is unknown. A standardized quantitative threshold of acceptable discrepancy (if detected by utilizing more than one pipeline for the analysis) between methods does not currently exist and will depend on the research questions under study. Therefore, the choice of pipeline may impact research questions, such as number of species present (Fig. 1B), alpha diversity (Fig. 1C), top five species ranked by abundance (Fig. 2C), and beta-diversity (Fig. 3).

In publicly available (NCBI SRA) datasets from ten veterinary hosts, QIIME2 produced more ASVs in eight host species, except for dog and horse nemabiomes (Fig. 4A). Taxa counts were comparable between pipelines (Fig. 4B), though R DADA2 still reported more unclassified taxa. Shannon alpha diversity was similar between pipelines except for the moose nemabiome (Fig. 4C). Shannon alpha diversity can also be calculated directly from ASVs, and such calculations may be appropriate depending on the analysis questions under study. Despite general agreement in species-level relative abundances (Fig. 5), significant differences in beta diversity (PERMANOVA *P* < 0.05) were observed in cattle and moose between pipelines (Fig. 6), largely owing to R DADA2 classifying some abundant reads as unclassified. Additional contributors to community structure differences included low-abundance taxa (< 1%) detected only by QIIME2, often representing aberrant host associations. These included *C. fuelleborni* (of African wild ruminants) in U.S. bison, *T. retortaeformis* (of lagomorphs) in camels and kangaroos, *H. bedfordi* (of African wild ruminants) in Australian cattle, *O. ostertagi* (of bovids) in goats, *S. procyonis* (of racoons) in moose, *C. ashworthi* (of equids), *H. contortus* (of ruminants), and *U. sanguinis* (of sea lions) in pigs, and *C. pateratum* and *C. leptostomus* (of equids) in sheep. In publications, these aberrant taxa can be removed if the reason for their occurrence is known [48]. Another reason is low sequence divergence between closely related species such as *C. curticei* and *C. fuelleborni*, which may result in misclassification, as previously reported [49].

In several datasets, QIIME2 identified species not detected by R DADA2 unless the original study used modified pipelines. For instance, in the horse nemabiome, QIIME2 identified multiple Cyathostominae species that were absent from R DADA2 unless pipeline adjustments were made [50]. The kangaroo dataset, originally processed with UCHIME [3], was reanalyzed here using DADA2 for the first time. Specific genera were not identified in the original study likely owing to a lack of comprehensive reference databases at the time of publication of the kangaroo nemabiome. Similarly, in the moose nemabiome study, a Bayesian classifier in R was used to assess genus-level (not species) classifications [51]. This missed key species such as *B. trigonocephalum* and *B. phlebotomum*. Low levels of *Caenorhabditis* spp. were also detected despite rectal sampling. In pigs, *A. suum* and *A. lumbricoides* were represented in R DADA2 and QIIME2 outputs, respectively. However, since the infective L3 stages are retained within the eggs until infection, the detection of this species represents cross-contamination likely owing to high *Ascaris* egg burdens in the samples, as suggested in the original study [48]. However, *G. urosubulatus*, reported in low abundance [48], was not recovered by either pipeline, though low levels of *C. elegans* was found. In sheep, both pipelines detected strongyle species of equines.

*Nematodirus* spp. were frequently observed in ruminant nemabiomes, but require cautious interpretation, since this genus shows variation in larval hatching. They also have a unique temperature range for hatching with an upper limit of 17 °C [52]. Some *Nematodirus* populations have a requirement for exposure to cold conditions, while others do not [53]. Therefore, in studies involving larval hatching by fecal culture, *Nematodirus* spp. may or may not be properly represented.

The effects of classification thresholds and bootstrap parameters in R DADA2 were evaluated across nine datasets (Fig. 7; Supplementary Figs. S14–S32). In IDTAXA, lower thresholds led to deeper species-level assignments with fewer “unclassified” taxa, while higher thresholds reduced classification depth and increased the number of unclassified ASVs. At a threshold of 100 (data not shown), a large fraction of ASVs were labeled as “NA.” Higher thresholds also caused noticeable shifts in sample clustering (Fig. 8), while increasing bootstrap iterations tightened clustering. Both parameters should be reported in future nemabiome studies to ensure reproducibility.

The QIIME2 pipeline can be adapted to other genetic markers used in nemabiome studies, such as 18S [1], *cox1* [54, 55], 28S, 12S, and 16S [56], as long as a suitable reference database is available. Comprehensive databases have been developed for 18S [57] and nematode ITS2 [18]. While we performed post-pipeline analysis in R, QIIME2 offers several plug-ins that can create and explore data visualizations, diversity calculations, and other statistics. A frequently updated website with tutorials and forums to support QIIME2 is also available.

The findings of this study must be interpreted in light of its limitations. Despite its ease, installing and using QIIME2 requires basic Linux computational knowledge to handle files on the command line. Some of these may be mitigated by the availability of tools such as OnDemand, but these may not be available on all servers. We acknowledge that more than basic Linux knowledge is often required to troubleshoot both QIIME2 and R DADA2 when errors occur. In this study, we generated simulated data using ART-Illumina [17], which simulates sequencing errors (indels and substitutions) on the basis of read quality profiles, but does not simulate PCR artifacts and chimera formations. In this study, we used ten randomly selected samples per host from publicly available SRA datasets, without considering original experimental designs. To make this clear, we used accession numbers from NCBI SRA to indicate individual samples, and do not use original sample names. Calculated diversity metrics apply only to the selected samples and cannot be extrapolated to the original studies for comparison. In addition, we did not use any correction factors, since they are not available for all hosts. Time taken for the analyses should be interpreted with caution. Despite the use of 6 GB cores on both servers, the efficiency of memory usage on each server was subject to factors outside of our control. Finally, the QIIME2 pipeline with minimal parameter optimization was compared with a standard R DADA2 implementation (as described on nemabiome.ca) likely to be used by novice users, not to the modified pipelines used in some original studies [50].

## Conclusions

This study presents a QIIME2-based pipeline for nemabiome analysis using the ITS2 region and compared its performance with a commonly used R-based DADA2 implementation. Using both simulated and publicly available datasets from ten veterinary hosts, we show that while both pipelines produce similar outputs, QIIME2 was consistently faster, yielded fewer unclassified taxa, and provided more accurate estimates of composition at the species level. In addition, parameter settings such as threshold and bootstrap iterations in the R DADA2 pipeline significantly influenced outputs, highlighting the need for careful parameter selection and reporting. Our QIIME2 implementation provides a practical alternative to R DADA2 for analyzing nemabiome sequencing data.

## Supplementary Information

Additional file 1.

## Data Availability

Simulated nemabiome FASTQ files, FASTA files used as input, and QIIME2 script files have been deposited to Zenodo 10.5281/zenodo.17019940.
