# Supplementary material for: QIIME2 pipeline for ITS2-based nemabiome sequencing in veterinary species and the importance of analysis parameters
Source: Parasit Vectors. 2025 Dec 17;19:39. doi: 10.1186/s13071-025-07184-1 (PMC12822254; doi:10.1186/s13071-025-07184-1)
Supplement: Supplementary file 1 — Additional file 1. [file 13071_2025_7184_MOESM1_ESM.docx]

Table of Contents

[Table S1 : Parameters used for simulating data using ART-illumina 3](#_Toc207646138)

[Table S2. Ground truth abundance in the raw Simulated nemabiome data. 4](#_Toc207646139)

[Table S3: Taxonomic classification accuracy of simulated nematode communities following processing with QIIME 2 and DADA2 pipelines compared to the ground truth. 6](#_Toc207646140)

[Table S4. Comparison of relative abundance estimates from QIIME 2 and DADA2 pipelines against ground truth values for the simulated canine nemabiome. 7](#_Toc207646141)

[Table S5. Comparison of relative abundance estimates from QIIME 2 and DADA2 pipelines against ground truth values for the simulated ruminant nemabiome. 9](#_Toc207646142)

[Table S6. Comparison of relative abundance estimates from QIIME 2 and DADA2 pipelines against ground truth values for the simulated equine nemabiome. 14](#_Toc207646143)

[Table S7: Taxonomic classification accuracy of NCBI SRA nemabiome communities following processing with QIIME 2 and DADA2 pipelines. Ground truth is not known. 20](#_Toc207646144)

[Figure S1: Pairwise comparison of the simulated canine nemabiome. 21](#_Toc207646145)

[Figure S2 : Pairwise comparison of the simulated ruminant nemabiome 22](#_Toc207646146)

[Figure S3: Pairwise comparison of the simulated equine nemabiome. 23](#_Toc207646147)

[Figure S4: Pairwise comparison of the NCBI SRA bison dataset. 24](#_Toc207646148)

[Figure S5: Pairwise comparison of the NCBI SRA camel dataset. 25](#_Toc207646149)

[Figure S6: Pairwise comparison of the NCBI SRA cattle dataset. 26](#_Toc207646150)

[Figure S7: Pairwise comparison of the NCBI SRA dog dataset. 27](#_Toc207646151)

[Figure S8: Pairwise comparison of the NCBI SRA goat dataset. 28](#_Toc207646152)

[Figure S9: Pairwise comparison of the NCBI SRA horse dataset. 29](#_Toc207646153)

[Figure S10: Pairwise comparison of the NCBI SRA kangaroo dataset. 30](#_Toc207646154)

[Figure S11: Pairwise comparison of the NCBI SRA moose dataset. 31](#_Toc207646155)

[Figure S12: Pairwise comparison of the NCBI SRA pig dataset. 32](#_Toc207646156)

[Figure S13: Pairwise comparison of the NCBI SRA sheep dataset. 33](#_Toc207646157)

[Figure S14: Barplot showing the effect of threshold and bootstrap settings in R DADA2 on relative abundance estimation in camel nemabiome communities. 34](#_Toc207646158)

[Figure S15: Barplot showing the effect of threshold and bootstrap settings in R DADA2 on relative abundance estimation in cattle nemabiome communities. 35](#_Toc207646159)

[Figure S16: Barplot showing the effect of threshold and bootstrap settings in R DADA2 on relative abundance estimation in dog nemabiome communities. 36](#_Toc207646160)

[Figure S17: Barplot showing the effect of threshold and bootstrap settings in R DADA2 on relative abundance estimation in goat nemabiome communities. 37](#_Toc207646161)

[Figure S18: Barplot showing the effect of threshold and bootstrap settings in R DADA2 on relative abundance estimation in horse nemabiome communities. 38](#_Toc207646162)

[Figure S19: Barplot showing the effect of threshold and bootstrap settings in R DADA2 on relative abundance estimation in kangaroo nemabiome communities. 39](#_Toc207646163)

[Figure S20: Barplot showing the effect of threshold and bootstrap settings in R DADA2 on relative abundance estimation in moose nemabiome communities. 40](#_Toc207646164)

[Figure S21: Barplot showing the effect of threshold and bootstrap settings in R DADA2 on relative abundance estimation in pig nemabiome communities. 41](#_Toc207646165)

[Figure S22: Barplot showing the effect of threshold and bootstrap settings in R DADA2 on relative abundance estimation in sheep nemabiome communities. 42](#_Toc207646166)

[Figure S23: Boxplot showing the effect of threshold and bootstrap settings in R DADA2 on relative abundance estimation in bison nemabiome communities. 43](#_Toc207646167)

[Figure S24: Boxplot showing the effect of threshold and bootstrap settings in R DADA2 on relative abundance estimation in camel nemabiome communities. 44](#_Toc207646168)

[Figure S25: Boxplot showing the effect of threshold and bootstrap settings in R DADA2 on relative abundance estimation in cattle nemabiome communities. 45](#_Toc207646169)

[Figure S26: Boxplot showing the effect of threshold and bootstrap settings in R DADA2 on relative abundance estimation in dog nemabiome communities. 46](#_Toc207646170)

[Figure S27: Boxplot showing the effect of threshold and bootstrap settings in R DADA2 on relative abundance estimation in goat nemabiome communities. 47](#_Toc207646171)

[Figure S28: Boxplot showing the effect of threshold and bootstrap settings in R DADA2 on relative abundance estimation in horse nemabiome communities. 48](#_Toc207646172)

[Figure S29: Boxplot showing the effect of threshold and bootstrap settings in R DADA2 on relative abundance estimation in kangaroo nemabiome communities. 49](#_Toc207646173)

[Figure S30: Boxplot showing the effect of threshold and bootstrap settings in R DADA2 on relative abundance estimation in moose nemabiome communities. 50](#_Toc207646174)

[Figure S31: Boxplot showing the effect of threshold and bootstrap settings in R DADA2 on relative abundance estimation in pig nemabiome communities. 51](#_Toc207646175)

[Figure S32: Boxplot showing the effect of threshold and bootstrap settings in R DADA2 on relative abundance estimation in sheep nemabiome communities. 52](#_Toc207646176)

### Table S1 : Parameters used for simulating data using ART-illumina

| Sample names in the simulated canine dataset | Sample names in the simulated equine dataset | Sample names in the simulated ruminant dataset | Fold of read coverage over reference sequences | Seed for simulation |
| --- | --- | --- | --- | --- |
| Dog 1 | Horse1 | Ruminant1 | 500 | Random |
| Dog 2 | Horse2 | Ruminant2 | 1000 | Random |
| Dog 3 | Horse3 | Ruminant3 | 1500 | Random |
| Dog 4 | Horse4 | Ruminant4 | 500 | 123 |
| Dog 5 | Horse5 | Ruminant5 | 1000 | 123 |
| Dog 6 | Horse6 | Ruminant6 | 1500 | 123 |
| Dog 7 | Horse7 | Ruminant7 | 500 | 876 |
| Dog 8 | Horse8 | Ruminant8 | 1000 | 876 |
| Dog 9 | Horse9 | Ruminant9 | 1500 | 876 |

### Table S2. Ground truth abundance in the raw Simulated nemabiome data.

| Dataset | Species | Relative abundance in % |
| --- | --- | --- |
| Simulated canine nemabiome |  |  |
|  | *Ancylostoma caninum* | 50 |
|  | *Ancylostoma ceylanicum* | 20 |
|  | *Uncinaria stenocephala* | 16.67 |
|  | *Ancylostoma braziliense* | 13.33 |
| Simulated Ruminant nemabiome |  |  |
|  | *Haemonchus contortus* | 31.62 |
|  | *Cooperia oncophora* | 24.26 |
|  | *Trichostrongylus colubriformis* | 11.76 |
|  | *Teladorsagia circumcincta* | 8.82 |
|  | *Haemonchus placei* | 4.41 |
|  | *Trichostrongylus axei* | 3.68 |
|  | *Oesophagostomum venulosum* | 2.94 |
|  | *Ostertagia ostertagi* | 2.57 |
|  | *Cooperia curticei* | 2.21 |
|  | *Cooperia spatulata* | 2.21 |
|  | *Cooperia punctata* | 1.84 |
|  | *Trichostrongylus vitrinus* | 1.10 |
|  | *Bunostomum trigonocephalum* | 0.74 |
|  | *Chabertia ovina* | 0.74 |
|  | *Cooperia pectinata* | 0.74 |
|  | *Oesophagostomum radiatum* | 0.37 |
| Simulated equine nemabiome |  |  |
|  | *Cylicostephanus longibursatus* | 23.33 |
|  | *Cylicocyclus insigne* | 12.22 |
|  | *Cylicostephanus minutus* | 10 |
|  | *Cylicocyclus nassatus* | 6.67 |
|  | *Cyathostomum catinatum* | 6.67 |
|  | *Cylicostephanus goldi* | 5.56 |
|  | *Cylicocyclus ashworthi* | 4.44 |
|  | *Cyathostomum pateratum* | 4.44 |
|  | *Cylicodontophorus bicoronatus* | 3.33 |
|  | *Cylicocyclus auriculatus* | 3.33 |
|  | *Cylicostephanus calicatus* | 2.22 |
|  | *Cylicocyclus radiatus* | 2.22 |
|  | *Cylicocyclus leptostomus* | 2.22 |
|  | *Cylicocyclus elongatus* | 2.22 |
|  | *Cyathostomum tetracanthum* | 2.22 |
|  | *Craterostomum acuticaudatum* | 2.22 |
|  | *Petrovinema poculatum* | 1.11 |
|  | *Gyalocephalus capitatus* | 1.11 |
|  | *Cylicostephanus poculatus* | 1.11 |
|  | *Cylicostephanus bidentatus* | 1.11 |
|  | *Cylicodontophorus mettami* | 1.11 |
|  | *Cylicocyclus brevicapsulatus* | 1.11 |

### Table S3: Taxonomic classification accuracy of simulated nematode communities following processing with QIIME 2 and DADA2 pipelines compared to the ground truth.

| Dataset | Kingdom | Phylum | Class | Order | Family | Genus | Species |
| --- | --- | --- | --- | --- | --- | --- | --- |
| Simulated canine Qiime2 | 1 | 1 | 1 | 1 | 1 | 2 | 4 |
| Simulated canine R Dada2 | 1 | 1 | 1 | 1 | 1 | 2 | 5 |
| Ground truth Dog | 1 | 1 | 1 | 1 | 1 | 2 | 4 |
| Simulated Ruminant Qiime2 | 1 | 1 | 1 | 1 | 5 | 9 | 16 |
| Simulated Ruminant R Dada2 | 1 | 1 | 1 | 1 | 6 | 8 | 18 |
| Ground truth Ruminant | 1 | 1 | 1 | 1 | 5 | 8 | 16 |
| Simulated equine Qiime2 | 1 | 1 | 1 | 1 | 1 | 6 | 20 |
| Simulated equine R Dada2 | 1 | 1 | 1 | 1 | 1 | 7 | 21 |
| Ground truth Horse | 1 | 1 | 1 | 1 | 1 | 7 | 22 |

### Table S4. Comparison of relative abundance estimates from QIIME 2 and DADA2 pipelines against ground truth values for the simulated canine nemabiome.

| Sample | Species | Ground | Qiime2 | dada2 |
| --- | --- | --- | --- | --- |
| Dog1 | *Ancylostoma braziliense* | 0.133333 | 0.177669 | 0.179682 |
| Dog1 | *Ancylostoma caninum* | 0.5 | 0.662592 | 0.587593 |
| Dog1 | *Ancylostoma ceylanicum* | 0.2 | 0.105134 | 0.095072 |
| Dog1 | *Uncinaria stenocephala* | 0.166667 | 0.054605 | 0.137653 |
| Dog1 | *unclassified Ancylostoma* | 0 | 0 | 0 |
| Dog2 | *Ancylostoma braziliense* | 0.133333 | 0.051561 | 0.165367 |
| Dog2 | *Ancylostoma caninum* | 0.5 | 0.601193 | 0.538507 |
| Dog2 | *Ancylostoma ceylanicum* | 0.2 | 0.205542 | 0.169667 |
| Dog2 | *Uncinaria stenocephala* | 0.166667 | 0.141705 | 0.12646 |
| Dog2 | *unclassified Ancylostoma* | 0 | 0 | 0 |
| Dog3 | *Ancylostoma braziliense* | 0.133333 | 0.170246 | 0.150967 |
| Dog3 | *Ancylostoma caninum* | 0.5 | 0.542561 | 0.552382 |
| Dog3 | *Ancylostoma ceylanicum* | 0.2 | 0.171027 | 0.201154 |
| Dog3 | *Uncinaria stenocephala* | 0.166667 | 0.116166 | 0.095497 |
| Dog3 | *unclassified Ancylostoma* | 0 | 0 | 0 |
| Dog4 | *Ancylostoma braziliense* | 0.133333 | 0.151673 | 0.172897 |
| Dog4 | *Ancylostoma caninum* | 0.5 | 0.588104 | 0.56493 |
| Dog4 | *Ancylostoma ceylanicum* | 0.2 | 0.15316 | 0.130999 |
| Dog4 | *Uncinaria stenocephala* | 0.166667 | 0.107063 | 0.13113 |
| Dog4 | *unclassified Ancylostoma* | 0 | 0 | 4.38E-05 |
| Dog5 | *Ancylostoma braziliense* | 0.133333 | 0.138907 | 0.149901 |
| Dog5 | *Ancylostoma caninum* | 0.5 | 0.55135 | 0.545879 |
| Dog5 | *Ancylostoma ceylanicum* | 0.2 | 0.186307 | 0.206952 |
| Dog5 | *Uncinaria stenocephala* | 0.166667 | 0.123436 | 0.097268 |
| Dog5 | *unclassified Ancylostoma* | 0 | 0 | 0 |
| Dog6 | *Ancylostoma braziliense* | 0.133333 | 0.180558 | 0.171561 |
| Dog6 | *Ancylostoma caninum* | 0.5 | 0.516029 | 0.566056 |
| Dog6 | *Ancylostoma ceylanicum* | 0.2 | 0.180972 | 0.131323 |
| Dog6 | *Uncinaria stenocephala* | 0.166667 | 0.122441 | 0.13106 |
| Dog6 | *unclassified Ancylostoma* | 0 | 0 | 0 |
| Dog7 | *Ancylostoma braziliense* | 0.133333 | 0.08528 | 0.189612 |
| Dog7 | *Ancylostoma caninum* | 0.5 | 0.529206 | 0.577767 |
| Dog7 | *Ancylostoma ceylanicum* | 0.2 | 0.244159 | 0.135437 |
| Dog7 | *Uncinaria stenocephala* | 0.166667 | 0.141355 | 0.096796 |
| Dog7 | *unclassified Ancylostoma* | 0 | 0 | 0.000388 |
| Dog8 | *Ancylostoma braziliense* | 0.133333 | 0.153612 | 0.155689 |
| Dog8 | *Ancylostoma caninum* | 0.5 | 0.540851 | 0.568727 |
| Dog8 | *Ancylostoma ceylanicum* | 0.2 | 0.175557 | 0.16129 |
| Dog8 | *Uncinaria stenocephala* | 0.166667 | 0.12998 | 0.114294 |
| Dog8 | *unclassified Ancylostoma* | 0 | 0 | 0 |
| Dog9 | *Ancylostoma braziliense* | 0.133333 | 0.126393 | 0.164843 |
| Dog9 | *Ancylostoma caninum* | 0.5 | 0.573223 | 0.541197 |
| Dog9 | *Ancylostoma ceylanicum* | 0.2 | 0.178854 | 0.167797 |
| Dog9 | *Uncinaria stenocephala* | 0.166667 | 0.121531 | 0.126164 |
| Dog9 | *unclassified Ancylostoma* | 0 | 0 | 0 |

### Table S5. Comparison of relative abundance estimates from QIIME 2 and DADA2 pipelines against ground truth values for the simulated ruminant nemabiome.

| Sample | Species | Ground | Qiime2 | dada2 |
| --- | --- | --- | --- | --- |
| Ruminant1 | *Bunostomum trigonocephalum* | 0.007353 | 0.012156 | 0.00975 |
| Ruminant1 | *Chabertia ovina* | 0.007353 | 0.005594 | 0.006282 |
| Ruminant1 | *Cooperia curticei* | 0.022059 | 0.027969 | 0.002975 |
| Ruminant1 | *Cooperia oncophora* | 0.242647 | 0.224828 | 0.352892 |
| Ruminant1 | *Cooperia pectinata* | 0.007353 | 0.020116 | 0 |
| Ruminant1 | *Cooperia punctata* | 0.018382 | 0.028507 | 0 |
| Ruminant1 | *Cooperia spatulata* | 0.022059 | 0.021837 | 0.011133 |
| Ruminant1 | *Haemonchus contortus* | 0.316176 | 0.248817 | 0.252549 |
| Ruminant1 | *Haemonchus placei* | 0.044118 | 0.061209 | 0.048937 |
| Ruminant1 | *Mecistocirrus digitatus* | 0 | 0.007853 | 0 |
| Ruminant1 | *Oesophagostomum radiatum* | 0.003676 | 0 | 0 |
| Ruminant1 | *Oesophagostomum venulosum* | 0.029412 | 0.03636 | 0.028758 |
| Ruminant1 | *Ostertagia ostertagi* | 0.025735 | 0.01775 | 0.006221 |
| Ruminant1 | *Teladorsagia circumcincta* | 0.088235 | 0.075947 | 0.049888 |
| Ruminant1 | *Trichostrongylus axei* | 0.036765 | 0.047762 | 0.038051 |
| Ruminant1 | *Trichostrongylus colubriformis* | 0.117647 | 0.135112 | 0.162018 |
| Ruminant1 | *Trichostrongylus vitrinus* | 0.011029 | 0.028184 | 0.006566 |
| Ruminant1 | *unclassified Cooperia* | 0 | 0 | 0.012935 |
| Ruminant1 | *unclassified Haemonchus* | 0 | 0 | 0.011046 |
| Ruminant1 | *unclassified Teladorsagia* | 0 | 0 | 0 |
| Ruminant2 | *Bunostomum trigonocephalum* | 0.007353 | 0.009986 | 0.009363 |
| Ruminant2 | *Chabertia ovina* | 0.007353 | 0.004993 | 0.004946 |
| Ruminant2 | *Cooperia curticei* | 0.022059 | 0.021532 | 0.013582 |
| Ruminant2 | *Cooperia oncophora* | 0.242647 | 0.261457 | 0.324294 |
| Ruminant2 | *Cooperia pectinata* | 0.007353 | 0.01587 | 0 |
| Ruminant2 | *Cooperia punctata* | 0.018382 | 0.023894 | 0.009534 |
| Ruminant2 | *Cooperia spatulata* | 0.022059 | 0.019882 | 0.00994 |
| Ruminant2 | *Haemonchus contortus* | 0.316176 | 0.261813 | 0.228567 |
| Ruminant2 | *Haemonchus placei* | 0.044118 | 0.048859 | 0.038423 |
| Ruminant2 | *Mecistocirrus digitatus* | 0 | 0.00584 | 0 |
| Ruminant2 | *Oesophagostomum radiatum* | 0.003676 | 0 | 0 |
| Ruminant2 | *Oesophagostomum venulosum* | 0.029412 | 0.04293 | 0.040699 |
| Ruminant2 | *Ostertagia ostertagi* | 0.025735 | 0.013864 | 0.014829 |
| Ruminant2 | *Teladorsagia circumcincta* | 0.088235 | 0.075205 | 0.072169 |
| Ruminant2 | *Trichostrongylus axei* | 0.036765 | 0.049349 | 0.043941 |
| Ruminant2 | *Trichostrongylus colubriformis* | 0.117647 | 0.119829 | 0.138977 |
| Ruminant2 | *Trichostrongylus vitrinus* | 0.011029 | 0.024697 | 0.01487 |
| Ruminant2 | *unclassified Cooperia* | 0 | 0 | 0.035866 |
| Ruminant2 | *unclassified Haemonchus* | 0 | 0 | 0 |
| Ruminant2 | *unclassified Teladorsagia* | 0 | 0 | 0 |
| Ruminant3 | *Bunostomum trigonocephalum* | 0.007353 | 0.009938 | 0.00946 |
| Ruminant3 | *Chabertia ovina* | 0.007353 | 0.005723 | 0.001697 |
| Ruminant3 | *Cooperia curticei* | 0.022059 | 0.025823 | 0.024175 |
| Ruminant3 | *Cooperia oncophora* | 0.242647 | 0.279361 | 0.328241 |
| Ruminant3 | *Cooperia pectinata* | 0.007353 | 0.017532 | 0.014718 |
| Ruminant3 | *Cooperia punctata* | 0.018382 | 0.035957 | 0.009564 |
| Ruminant3 | *Cooperia spatulata* | 0.022059 | 0.01689 | 0.010158 |
| Ruminant3 | *Haemonchus contortus* | 0.316176 | 0.266492 | 0.20609 |
| Ruminant3 | *Haemonchus placei* | 0.044118 | 0.063622 | 0.066392 |
| Ruminant3 | *Mecistocirrus digitatus* | 0 | 0.005611 | 0 |
| Ruminant3 | *Oesophagostomum radiatum* | 0.003676 | 0 | 0 |
| Ruminant3 | *Oesophagostomum venulosum* | 0.029412 | 0.021859 | 0.034072 |
| Ruminant3 | *Ostertagia ostertagi* | 0.025735 | 0.013037 | 0.004911 |
| Ruminant3 | *Teladorsagia circumcincta* | 0.088235 | 0.066721 | 0.065105 |
| Ruminant3 | *Trichostrongylus axei* | 0.036765 | 0.037632 | 0.039056 |
| Ruminant3 | *Trichostrongylus colubriformis* | 0.117647 | 0.110913 | 0.140402 |
| Ruminant3 | *Trichostrongylus vitrinus* | 0.011029 | 0.022892 | 0.01486 |
| Ruminant3 | *unclassified Cooperia* | 0 | 0 | 0.031098 |
| Ruminant3 | *unclassified Haemonchus* | 0 | 0 | 0 |
| Ruminant3 | *unclassified Teladorsagia* | 0 | 0 | 0 |
| Ruminant4 | *Bunostomum trigonocephalum* | 0.007353 | 0.014086 | 0.010473 |
| Ruminant4 | *Chabertia ovina* | 0.007353 | 0.007043 | 0.00308 |
| Ruminant4 | *Cooperia curticei* | 0.022059 | 0.021697 | 0.01427 |
| Ruminant4 | *Cooperia oncophora* | 0.242647 | 0.205839 | 0.32816 |
| Ruminant4 | *Cooperia pectinata* | 0.007353 | 0.023174 | 0 |
| Ruminant4 | *Cooperia punctata* | 0.018382 | 0.030558 | 0.00983 |
| Ruminant4 | *Cooperia spatulata* | 0.022059 | 0.024196 | 0.010109 |
| Ruminant4 | *Haemonchus contortus* | 0.316176 | 0.258321 | 0.232486 |
| Ruminant4 | *Haemonchus placei* | 0.044118 | 0.080768 | 0.039729 |
| Ruminant4 | *Mecistocirrus digitatus* | 0 | 0.007838 | 0 |
| Ruminant4 | *Oesophagostomum radiatum* | 0.003676 | 0 | 0 |
| Ruminant4 | *Oesophagostomum venulosum* | 0.029412 | 0.023401 | 0.018124 |
| Ruminant4 | *Ostertagia ostertagi* | 0.025735 | 0.020448 | 0.014665 |
| Ruminant4 | *Teladorsagia circumcincta* | 0.088235 | 0.056117 | 0.074549 |
| Ruminant4 | *Trichostrongylus axei* | 0.036765 | 0.047711 | 0.044554 |
| Ruminant4 | *Trichostrongylus colubriformis* | 0.117647 | 0.146995 | 0.140355 |
| Ruminant4 | *Trichostrongylus vitrinus* | 0.011029 | 0.031807 | 0.015361 |
| Ruminant4 | *unclassified Cooperia* | 0 | 0 | 0.036528 |
| Ruminant4 | *unclassified Haemonchus* | 0 | 0 | 0 |
| Ruminant4 | *unclassified Teladorsagia* | 0 | 0 | 0.007725 |
| Ruminant5 | *Bunostomum trigonocephalum* | 0.007353 | 0.010025 | 0 |
| Ruminant5 | *Chabertia ovina* | 0.007353 | 0.006142 | 0.006016 |
| Ruminant5 | *Cooperia curticei* | 0.022059 | 0.024476 | 0.005659 |
| Ruminant5 | *Cooperia oncophora* | 0.242647 | 0.236317 | 0.368575 |
| Ruminant5 | *Cooperia pectinata* | 0.007353 | 0.017522 | 0 |
| Ruminant5 | *Cooperia punctata* | 0.018382 | 0.036805 | 0.011241 |
| Ruminant5 | *Cooperia spatulata* | 0.022059 | 0.018515 | 0.009467 |
| Ruminant5 | *Haemonchus contortus* | 0.316176 | 0.277276 | 0.226036 |
| Ruminant5 | *Haemonchus placei* | 0.044118 | 0.052339 | 0.074654 |
| Ruminant5 | *Mecistocirrus digitatus* | 0 | 0.005961 | 0 |
| Ruminant5 | *Oesophagostomum radiatum* | 0.003676 | 0 | 0 |
| Ruminant5 | *Oesophagostomum venulosum* | 0.029412 | 0.044256 | 0.016495 |
| Ruminant5 | *Ostertagia ostertagi* | 0.025735 | 0.013638 | 0.006028 |
| Ruminant5 | *Teladorsagia circumcincta* | 0.088235 | 0.065977 | 0.063113 |
| Ruminant5 | *Trichostrongylus axei* | 0.036765 | 0.040553 | 0.03009 |
| Ruminant5 | *Trichostrongylus colubriformis* | 0.117647 | 0.126535 | 0.125035 |
| Ruminant5 | *Trichostrongylus vitrinus* | 0.011029 | 0.023663 | 0.017224 |
| Ruminant5 | *unclassified Cooperia* | 0 | 0 | 0.040367 |
| Ruminant5 | *unclassified Haemonchus* | 0 | 0 | 0 |
| Ruminant5 | *unclassified Teladorsagia* | 0 | 0 | 0 |
| Ruminant6 | *Bunostomum trigonocephalum* | 0.007353 | 0.009238 | 0.005866 |
| Ruminant6 | *Chabertia ovina* | 0.007353 | 0.004914 | 0.00582 |
| Ruminant6 | *Cooperia curticei* | 0.022059 | 0.028923 | 0.015136 |
| Ruminant6 | *Cooperia oncophora* | 0.242647 | 0.263099 | 0.365259 |
| Ruminant6 | *Cooperia pectinata* | 0.007353 | 0.015495 | 0 |
| Ruminant6 | *Cooperia punctata* | 0.018382 | 0.032817 | 0.011029 |
| Ruminant6 | *Cooperia spatulata* | 0.022059 | 0.016865 | 0.011264 |
| Ruminant6 | *Haemonchus contortus* | 0.316176 | 0.261004 | 0.207474 |
| Ruminant6 | *Haemonchus placei* | 0.044118 | 0.060209 | 0.044609 |
| Ruminant6 | *Mecistocirrus digitatus* | 0 | 0.00564 | 0 |
| Ruminant6 | *Oesophagostomum radiatum* | 0.003676 | 0 | 0 |
| Ruminant6 | *Oesophagostomum venulosum* | 0.029412 | 0.044203 | 0.023419 |
| Ruminant6 | *Ostertagia ostertagi* | 0.025735 | 0.013615 | 0.022668 |
| Ruminant6 | *Teladorsagia circumcincta* | 0.088235 | 0.077692 | 0.074399 |
| Ruminant6 | *Trichostrongylus axei* | 0.036765 | 0.042297 | 0.050358 |
| Ruminant6 | *Trichostrongylus colubriformis* | 0.117647 | 0.102908 | 0.105843 |
| Ruminant6 | *Trichostrongylus vitrinus* | 0.011029 | 0.021081 | 0.017071 |
| Ruminant6 | *unclassified Cooperia* | 0 | 0 | 0.039786 |
| Ruminant6 | *unclassified Haemonchus* | 0 | 0 | 0 |
| Ruminant6 | *unclassified Teladorsagia* | 0 | 0 | 0 |
| Ruminant7 | *Bunostomum trigonocephalum* | 0.007353 | 0.015249 | 0.010644 |
| Ruminant7 | *Chabertia ovina* | 0.007353 | 0.007155 | 0 |
| Ruminant7 | *Cooperia curticei* | 0.022059 | 0.024047 | 0.028224 |
| Ruminant7 | *Cooperia oncophora* | 0.242647 | 0.202111 | 0.368092 |
| Ruminant7 | *Cooperia pectinata* | 0.007353 | 0.022405 | 0.005766 |
| Ruminant7 | *Cooperia punctata* | 0.018382 | 0.032845 | 0.010597 |
| Ruminant7 | *Cooperia spatulata* | 0.022059 | 0.024282 | 0.01027 |
| Ruminant7 | *Haemonchus contortus* | 0.316176 | 0.282581 | 0.148722 |
| Ruminant7 | *Haemonchus placei* | 0.044118 | 0.064164 | 0.074706 |
| Ruminant7 | *Mecistocirrus digitatus* | 0 | 0.007859 | 0 |
| Ruminant7 | *Oesophagostomum radiatum* | 0.003676 | 0 | 0 |
| Ruminant7 | *Oesophagostomum venulosum* | 0.029412 | 0.024868 | 0.017112 |
| Ruminant7 | *Ostertagia ostertagi* | 0.025735 | 0.019472 | 0 |
| Ruminant7 | *Teladorsagia circumcincta* | 0.088235 | 0.05783 | 0.072531 |
| Ruminant7 | *Trichostrongylus axei* | 0.036765 | 0.030264 | 0.045359 |
| Ruminant7 | *Trichostrongylus colubriformis* | 0.117647 | 0.151437 | 0.158079 |
| Ruminant7 | *Trichostrongylus vitrinus* | 0.011029 | 0.033431 | 0.016492 |
| Ruminant7 | *unclassified Cooperia* | 0 | 0 | 0.033405 |
| Ruminant7 | *unclassified Haemonchus* | 0 | 0 | 0 |
| Ruminant7 | *unclassified Teladorsagia* | 0 | 0 | 0 |
| Ruminant8 | *Bunostomum trigonocephalum* | 0.007353 | 0.010574 | 0.009435 |
| Ruminant8 | *Chabertia ovina* | 0.007353 | 0.005287 | 0.005117 |
| Ruminant8 | *Cooperia curticei* | 0.022059 | 0.018863 | 0.014242 |
| Ruminant8 | *Cooperia oncophora* | 0.242647 | 0.272754 | 0.326945 |
| Ruminant8 | *Cooperia pectinata* | 0.007353 | 0.016241 | 0 |
| Ruminant8 | *Cooperia punctata* | 0.018382 | 0.024615 | 0.009693 |
| Ruminant8 | *Cooperia spatulata* | 0.022059 | 0.017763 | 0.010108 |
| Ruminant8 | *Haemonchus contortus* | 0.316176 | 0.280705 | 0.227285 |
| Ruminant8 | *Haemonchus placei* | 0.044118 | 0.063864 | 0.052872 |
| Ruminant8 | *Mecistocirrus digitatus* | 0 | 0.005963 | 0 |
| Ruminant8 | *Oesophagostomum radiatum* | 0.003676 | 0 | 0 |
| Ruminant8 | *Oesophagostomum venulosum* | 0.029412 | 0.017806 | 0.030203 |
| Ruminant8 | *Ostertagia ostertagi* | 0.025735 | 0.010743 | 0.014615 |
| Ruminant8 | *Teladorsagia circumcincta* | 0.088235 | 0.07245 | 0.073692 |
| Ruminant8 | *Trichostrongylus axei* | 0.036765 | 0.033878 | 0.046079 |
| Ruminant8 | *Trichostrongylus colubriformis* | 0.117647 | 0.123837 | 0.128896 |
| Ruminant8 | *Trichostrongylus vitrinus* | 0.011029 | 0.024657 | 0.014868 |
| Ruminant8 | *unclassified Cooperia* | 0 | 0 | 0.03595 |
| Ruminant8 | *unclassified Haemonchus* | 0 | 0 | 0 |
| Ruminant8 | *unclassified Teladorsagia* | 0 | 0 | 0 |
| Ruminant9 | *Bunostomum trigonocephalum* | 0.007353 | 0.009225 | 0.00959 |
| Ruminant9 | *Chabertia ovina* | 0.007353 | 0.005055 | 0.003288 |
| Ruminant9 | *Cooperia curticei* | 0.022059 | 0.030465 | 0.0147 |
| Ruminant9 | *Cooperia oncophora* | 0.242647 | 0.264936 | 0.333323 |
| Ruminant9 | *Cooperia pectinata* | 0.007353 | 0.016103 | 0 |
| Ruminant9 | *Cooperia punctata* | 0.018382 | 0.033752 | 0.009853 |
| Ruminant9 | *Cooperia spatulata* | 0.022059 | 0.017346 | 0.010266 |
| Ruminant9 | *Haemonchus contortus* | 0.316176 | 0.268582 | 0.232246 |
| Ruminant9 | *Haemonchus placei* | 0.044118 | 0.062036 | 0.048252 |
| Ruminant9 | *Mecistocirrus digitatus* | 0 | 0.006215 | 0 |
| Ruminant9 | *Oesophagostomum radiatum* | 0.003676 | 0 | 0 |
| Ruminant9 | *Oesophagostomum venulosum* | 0.029412 | 0.033697 | 0.020806 |
| Ruminant9 | *Ostertagia ostertagi* | 0.025735 | 0.012816 | 0.015379 |
| Ruminant9 | *Teladorsagia circumcincta* | 0.088235 | 0.074382 | 0.076096 |
| Ruminant9 | *Trichostrongylus axei* | 0.036765 | 0.036873 | 0.046587 |
| Ruminant9 | *Trichostrongylus colubriformis* | 0.117647 | 0.1054 | 0.119566 |
| Ruminant9 | *Trichostrongylus vitrinus* | 0.011029 | 0.023118 | 0.015187 |
| Ruminant9 | *unclassified Cooperia* | 0 | 0 | 0.037118 |
| Ruminant9 | *unclassified Haemonchus* | 0 | 0 | 0 |
| Ruminant9 | *unclassified Teladorsagia* | 0 | 0 | 0.007743 |

### Table S6. Comparison of relative abundance estimates from QIIME 2 and DADA2 pipelines against ground truth values for the simulated equine nemabiome.

| Sample | Species | Ground | Qiime | dada2 |
| --- | --- | --- | --- | --- |
| Horse1 | Craterostomum acuticaudatum | 0.022222 | 0.021942 | 0.063925 |
| Horse1 | Cyathostomum catinatum | 0.066667 | 0 | 0 |
| Horse1 | Cyathostomum pateratum | 0.044444 | 0.040318 | 0.125544 |
| Horse1 | Cyathostomum tetracanthum | 0.022222 | 0.023587 | 0.126057 |
| Horse1 | Cylicocyclus ashworthi | 0.044444 | 0.033461 | 0 |
| Horse1 | Cylicocyclus auriculatus | 0.033333 | 0.055677 | 0 |
| Horse1 | Cylicocyclus brevicapsulatus | 0.011111 | 0.02057 | 0.062004 |
| Horse1 | Cylicocyclus elongatus | 0.022222 | 0.038124 | 0 |
| Horse1 | Cylicocyclus insigne | 0.122222 | 0.040592 | 0 |
| Horse1 | Cylicocyclus leptostomus | 0.022222 | 0.023039 | 0 |
| Horse1 | Cylicocyclus nassatus | 0.066667 | 0.111355 | 0 |
| Horse1 | Cylicocyclus radiatus | 0.022222 | 0.040044 | 0 |
| Horse1 | Cylicodontophorus bicoronatus | 0.033333 | 0.054855 | 0.181399 |
| Horse1 | Cylicodontophorus mettami | 0.011111 | 0 | 0 |
| Horse1 | Cylicostephanus bidentatus | 0.011111 | 0.02057 | 0.062388 |
| Horse1 | Cylicostephanus calicatus | 0.022222 | 0.018925 | 0 |
| Horse1 | Cylicostephanus goldi | 0.055556 | 0.024685 | 0 |
| Horse1 | Cylicostephanus longibursatus | 0.233333 | 0.25864 | 0 |
| Horse1 | Cylicostephanus minutus | 0.1 | 0.131103 | 0.31527 |
| Horse1 | Cylicostephanus poculatus | 0.011111 | 0.019473 | 0.000897 |
| Horse1 | Gyalocephalus capitatus | 0.011111 | 0.023039 | 0.062516 |
| Horse1 | Petrovinema poculatum | 0.011111 | 0 | 0 |
| Horse1 | unclassified Cylicostephanus | 0 | 0 | 0 |
| Horse2 | Craterostomum acuticaudatum | 0.022222 | 0.017396 | 0.021951 |
| Horse2 | Cyathostomum catinatum | 0.066667 | 0 | 0 |
| Horse2 | Cyathostomum pateratum | 0.044444 | 0.070328 | 0.062715 |
| Horse2 | Cyathostomum tetracanthum | 0.022222 | 0.015905 | 0.043244 |
| Horse2 | Cylicocyclus ashworthi | 0.044444 | 0.034791 | 0.06434 |
| Horse2 | Cylicocyclus auriculatus | 0.033333 | 0.051814 | 0.060279 |
| Horse2 | Cylicocyclus brevicapsulatus | 0.011111 | 0.015408 | 0.021447 |
| Horse2 | Cylicocyclus elongatus | 0.022222 | 0.03417 | 0.035561 |
| Horse2 | Cylicocyclus insigne | 0.122222 | 0.051317 | 0.087938 |
| Horse2 | Cylicocyclus leptostomus | 0.022222 | 0.018762 | 0.020415 |
| Horse2 | Cylicocyclus nassatus | 0.066667 | 0.105492 | 0.116343 |
| Horse2 | Cylicocyclus radiatus | 0.022222 | 0.033797 | 0.042674 |
| Horse2 | Cylicodontophorus bicoronatus | 0.033333 | 0.039637 | 0.063879 |
| Horse2 | Cylicodontophorus mettami | 0.011111 | 0 | 0 |
| Horse2 | Cylicostephanus bidentatus | 0.011111 | 0.018638 | 0.021534 |
| Horse2 | Cylicostephanus calicatus | 0.022222 | 0.017271 | 0.02103 |
| Horse2 | Cylicostephanus goldi | 0.055556 | 0.053057 | 0.063154 |
| Horse2 | Cylicostephanus longibursatus | 0.233333 | 0.263419 | 0.032576 |
| Horse2 | Cylicostephanus minutus | 0.1 | 0.121894 | 0.176863 |
| Horse2 | Cylicostephanus poculatus | 0.011111 | 0.017396 | 0.022369 |
| Horse2 | Gyalocephalus capitatus | 0.011111 | 0.019508 | 0.021622 |
| Horse2 | Petrovinema poculatum | 0.011111 | 0 | 0 |
| Horse2 | unclassified Cylicostephanus | 0 | 0 | 6.59E-05 |
| Horse3 | Craterostomum acuticaudatum | 0.022222 | 0.017915 | 0.003684 |
| Horse3 | Cyathostomum catinatum | 0.066667 | 0 | 0 |
| Horse3 | Cyathostomum pateratum | 0.044444 | 0.083708 | 0 |
| Horse3 | Cyathostomum tetracanthum | 0.022222 | 0.034785 | 0.123119 |
| Horse3 | Cylicocyclus ashworthi | 0.044444 | 0.033821 | 0 |
| Horse3 | Cylicocyclus auriculatus | 0.033333 | 0.050691 | 0 |
| Horse3 | Cylicocyclus brevicapsulatus | 0.011111 | 0.014781 | 0.084972 |
| Horse3 | Cylicocyclus elongatus | 0.022222 | 0.033901 | 0 |
| Horse3 | Cylicocyclus insigne | 0.122222 | 0.064428 | 0 |
| Horse3 | Cylicocyclus leptostomus | 0.022222 | 0.014942 | 0 |
| Horse3 | Cylicocyclus nassatus | 0.066667 | 0.084271 | 0 |
| Horse3 | Cylicocyclus radiatus | 0.022222 | 0.03117 | 0 |
| Horse3 | Cylicodontophorus bicoronatus | 0.033333 | 0.033419 | 0.135481 |
| Horse3 | Cylicodontophorus mettami | 0.011111 | 0 | 0 |
| Horse3 | Cylicostephanus bidentatus | 0.011111 | 0.017513 | 0 |
| Horse3 | Cylicostephanus calicatus | 0.022222 | 0.015424 | 0 |
| Horse3 | Cylicostephanus goldi | 0.055556 | 0.048924 | 0 |
| Horse3 | Cylicostephanus longibursatus | 0.233333 | 0.249679 | 0.025723 |
| Horse3 | Cylicostephanus minutus | 0.1 | 0.135684 | 0.627021 |
| Horse3 | Cylicostephanus poculatus | 0.011111 | 0.018557 | 0 |
| Horse3 | Gyalocephalus capitatus | 0.011111 | 0.016388 | 0 |
| Horse3 | Petrovinema poculatum | 0.011111 | 0 | 0 |
| Horse3 | unclassified Cylicostephanus | 0 | 0 | 0 |
| Horse4 | Craterostomum acuticaudatum | 0.022222 | 0.019105 | 0.021167 |
| Horse4 | Cyathostomum catinatum | 0.066667 | 0 | 0.071934 |
| Horse4 | Cyathostomum pateratum | 0.044444 | 0.043395 | 0.05278 |
| Horse4 | Cyathostomum tetracanthum | 0.022222 | 0.018286 | 0.041974 |
| Horse4 | Cylicocyclus ashworthi | 0.044444 | 0.042849 | 0.062378 |
| Horse4 | Cylicocyclus auriculatus | 0.033333 | 0.051583 | 0.047229 |
| Horse4 | Cylicocyclus brevicapsulatus | 0.011111 | 0.020469 | 0.02087 |
| Horse4 | Cylicocyclus elongatus | 0.022222 | 0.037391 | 0.039664 |
| Horse4 | Cylicocyclus insigne | 0.122222 | 0.059498 | 0.061785 |
| Horse4 | Cylicocyclus leptostomus | 0.022222 | 0.021015 | 0.017501 |
| Horse4 | Cylicocyclus nassatus | 0.066667 | 0.1119 | 0.096851 |
| Horse4 | Cylicocyclus radiatus | 0.022222 | 0.039028 | 0.021019 |
| Horse4 | Cylicodontophorus bicoronatus | 0.033333 | 0.06059 | 0.062018 |
| Horse4 | Cylicodontophorus mettami | 0.011111 | 0 | 0 |
| Horse4 | Cylicostephanus bidentatus | 0.011111 | 0.019378 | 0.02087 |
| Horse4 | Cylicostephanus calicatus | 0.022222 | 0.020469 | 0.018963 |
| Horse4 | Cylicostephanus goldi | 0.055556 | 0.01774 | 0.061509 |
| Horse4 | Cylicostephanus longibursatus | 0.233333 | 0.251365 | 0.074244 |
| Horse4 | Cylicostephanus minutus | 0.1 | 0.129367 | 0.164739 |
| Horse4 | Cylicostephanus poculatus | 0.011111 | 0.019105 | 0.021485 |
| Horse4 | Gyalocephalus capitatus | 0.011111 | 0.017467 | 0.021019 |
| Horse4 | Petrovinema poculatum | 0.011111 | 0 | 0 |
| Horse4 | unclassified Cylicostephanus | 0 | 0 | 0 |
| Horse5 | Craterostomum acuticaudatum | 0.022222 | 0.019051 | 0.088433 |
| Horse5 | Cyathostomum catinatum | 0.066667 | 0 | 0 |
| Horse5 | Cyathostomum pateratum | 0.044444 | 0.087266 | 0 |
| Horse5 | Cyathostomum tetracanthum | 0.022222 | 0.016101 | 0.001418 |
| Horse5 | Cylicocyclus ashworthi | 0.044444 | 0.033432 | 0 |
| Horse5 | Cylicocyclus auriculatus | 0.033333 | 0.049656 | 0 |
| Horse5 | Cylicocyclus brevicapsulatus | 0.011111 | 0.016962 | 0.081876 |
| Horse5 | Cylicocyclus elongatus | 0.022222 | 0.030728 | 0 |
| Horse5 | Cylicocyclus insigne | 0.122222 | 0.072886 | 0 |
| Horse5 | Cylicocyclus leptostomus | 0.022222 | 0.014135 | 0 |
| Horse5 | Cylicocyclus nassatus | 0.066667 | 0.091937 | 0 |
| Horse5 | Cylicocyclus radiatus | 0.022222 | 0.035767 | 0 |
| Horse5 | Cylicodontophorus bicoronatus | 0.033333 | 0.036136 | 0.127422 |
| Horse5 | Cylicodontophorus mettami | 0.011111 | 0 | 0 |
| Horse5 | Cylicostephanus bidentatus | 0.011111 | 0.018559 | 0 |
| Horse5 | Cylicostephanus calicatus | 0.022222 | 0.019297 | 0 |
| Horse5 | Cylicostephanus goldi | 0.055556 | 0.054326 | 0 |
| Horse5 | Cylicostephanus longibursatus | 0.233333 | 0.252458 | 0.024102 |
| Horse5 | Cylicostephanus minutus | 0.1 | 0.123279 | 0.593691 |
| Horse5 | Cylicostephanus poculatus | 0.011111 | 0.013889 | 0.083058 |
| Horse5 | Gyalocephalus capitatus | 0.011111 | 0.014135 | 0 |
| Horse5 | Petrovinema poculatum | 0.011111 | 0 | 0 |
| Horse5 | unclassified Cylicostephanus | 0 | 0 | 0 |
| Horse6 | Craterostomum acuticaudatum | 0.022222 | 0.016385 | 0.052055 |
| Horse6 | Cyathostomum catinatum | 0.066667 | 0 | 0 |
| Horse6 | Cyathostomum pateratum | 0.044444 | 0.082091 | 0.062278 |
| Horse6 | Cyathostomum tetracanthum | 0.022222 | 0.015644 | 0.07271 |
| Horse6 | Cylicocyclus ashworthi | 0.044444 | 0.0359 | 0.032026 |
| Horse6 | Cylicocyclus auriculatus | 0.033333 | 0.055414 | 0 |
| Horse6 | Cylicocyclus brevicapsulatus | 0.011111 | 0.019597 | 0 |
| Horse6 | Cylicocyclus elongatus | 0.022222 | 0.032853 | 0.023576 |
| Horse6 | Cylicocyclus insigne | 0.122222 | 0.066365 | 0.024932 |
| Horse6 | Cylicocyclus leptostomus | 0.022222 | 0.018197 | 0.005529 |
| Horse6 | Cylicocyclus nassatus | 0.066667 | 0.083903 | 0.066973 |
| Horse6 | Cylicocyclus radiatus | 0.022222 | 0.033512 | 0.00918 |
| Horse6 | Cylicodontophorus bicoronatus | 0.033333 | 0.036888 | 0.108492 |
| Horse6 | Cylicodontophorus mettami | 0.011111 | 0 | 0 |
| Horse6 | Cylicostephanus bidentatus | 0.011111 | 0.017373 | 0 |
| Horse6 | Cylicostephanus calicatus | 0.022222 | 0.017291 | 0.01231 |
| Horse6 | Cylicostephanus goldi | 0.055556 | 0.050391 | 0.021281 |
| Horse6 | Cylicostephanus longibursatus | 0.233333 | 0.248827 | 0.054037 |
| Horse6 | Cylicostephanus minutus | 0.1 | 0.136764 | 0.353536 |
| Horse6 | Cylicostephanus poculatus | 0.011111 | 0.015809 | 0.051951 |
| Horse6 | Gyalocephalus capitatus | 0.011111 | 0.016797 | 0.049134 |
| Horse6 | Petrovinema poculatum | 0.011111 | 0 | 0 |
| Horse6 | unclassified Cylicostephanus | 0 | 0 | 0 |
| Horse7 | Craterostomum acuticaudatum | 0.022222 | 0.021767 | 0.026918 |
| Horse7 | Cyathostomum catinatum | 0.066667 | 0.029107 | 0.04898 |
| Horse7 | Cyathostomum pateratum | 0.044444 | 0.068337 | 0 |
| Horse7 | Cyathostomum tetracanthum | 0.022222 | 0.022273 | 0.053134 |
| Horse7 | Cylicocyclus ashworthi | 0.044444 | 0.02936 | 0.079512 |
| Horse7 | Cylicocyclus auriculatus | 0.033333 | 0.050873 | 0.062844 |
| Horse7 | Cylicocyclus brevicapsulatus | 0.011111 | 0.01797 | 0.026432 |
| Horse7 | Cylicocyclus elongatus | 0.022222 | 0.037206 | 0.051084 |
| Horse7 | Cylicocyclus insigne | 0.122222 | 0.03594 | 0.082371 |
| Horse7 | Cylicocyclus leptostomus | 0.022222 | 0.016958 | 0.021847 |
| Horse7 | Cylicocyclus nassatus | 0.066667 | 0.107821 | 0.107239 |
| Horse7 | Cylicocyclus radiatus | 0.022222 | 0.018983 | 0.024922 |
| Horse7 | Cylicodontophorus bicoronatus | 0.033333 | 0.056188 | 0.078164 |
| Horse7 | Cylicodontophorus mettami | 0.011111 | 0 | 0 |
| Horse7 | Cylicostephanus bidentatus | 0.011111 | 0.019489 | 0.026648 |
| Horse7 | Cylicostephanus calicatus | 0.022222 | 0.018983 | 0.023627 |
| Horse7 | Cylicostephanus goldi | 0.055556 | 0.052645 | 0.078326 |
| Horse7 | Cylicostephanus longibursatus | 0.233333 | 0.226525 | 0.049034 |
| Horse7 | Cylicostephanus minutus | 0.1 | 0.139205 | 0.105135 |
| Horse7 | Cylicostephanus poculatus | 0.011111 | 0.015692 | 0.027187 |
| Horse7 | Gyalocephalus capitatus | 0.011111 | 0.01468 | 0.026594 |
| Horse7 | Petrovinema poculatum | 0.011111 | 0 | 0 |
| Horse7 | unclassified Cylicostephanus | 0 | 0 | 0 |
| Horse8 | Craterostomum acuticaudatum | 0.022222 | 0.017217 | 0.10964 |
| Horse8 | Cyathostomum catinatum | 0.066667 | 0 | 0 |
| Horse8 | Cyathostomum pateratum | 0.044444 | 0.081659 | 0 |
| Horse8 | Cyathostomum tetracanthum | 0.022222 | 0.017814 | 0 |
| Horse8 | Cylicocyclus ashworthi | 0.044444 | 0.034792 | 0 |
| Horse8 | Cylicocyclus auriculatus | 0.033333 | 0.050215 | 0 |
| Horse8 | Cylicocyclus brevicapsulatus | 0.011111 | 0.017456 | 0.10214 |
| Horse8 | Cylicocyclus elongatus | 0.022222 | 0.035988 | 0 |
| Horse8 | Cylicocyclus insigne | 0.122222 | 0.051052 | 0 |
| Horse8 | Cylicocyclus leptostomus | 0.022222 | 0.01889 | 0 |
| Horse8 | Cylicocyclus nassatus | 0.066667 | 0.095648 | 0 |
| Horse8 | Cylicocyclus radiatus | 0.022222 | 0.03527 | 0 |
| Horse8 | Cylicodontophorus bicoronatus | 0.033333 | 0.049976 | 0 |
| Horse8 | Cylicodontophorus mettami | 0.011111 | 0 | 0 |
| Horse8 | Cylicostephanus bidentatus | 0.011111 | 0.017456 | 0 |
| Horse8 | Cylicostephanus calicatus | 0.022222 | 0.01889 | 0 |
| Horse8 | Cylicostephanus goldi | 0.055556 | 0.050335 | 0 |
| Horse8 | Cylicostephanus longibursatus | 0.233333 | 0.248326 | 0.013457 |
| Horse8 | Cylicostephanus minutus | 0.1 | 0.127331 | 0.73351 |
| Horse8 | Cylicostephanus poculatus | 0.011111 | 0.01638 | 0.041253 |
| Horse8 | Gyalocephalus capitatus | 0.011111 | 0.015304 | 0 |
| Horse8 | Petrovinema poculatum | 0.011111 | 0 | 0 |
| Horse8 | unclassified Cylicostephanus | 0 | 0 | 0 |
| Horse9 | Craterostomum acuticaudatum | 0.022222 | 0.014617 | 0.051654 |
| Horse9 | Cyathostomum catinatum | 0.066667 | 0.025276 | 0 |
| Horse9 | Cyathostomum pateratum | 0.044444 | 0.076513 | 0 |
| Horse9 | Cyathostomum tetracanthum | 0.022222 | 0.031062 | 0.001988 |
| Horse9 | Cylicocyclus ashworthi | 0.044444 | 0.044766 | 0.132682 |
| Horse9 | Cylicocyclus auriculatus | 0.033333 | 0.046974 | 0 |
| Horse9 | Cylicocyclus brevicapsulatus | 0.011111 | 0.015455 | 0.049152 |
| Horse9 | Cylicocyclus elongatus | 0.022222 | 0.031519 | 0 |
| Horse9 | Cylicocyclus insigne | 0.122222 | 0.063723 | 0 |
| Horse9 | Cylicocyclus leptostomus | 0.022222 | 0.016216 | 0 |
| Horse9 | Cylicocyclus nassatus | 0.066667 | 0.095242 | 0.151911 |
| Horse9 | Cylicocyclus radiatus | 0.022222 | 0.030986 | 0 |
| Horse9 | Cylicodontophorus bicoronatus | 0.033333 | 0.034336 | 0.111362 |
| Horse9 | Cylicodontophorus mettami | 0.011111 | 0 | 0 |
| Horse9 | Cylicostephanus bidentatus | 0.011111 | 0.016216 | 0 |
| Horse9 | Cylicostephanus calicatus | 0.022222 | 0.017054 | 0 |
| Horse9 | Cylicostephanus goldi | 0.055556 | 0.04842 | 0 |
| Horse9 | Cylicostephanus longibursatus | 0.233333 | 0.233727 | 0.017275 |
| Horse9 | Cylicostephanus minutus | 0.1 | 0.124553 | 0.380566 |
| Horse9 | Cylicostephanus poculatus | 0.011111 | 0.01713 | 0.052579 |
| Horse9 | Gyalocephalus capitatus | 0.011111 | 0.016216 | 0.050831 |
| Horse9 | Petrovinema poculatum | 0.011111 | 0 | 0 |
| Horse9 | unclassified Cylicostephanus | 0 | 0 | 0 |

### Table S7: Taxonomic classification accuracy of NCBI SRA nemabiome communities following processing with QIIME 2 and DADA2 pipelines. Ground truth is not known.

| Host | Analysis | Kingdom | Phylum | Class | Order | Family | Genus | Species |
| --- | --- | --- | --- | --- | --- | --- | --- | --- |
| Bison | QIIME2 | 1 | 1 | 1 | 1 | 5 | 6 | 8 |
| Bison | R DADA2 | 1 | 1 | 1 | 1 | 5 | 5 | 9 |
| Camel | R DADA2 | 1 | 1 | 1 | 1 | 3 | 4 | 6 |
| Camel | QIIME2 | 1 | 1 | 1 | 1 | 5 | 6 | 8 |
| Cattle | QIIME2 | 1 | 1 | 1 | 1 | 4 | 5 | 10 |
| Cattle | R DADA2 | 1 | 1 | 1 | 1 | 4 | 5 | 11 |
| Dog | QIIME2 | 1 | 1 | 1 | 1 | 1 | 2 | 2 |
| Dog | R DADA2 | 1 | 1 | 1 | 1 | 1 | 2 | 3 |
| Goat | QIIME2 | 1 | 1 | 1 | 1 | 4 | 6 | 9 |
| Goat | R DADA2 | 1 | 1 | 1 | 1 | 4 | 5 | 9 |
| Horse | R DADA2 | 1 | 1 | 1 | 1 | 1 | 6 | 16 |
| Horse | QIIME2 | 1 | 1 | 1 | 1 | 1 | 9 | 22 |
| Kangaroo | QIIME2 | 1 | 1 | 1 | 2 | 2 | 8 | 10 |
| Kangaroo | R DADA2 | 1 | 1 | 1 | 1 | 2 | 8 | 11 |
| Moose | R DADA2 | 1 | 1 | 1 | 1 | 6 | 7 | 9 |
| Moose | QIIME2 | 1 | 1 | 1 | 2 | 7 | 8 | 16 |
| Pig | R DADA2 | 1 | 1 | 1 | 2 | 5 | 6 | 7 |
| Pig | QIIME2 | 1 | 1 | 1 | 2 | 8 | 8 | 8 |
| Sheep | QIIME2 | 1 | 1 | 1 | 2 | 5 | 8 | 13 |
| Sheep | R DADA2 | 1 | 1 | 1 | 1 | 6 | 9 | 17 |


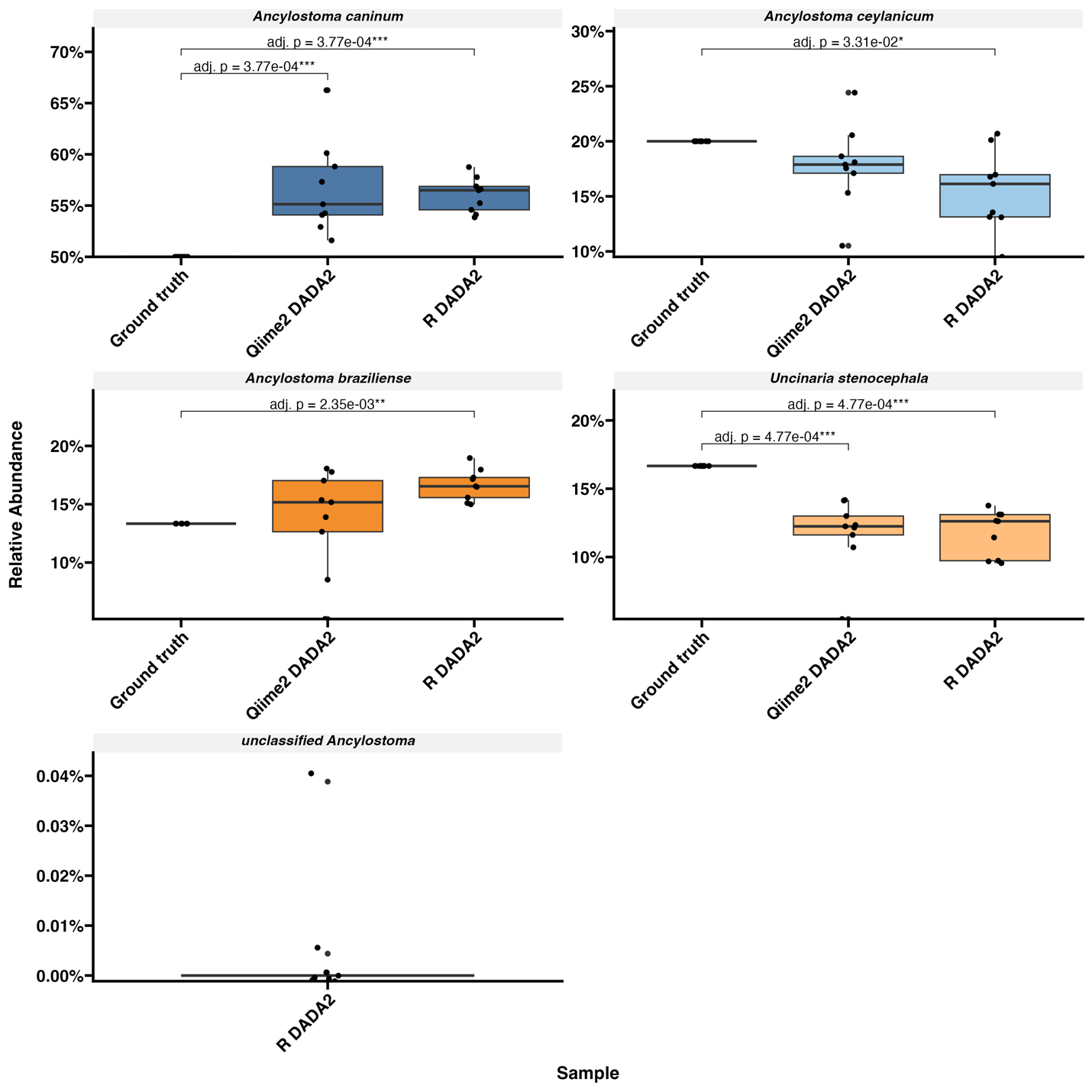


### Figure S1: Pairwise comparison of the simulated canine nemabiome.


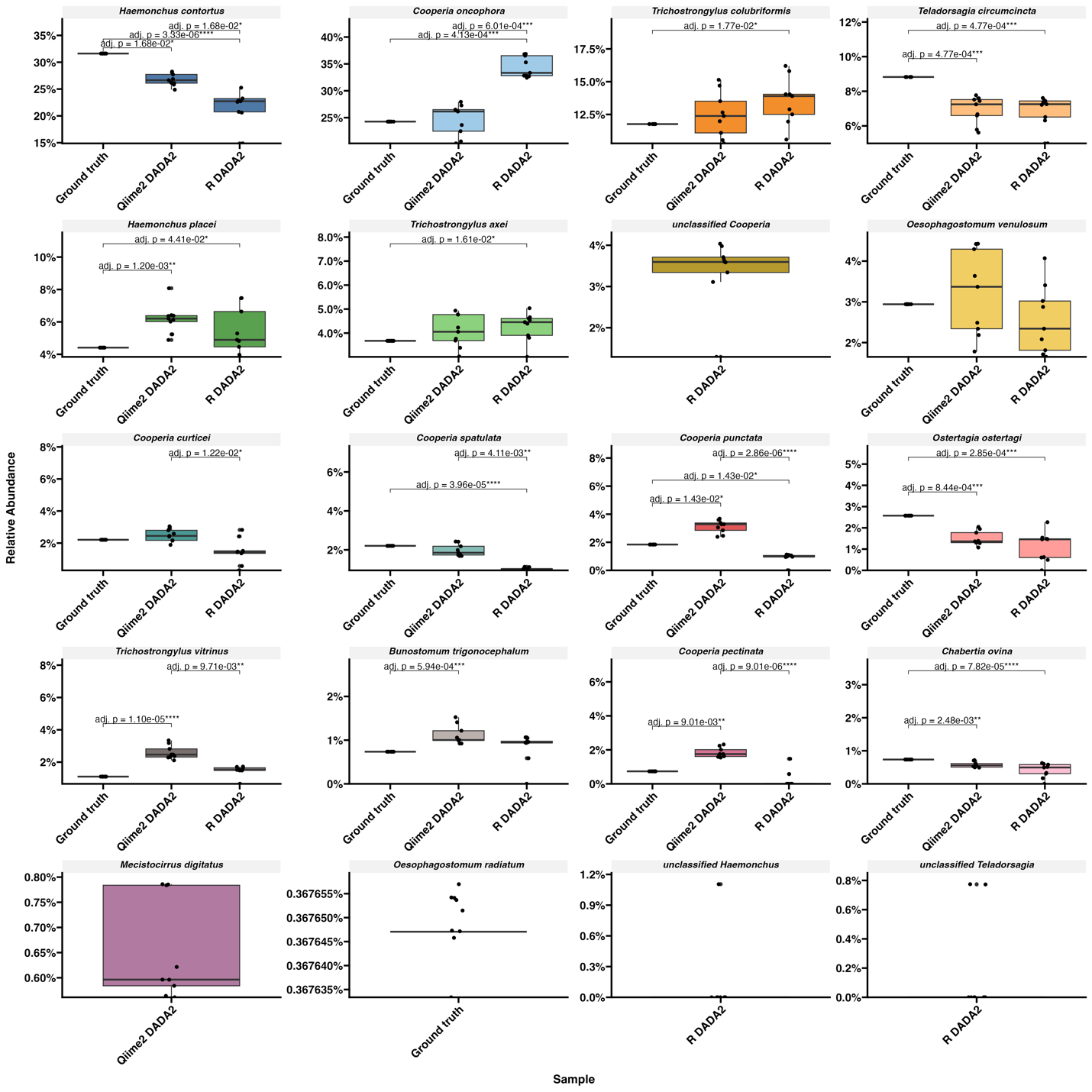


### Figure S2 : Pairwise comparison of the simulated ruminant nemabiome


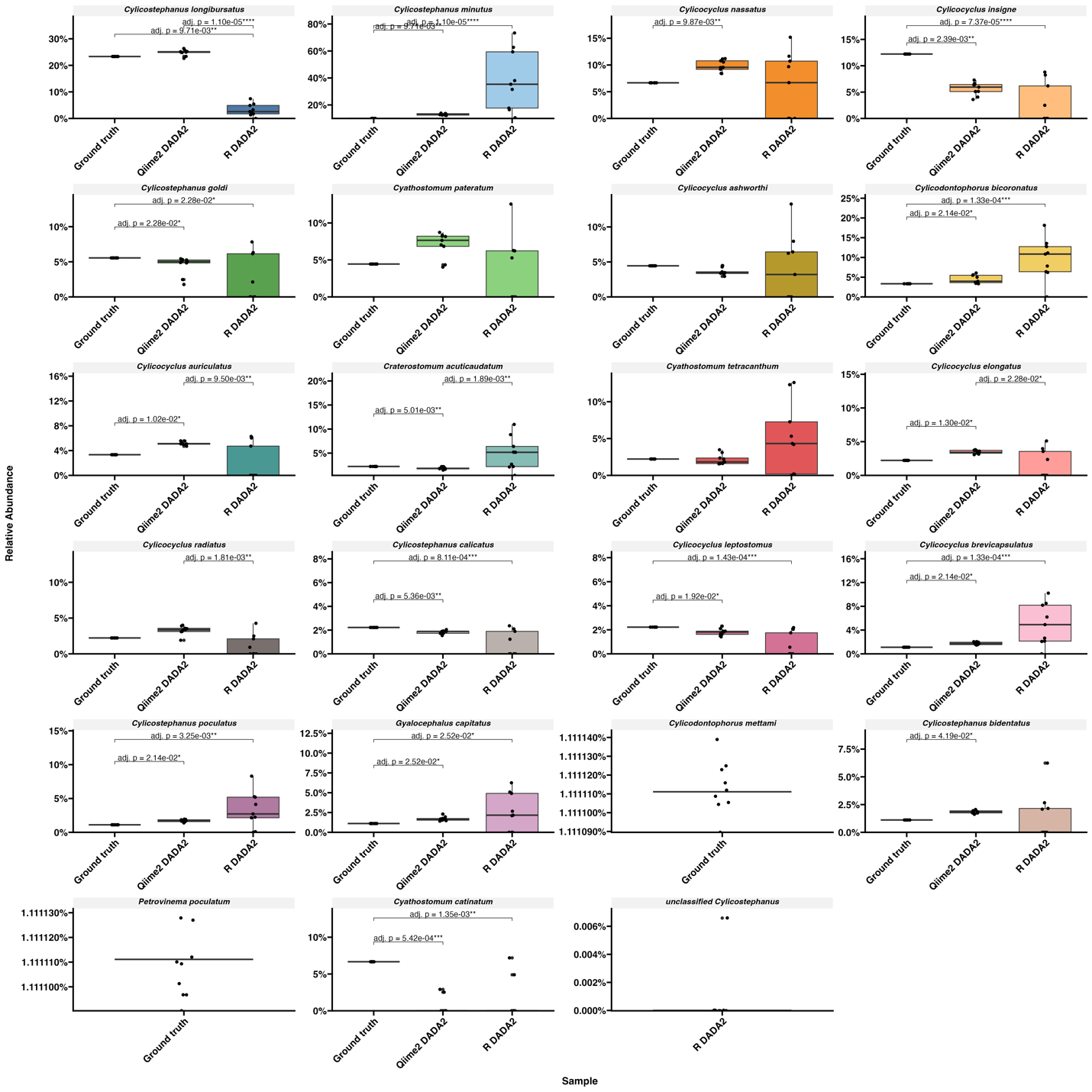


### Figure S3: Pairwise comparison of the simulated equine nemabiome.


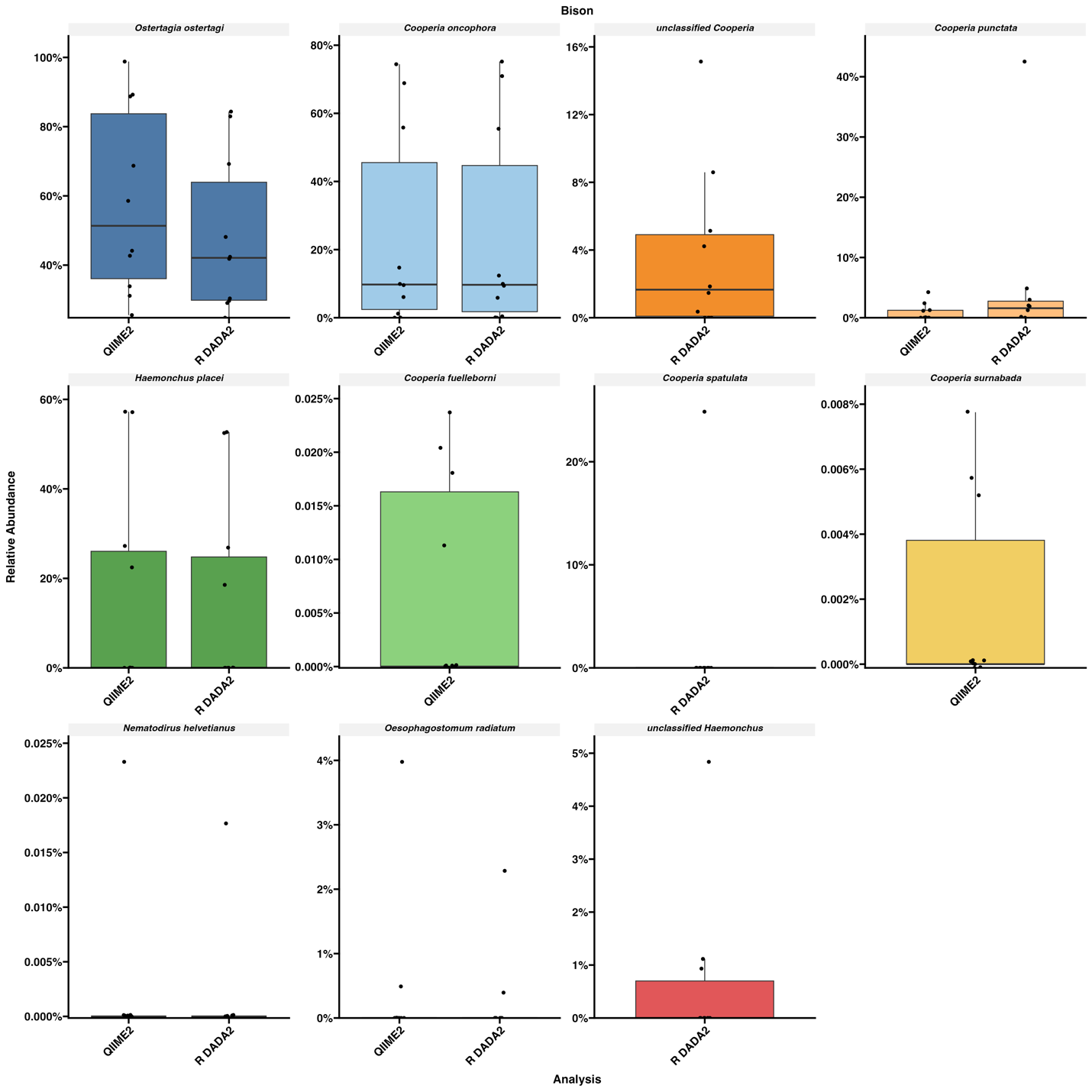


### Figure S4: Pairwise comparison of the NCBI SRA bison dataset.


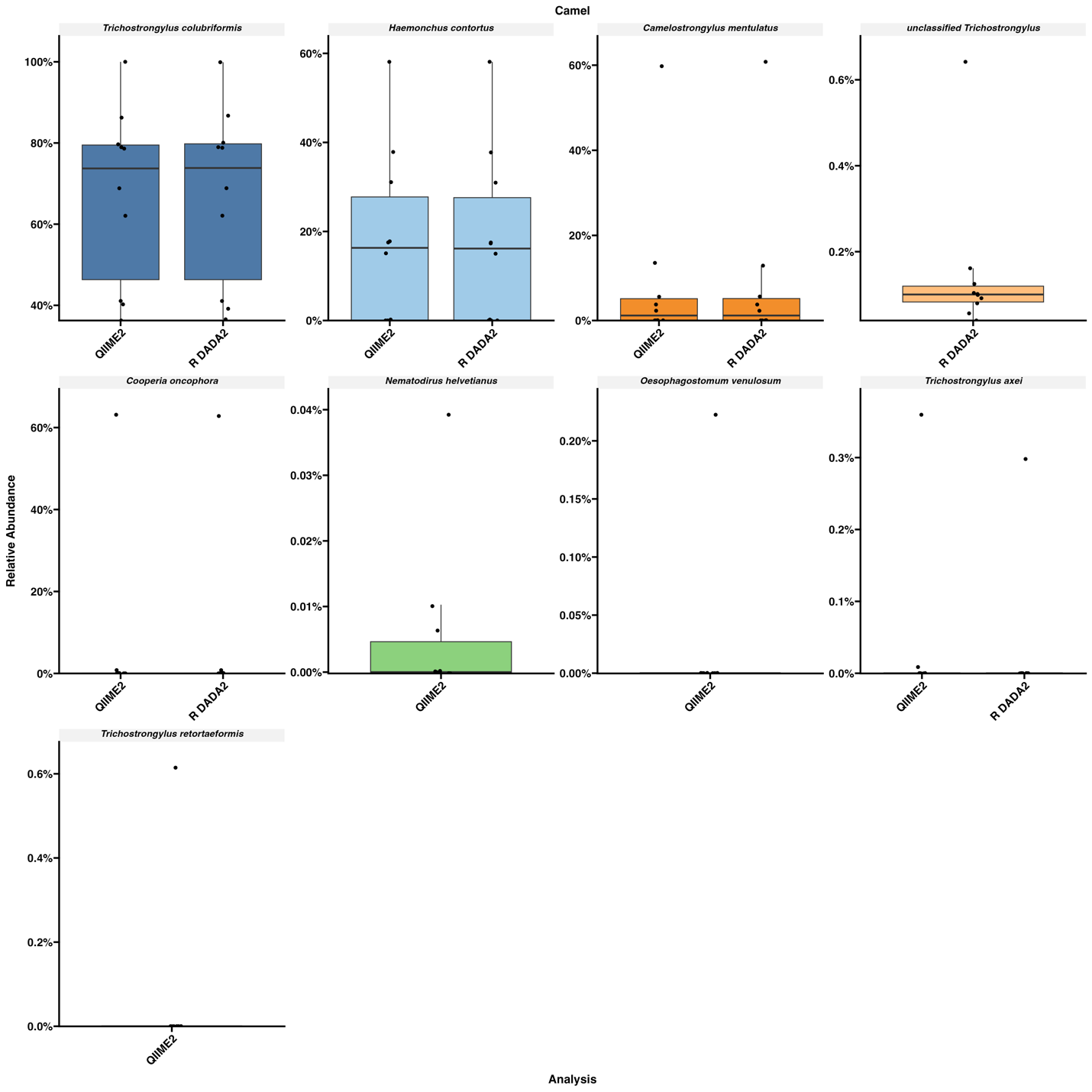


### Figure S5: Pairwise comparison of the NCBI SRA camel dataset.


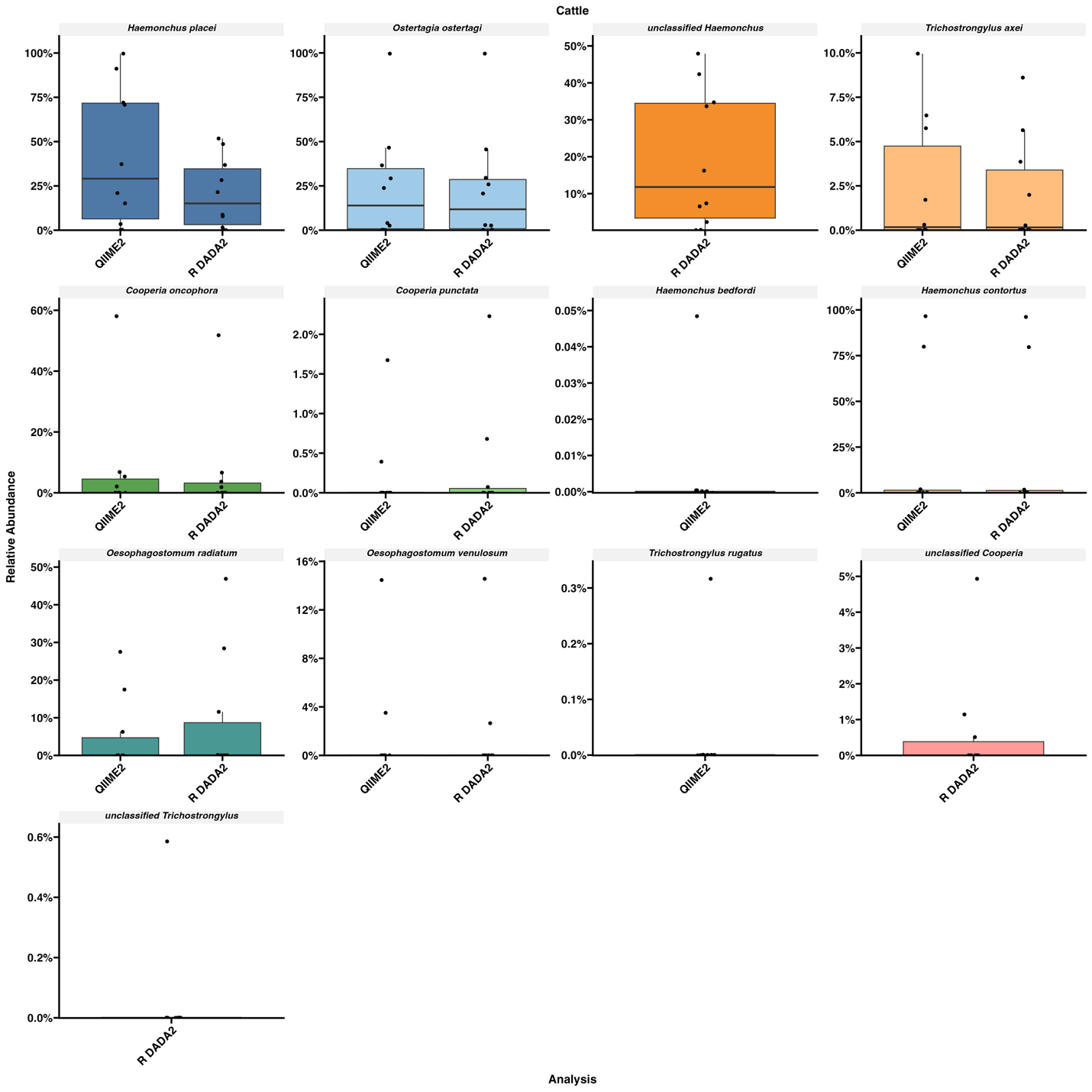


### Figure S6: Pairwise comparison of the NCBI SRA cattle dataset.


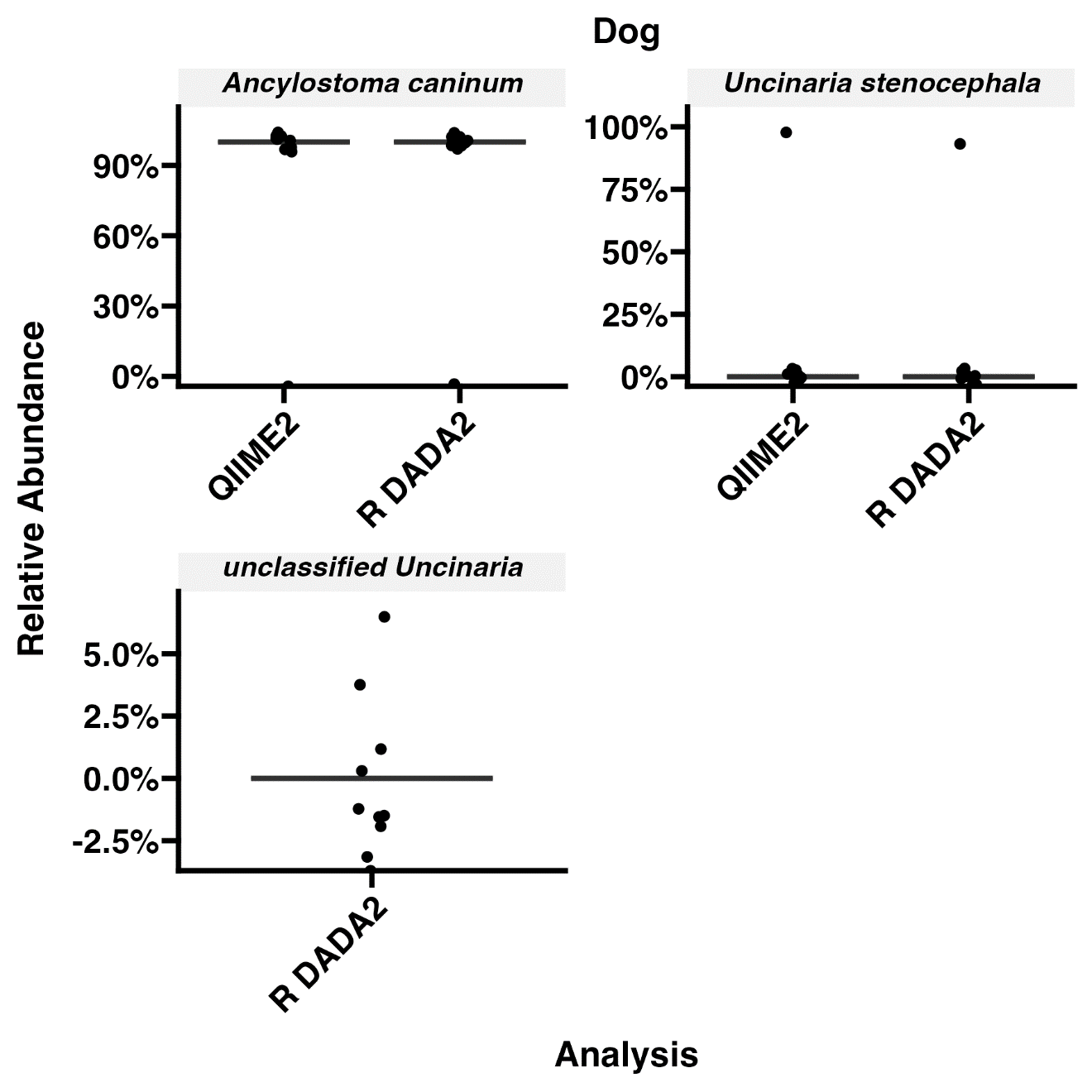


### Figure S7: Pairwise comparison of the NCBI SRA dog dataset.


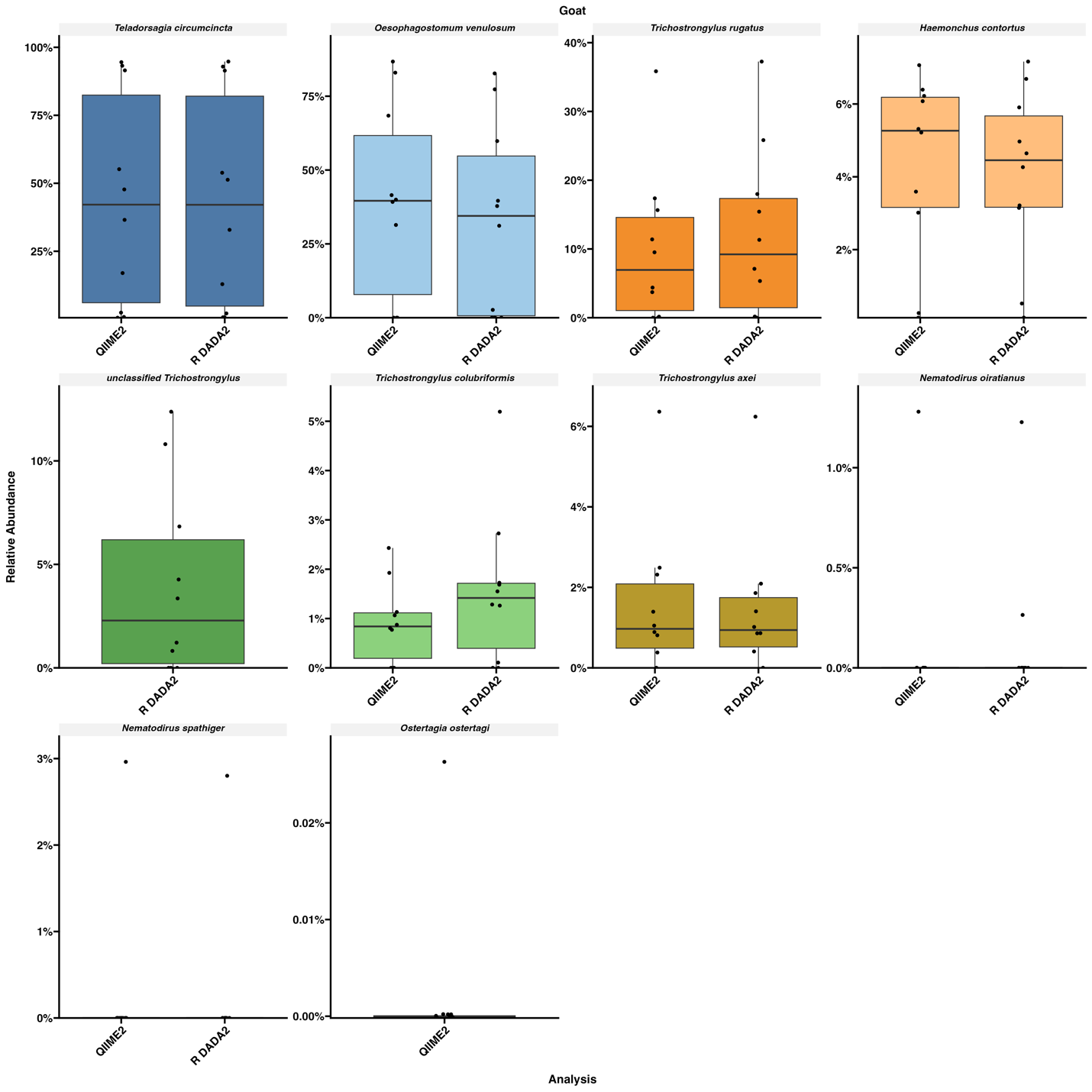


### Figure S8: Pairwise comparison of the NCBI SRA goat dataset.


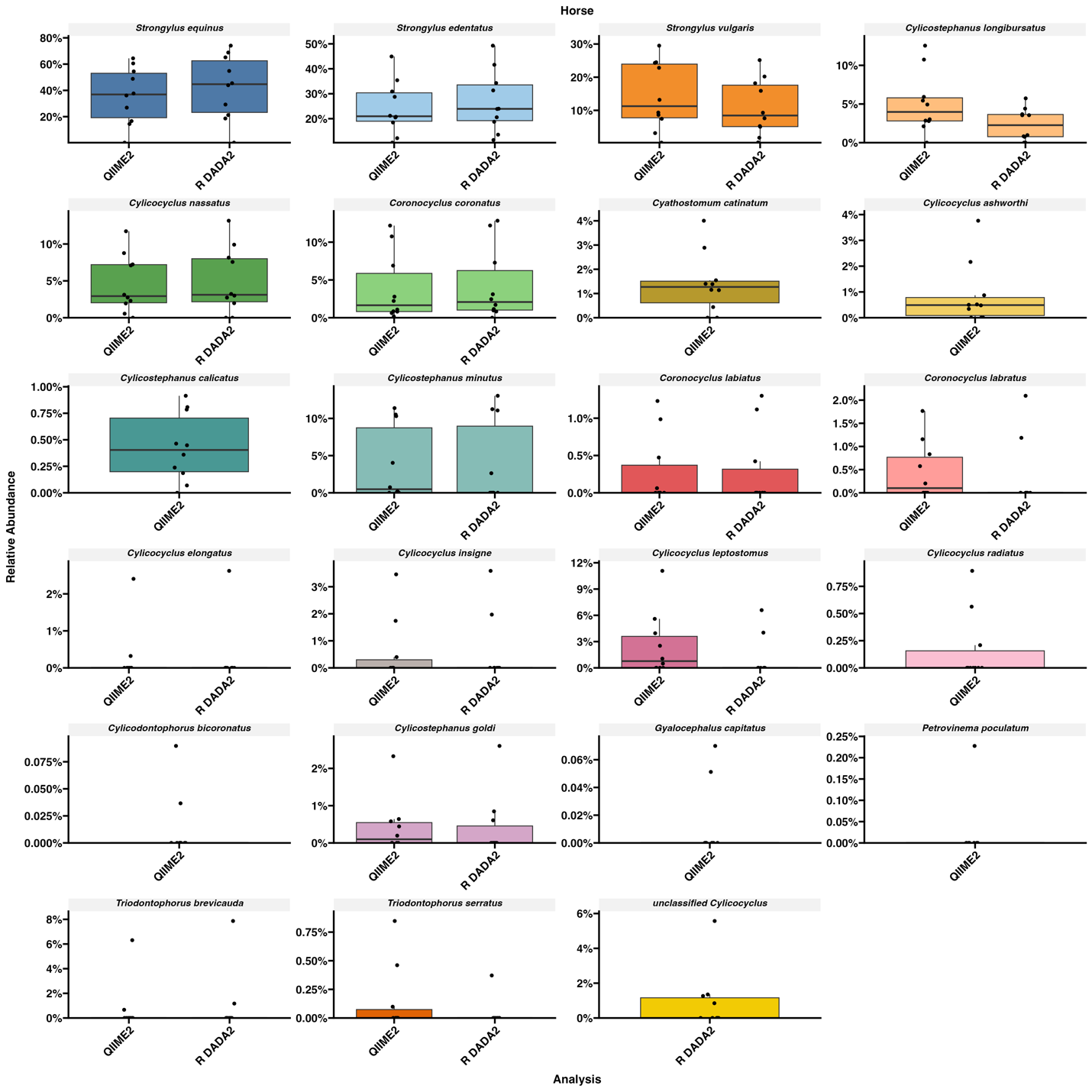


### Figure S9: Pairwise comparison of the NCBI SRA horse dataset.


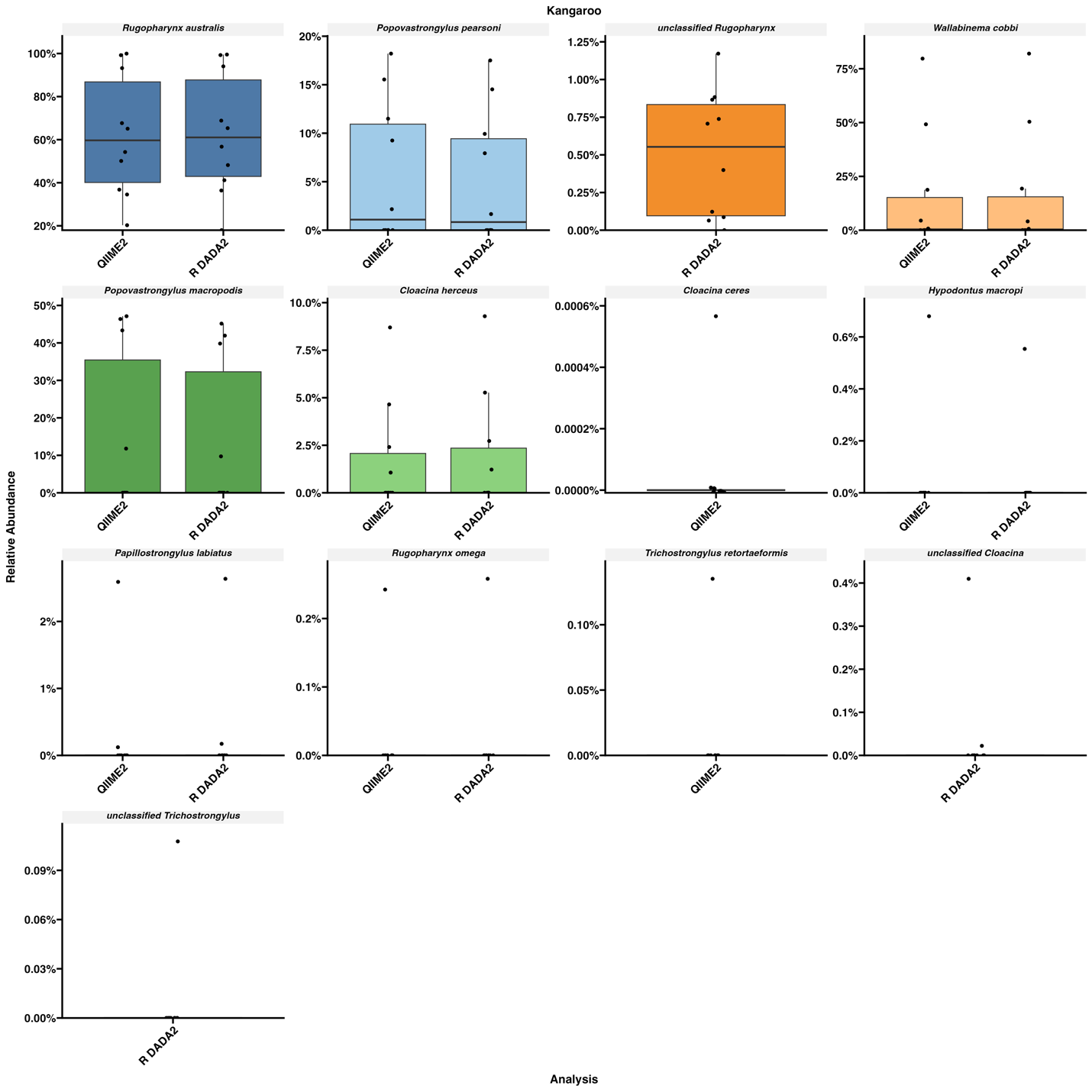


### Figure S10: Pairwise comparison of the NCBI SRA kangaroo dataset.


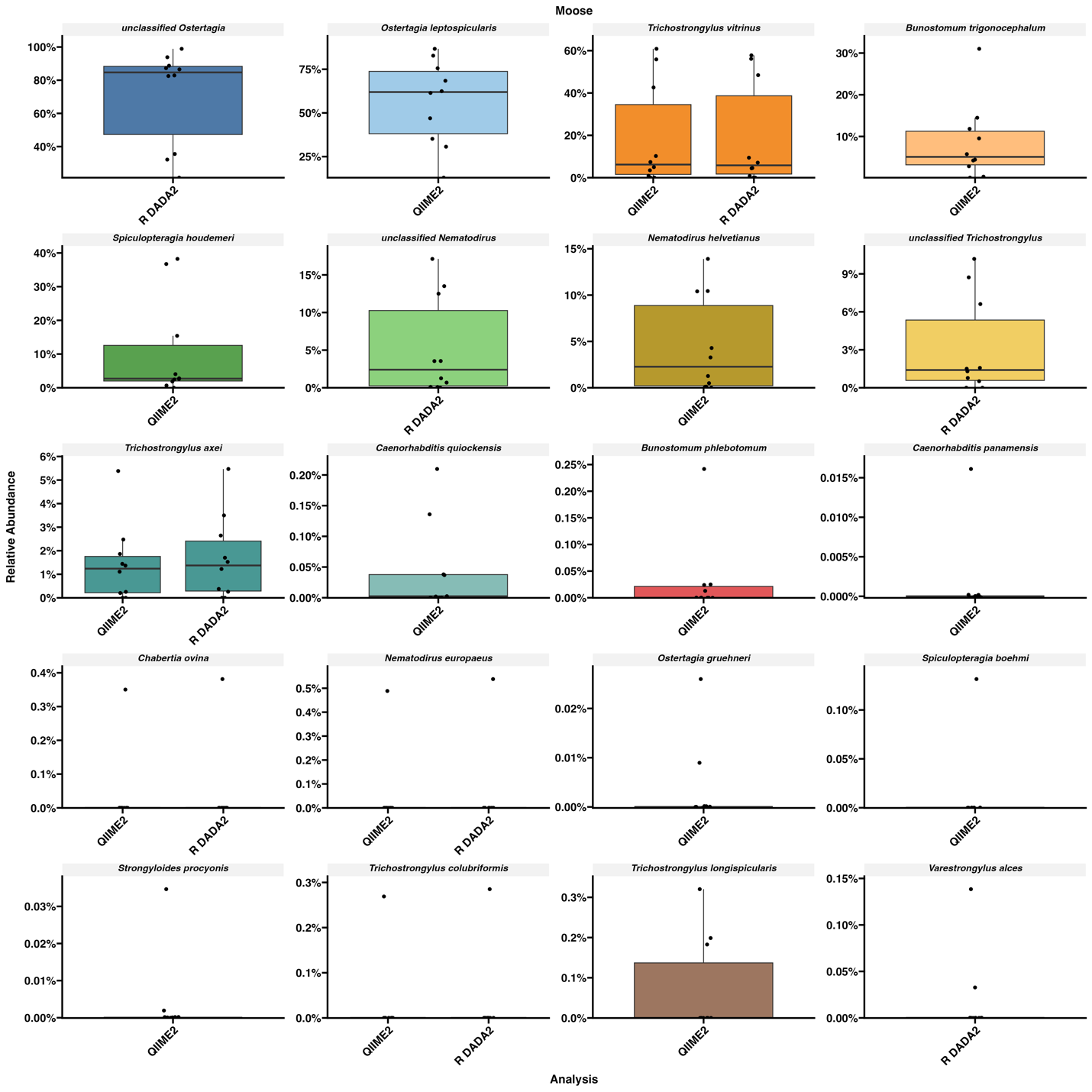


### Figure S11: Pairwise comparison of the NCBI SRA moose dataset.


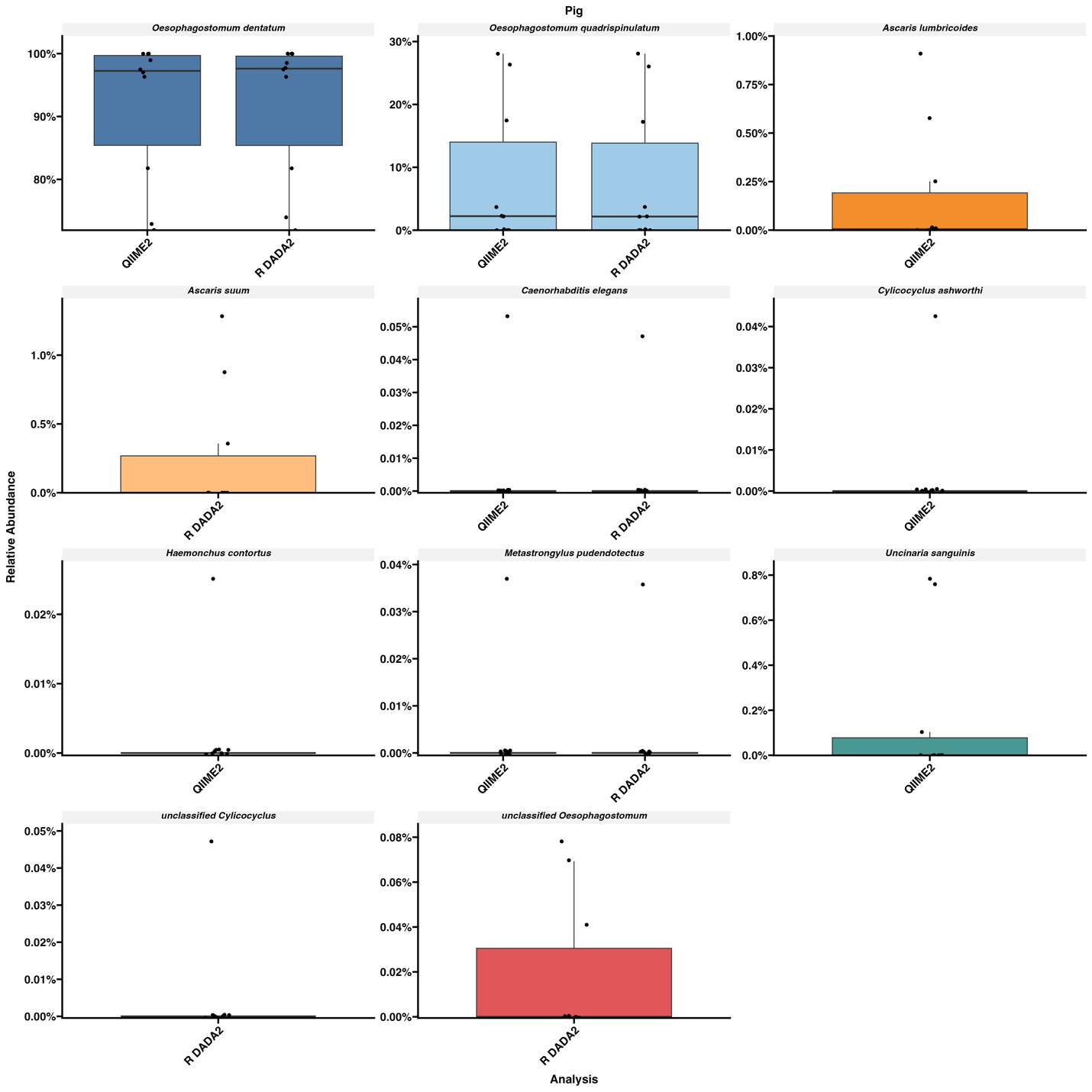


### Figure S12: Pairwise comparison of the NCBI SRA pig dataset.


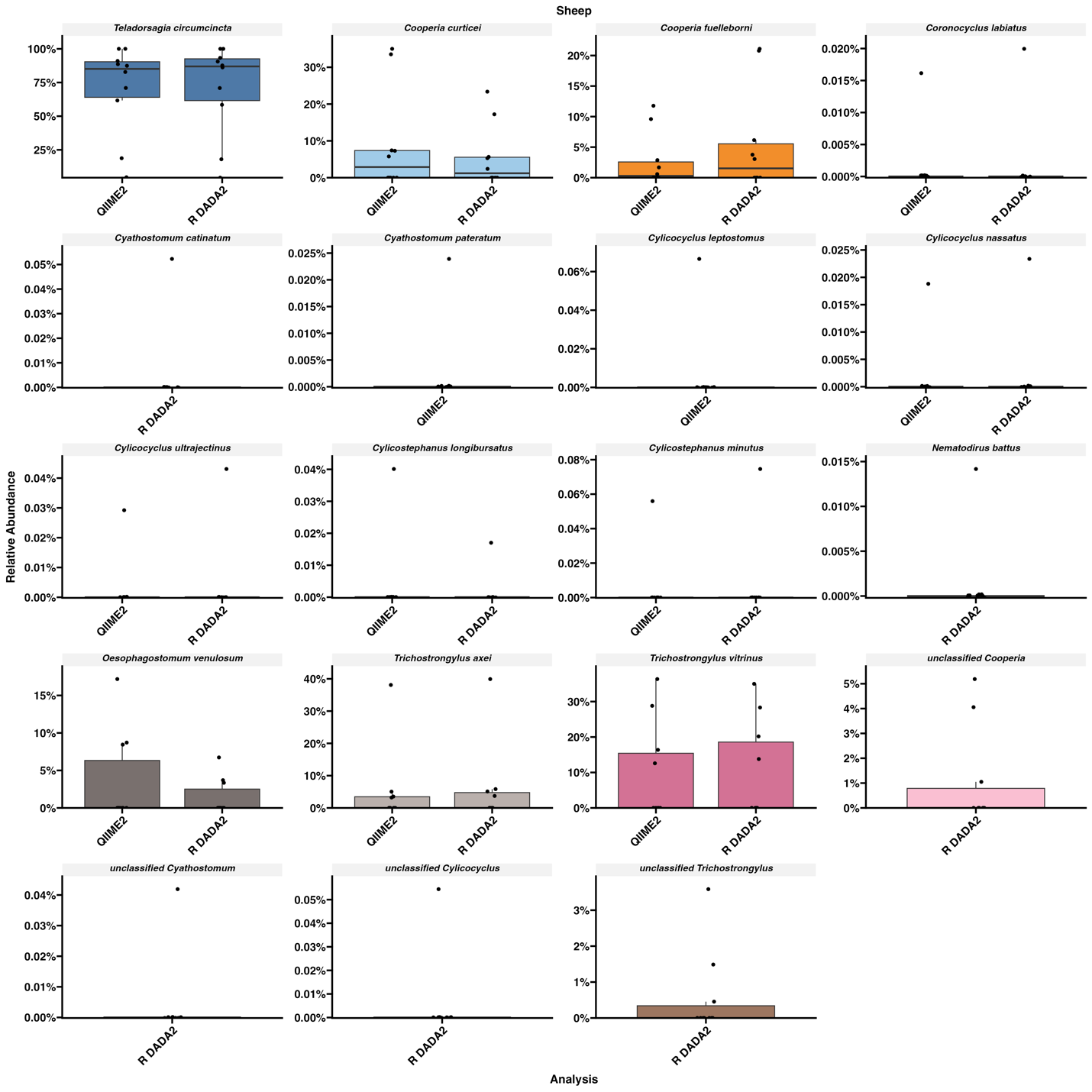


### Figure S13: Pairwise comparison of the NCBI SRA sheep dataset.


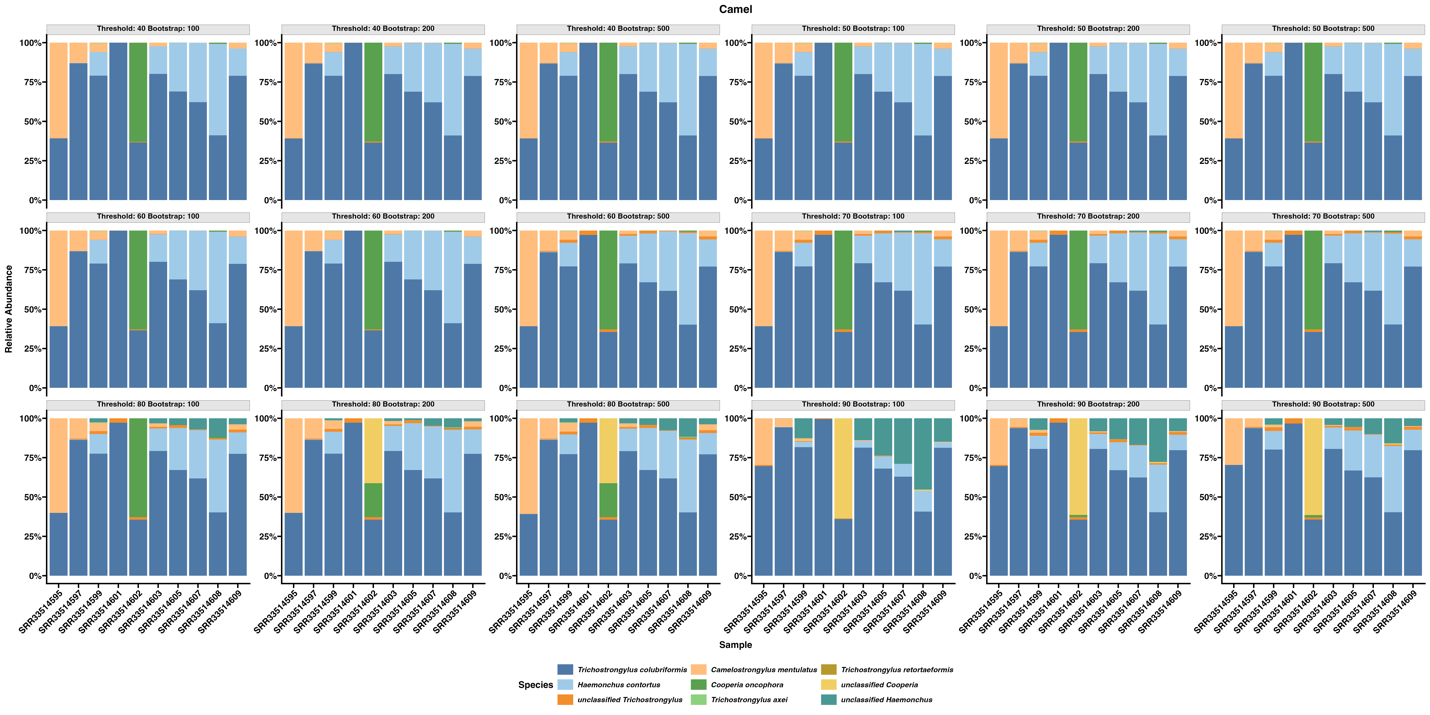


### Figure S14: Barplot showing the effect of threshold and bootstrap settings in R DADA2 on relative abundance estimation in camel nemabiome communities.


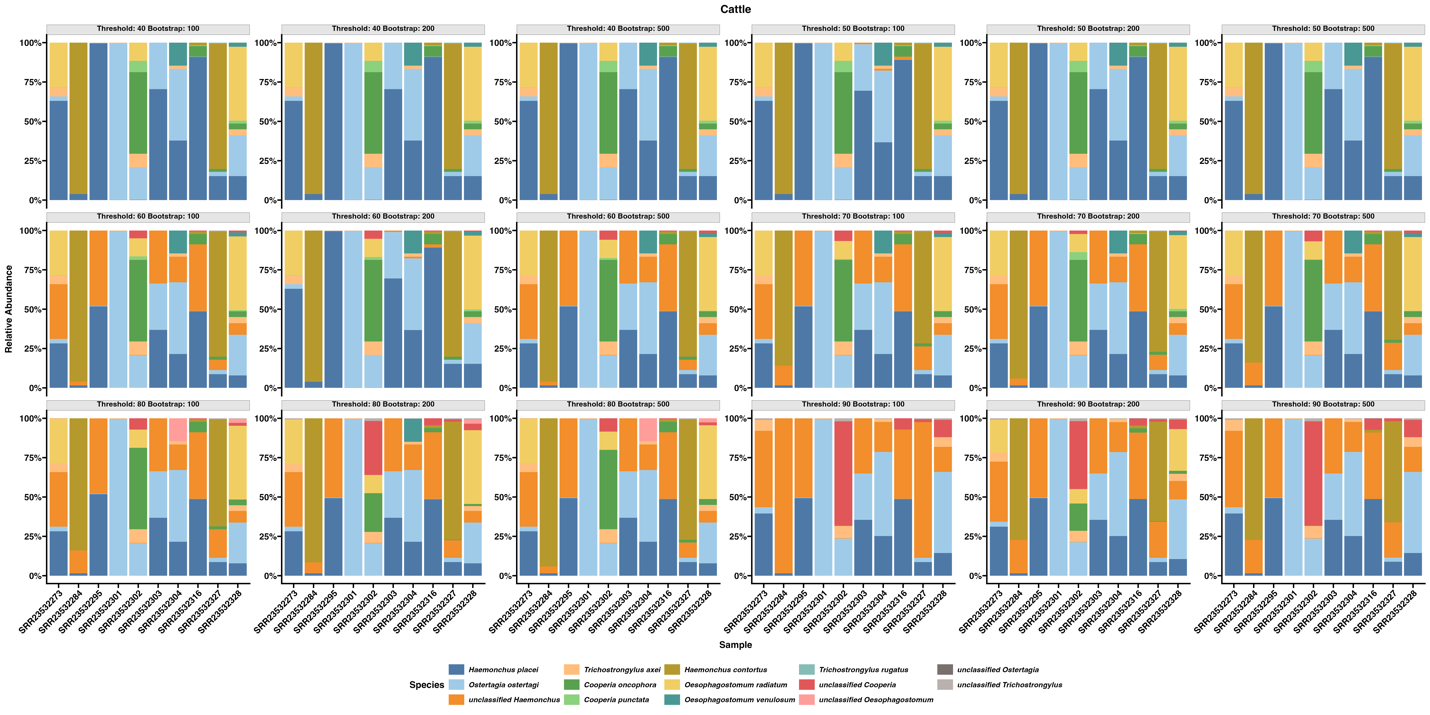


### Figure S15: Barplot showing the effect of threshold and bootstrap settings in R DADA2 on relative abundance estimation in cattle nemabiome communities.


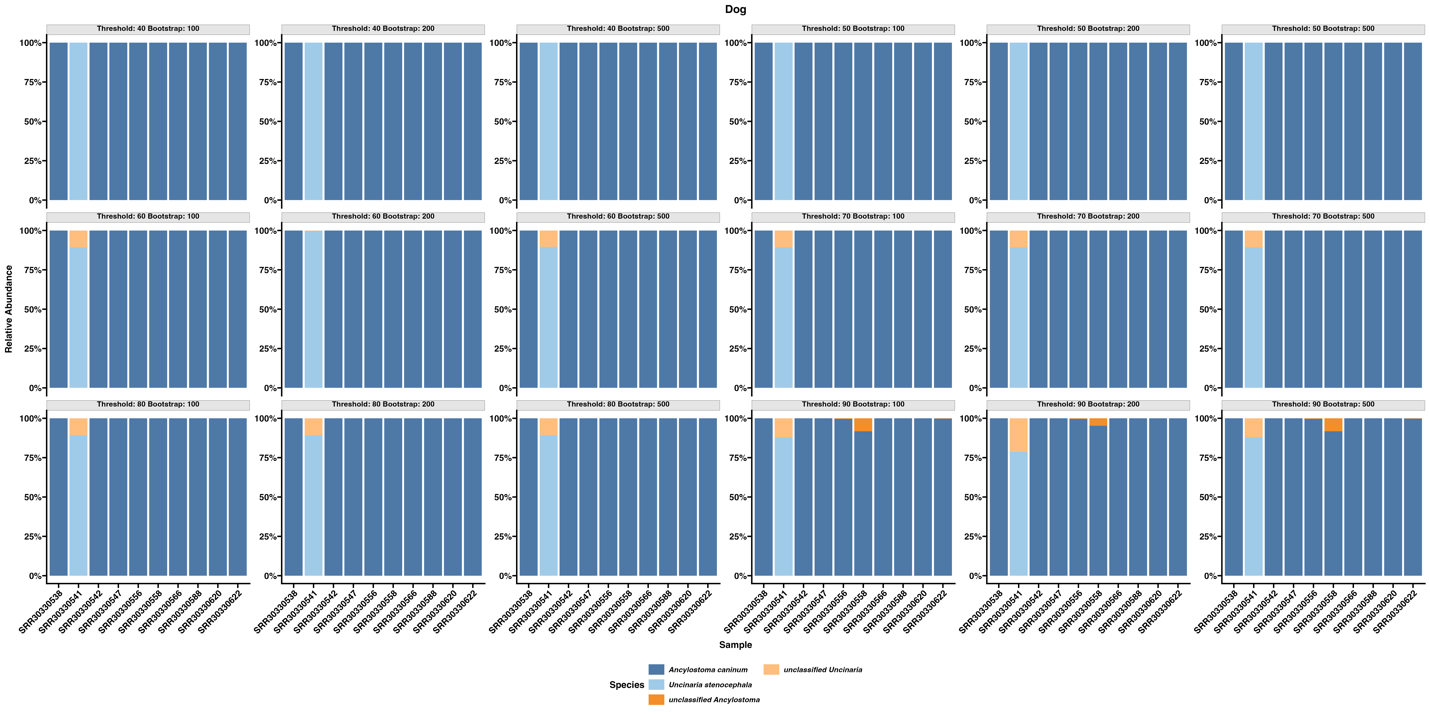


### Figure S16: Barplot showing the effect of threshold and bootstrap settings in R DADA2 on relative abundance estimation in dog nemabiome communities.


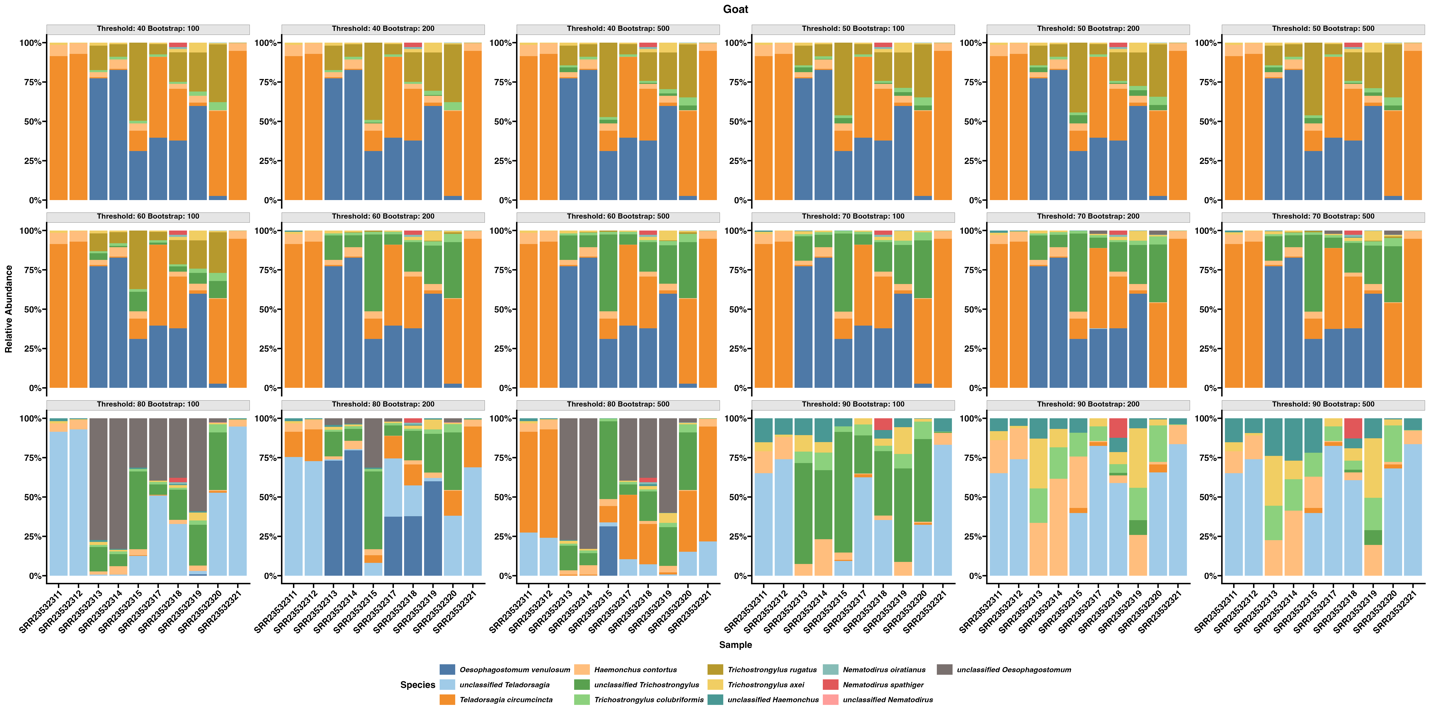


### Figure S17: Barplot showing the effect of threshold and bootstrap settings in R DADA2 on relative abundance estimation in goat nemabiome communities.


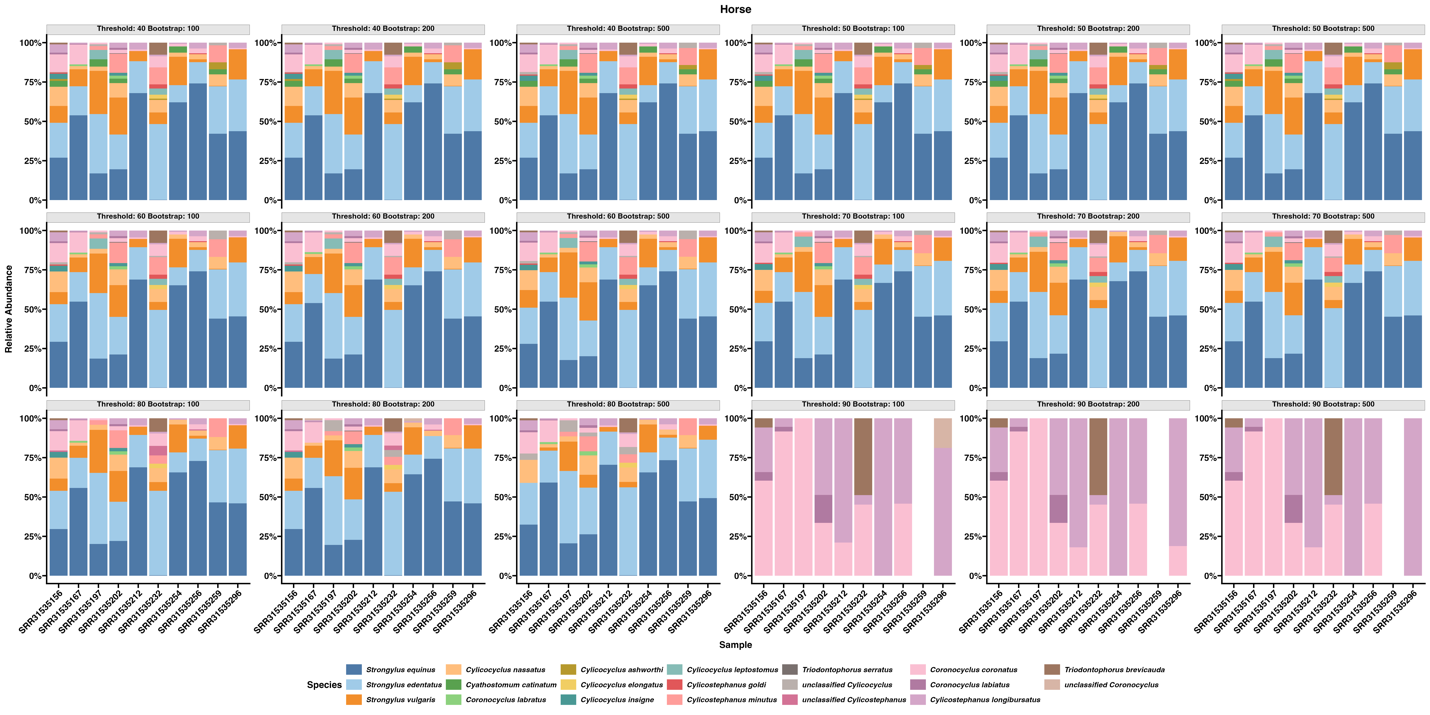


### Figure S18: Barplot showing the effect of threshold and bootstrap settings in R DADA2 on relative abundance estimation in horse nemabiome communities.


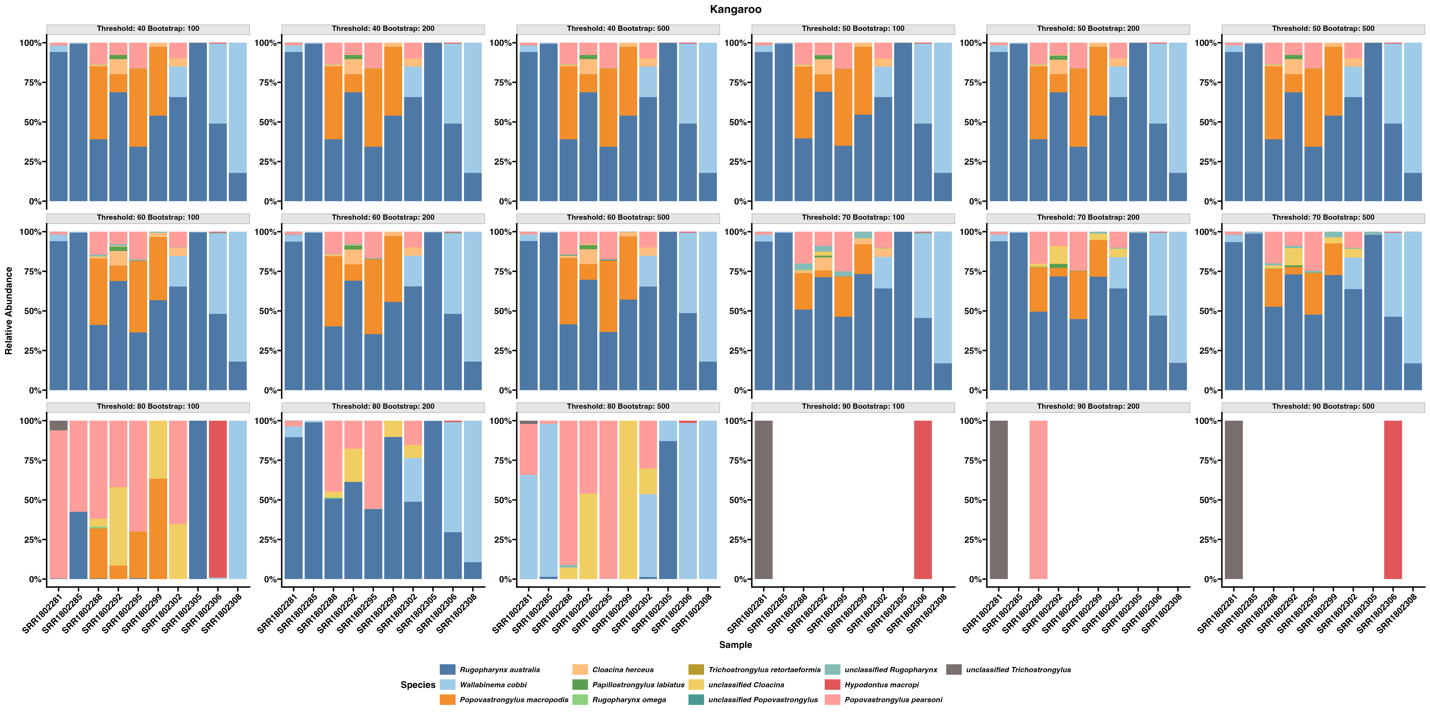


### Figure S19: Barplot showing the effect of threshold and bootstrap settings in R DADA2 on relative abundance estimation in kangaroo nemabiome communities.


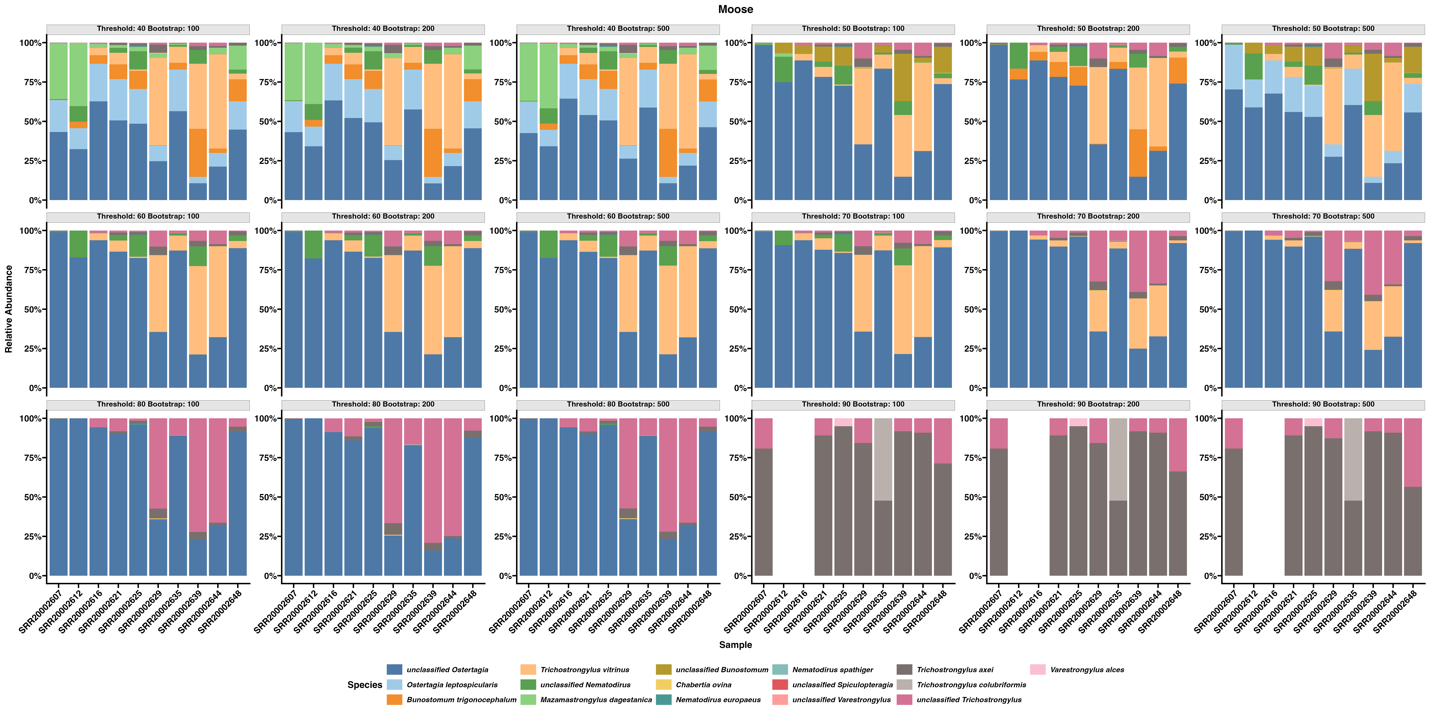


### Figure S20: Barplot showing the effect of threshold and bootstrap settings in R DADA2 on relative abundance estimation in moose nemabiome communities.


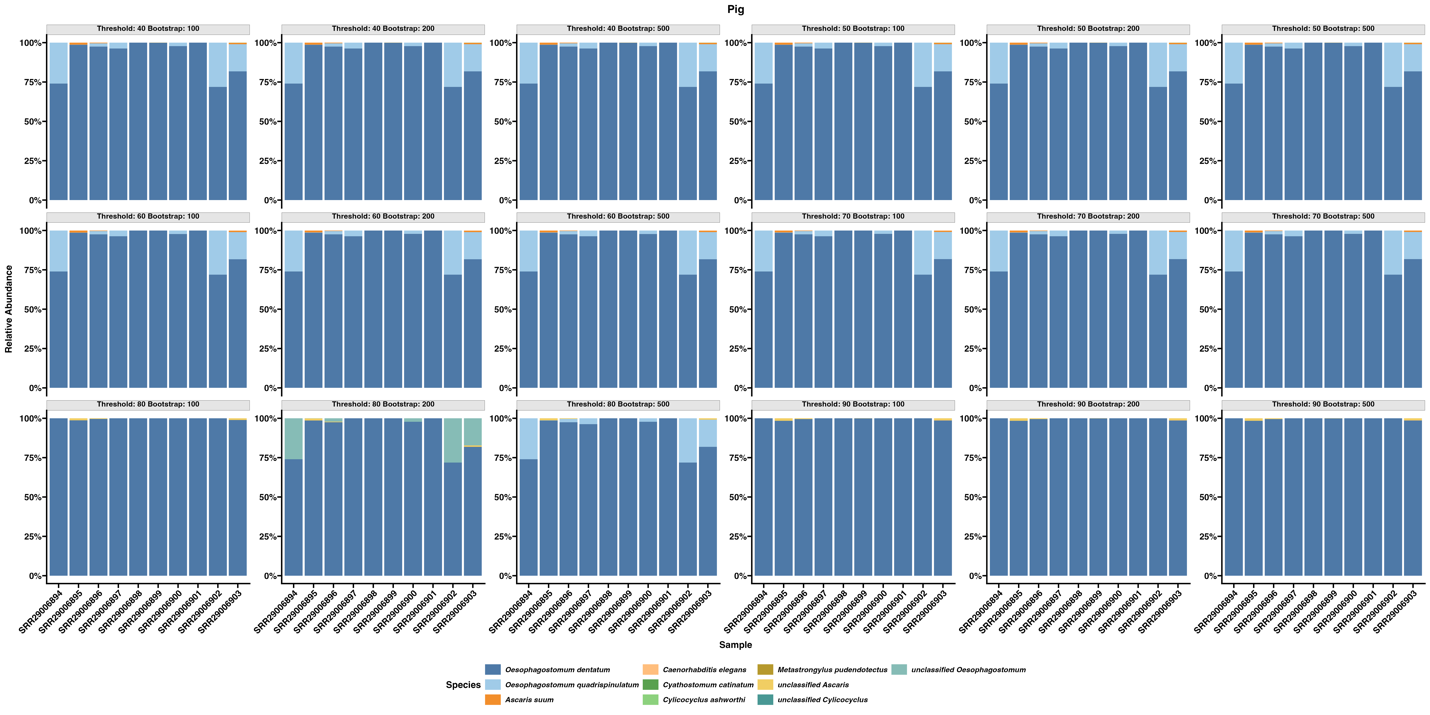


### Figure S21: Barplot showing the effect of threshold and bootstrap settings in R DADA2 on relative abundance estimation in pig nemabiome communities.


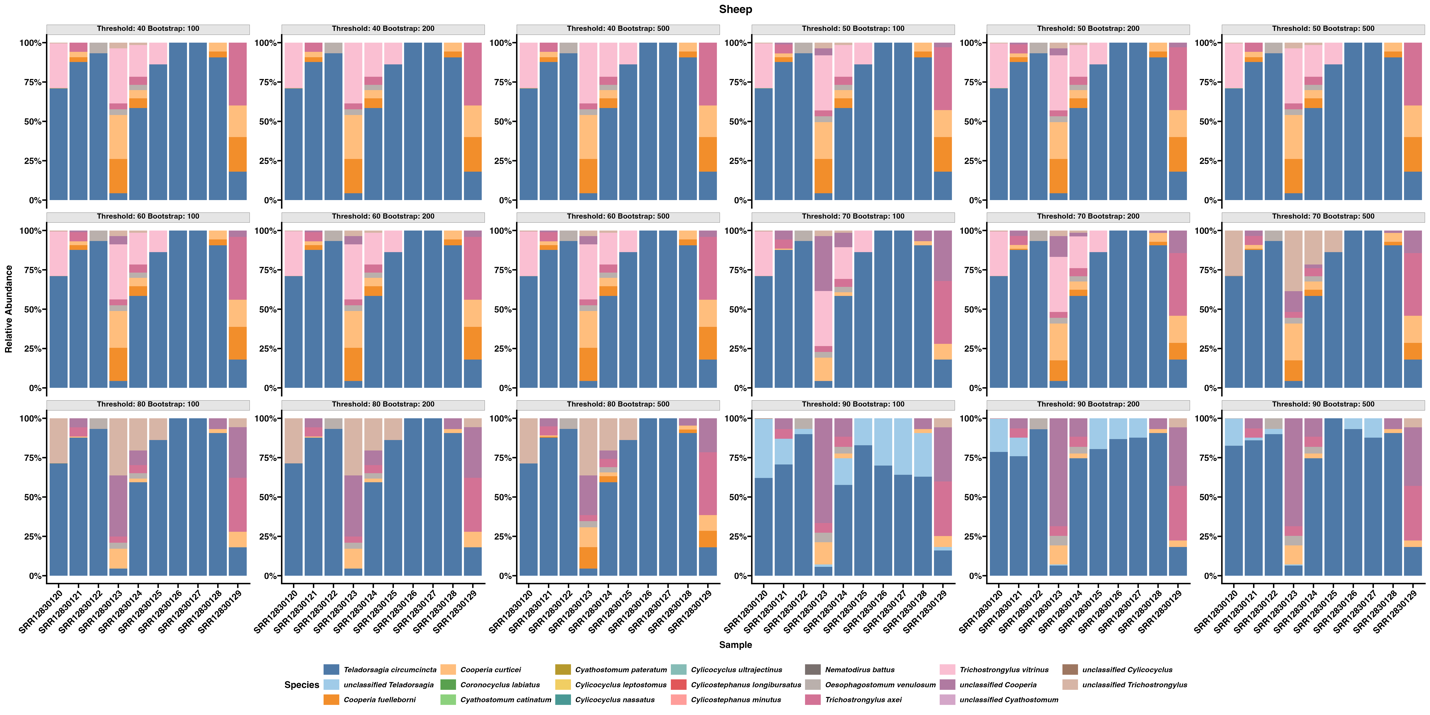


### Figure S22: Barplot showing the effect of threshold and bootstrap settings in R DADA2 on relative abundance estimation in sheep nemabiome communities.


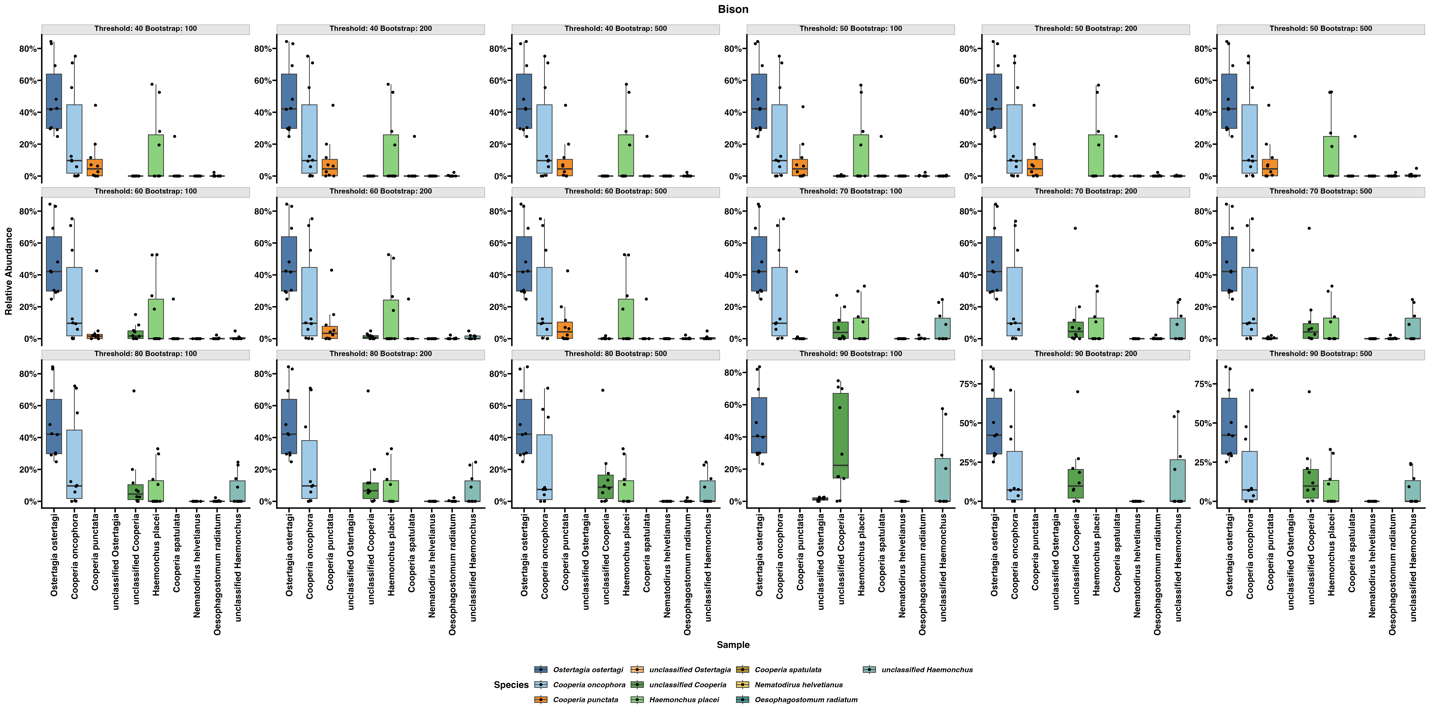


### Figure S23: Boxplot showing the effect of threshold and bootstrap settings in R DADA2 on relative abundance estimation in bison nemabiome communities.


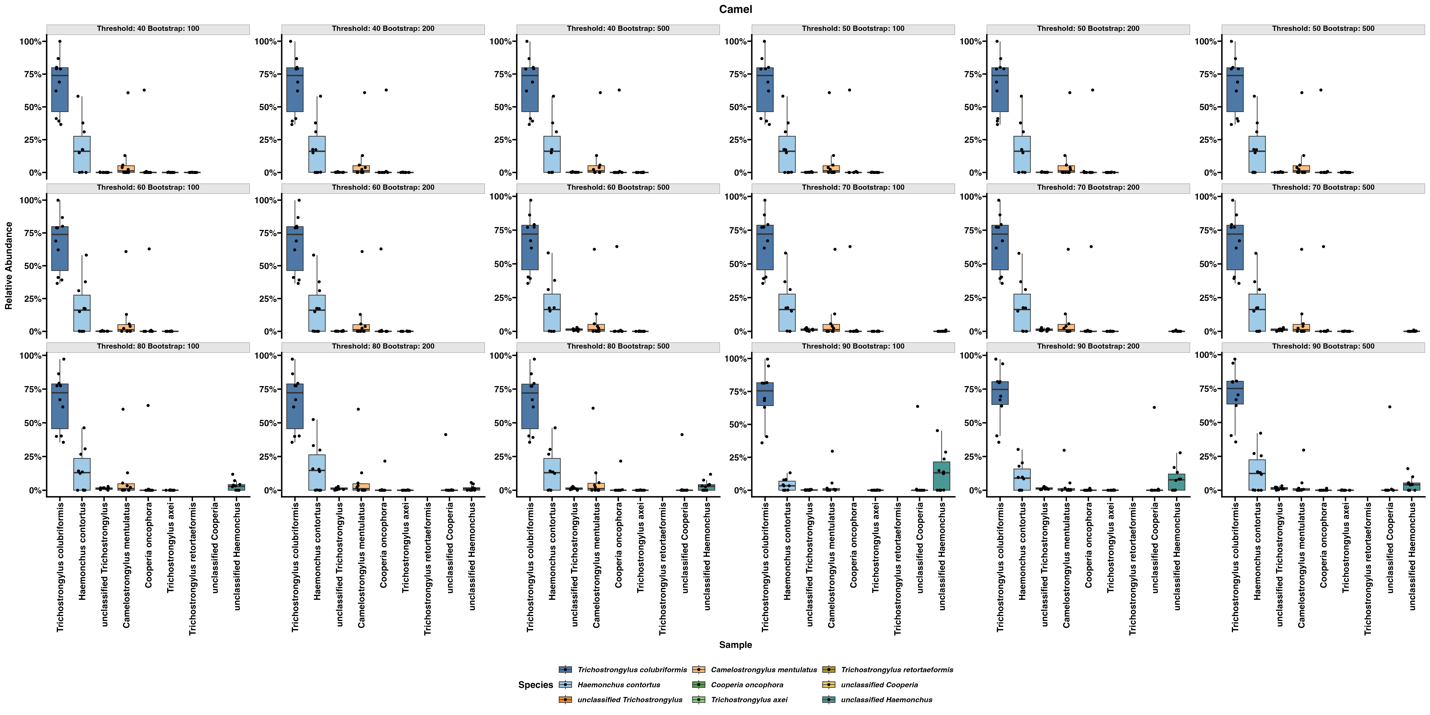


### Figure S24: Boxplot showing the effect of threshold and bootstrap settings in R DADA2 on relative abundance estimation in camel nemabiome communities.


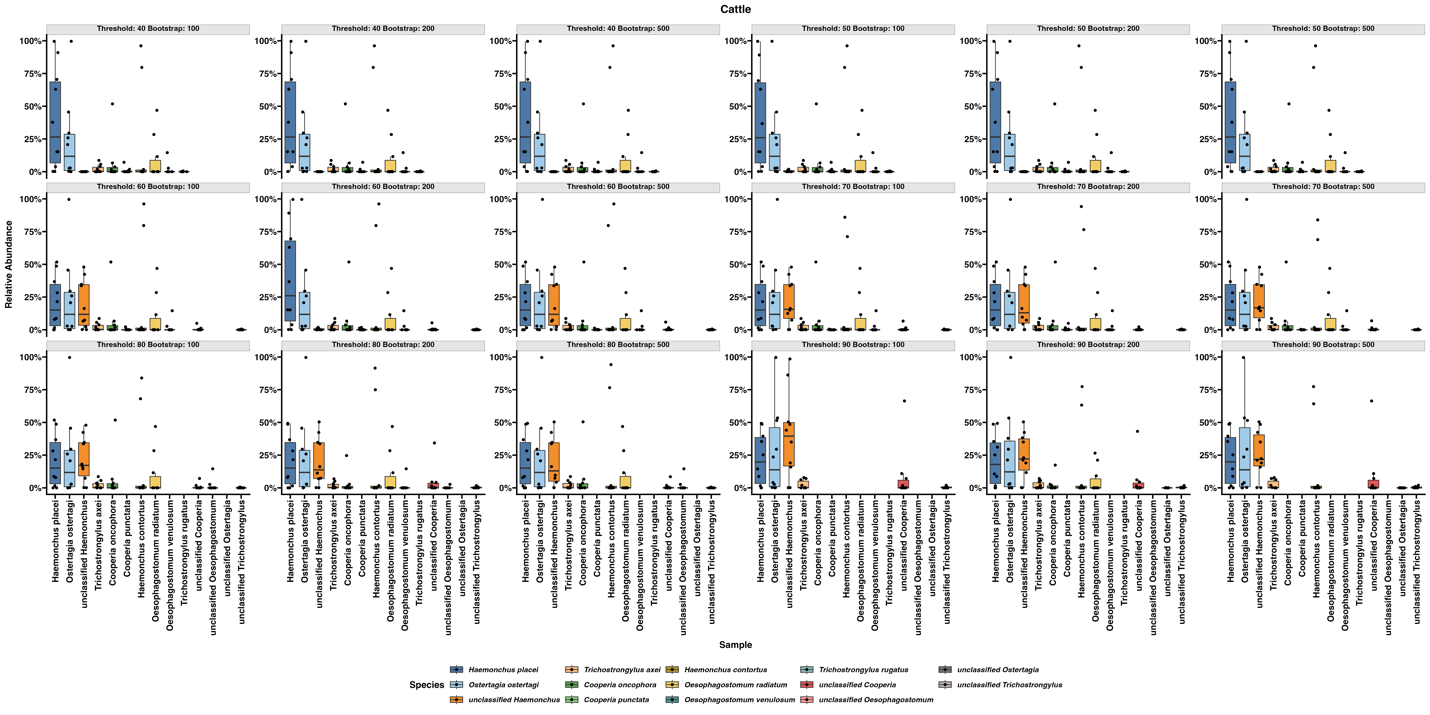


### Figure S25: Boxplot showing the effect of threshold and bootstrap settings in R DADA2 on relative abundance estimation in cattle nemabiome communities.


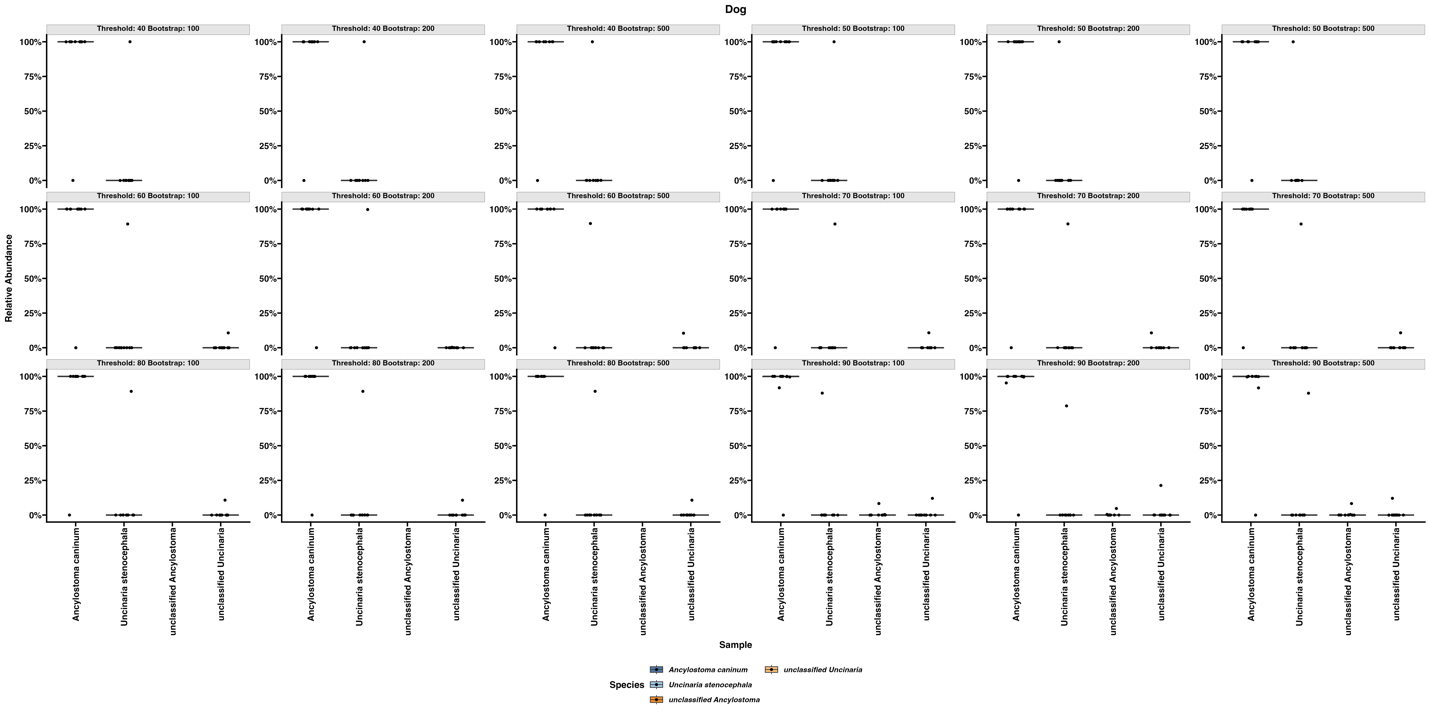


### Figure S26: Boxplot showing the effect of threshold and bootstrap settings in R DADA2 on relative abundance estimation in dog nemabiome communities.


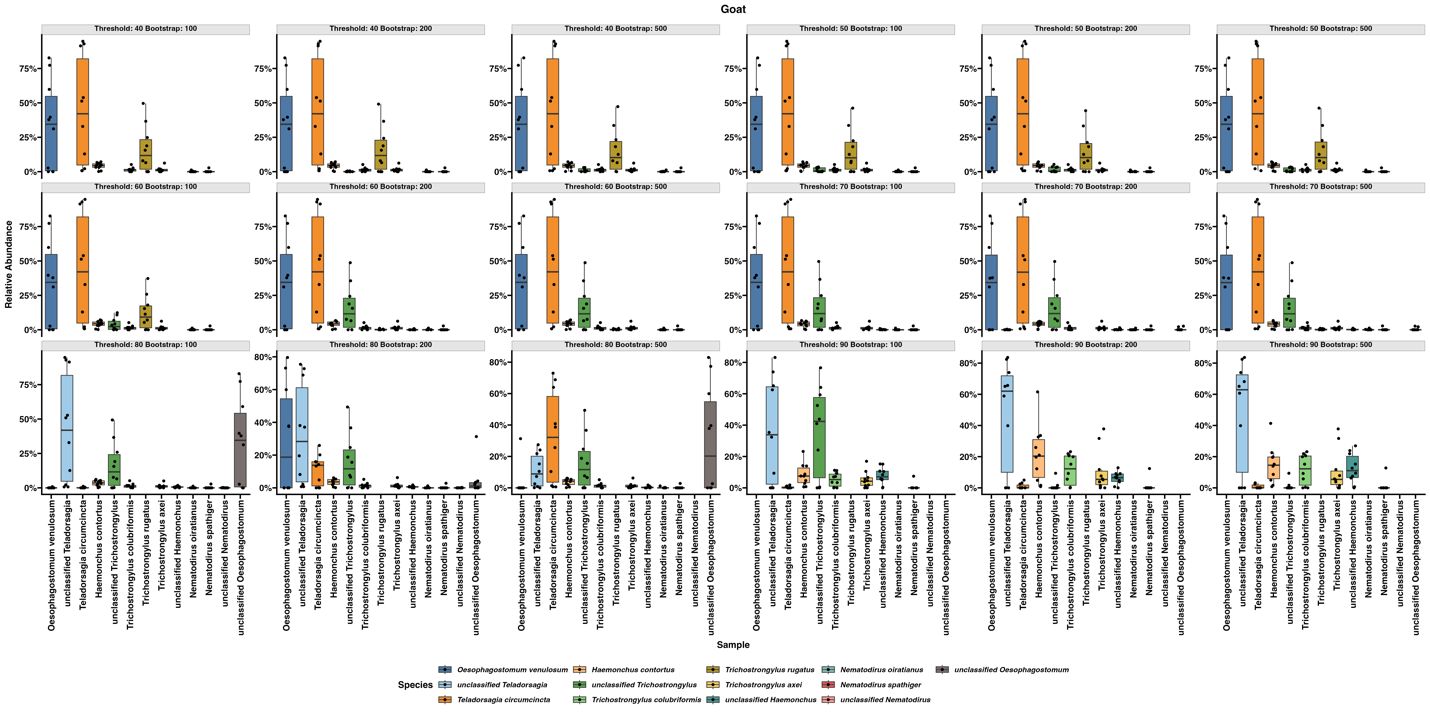


### Figure S27: Boxplot showing the effect of threshold and bootstrap settings in R DADA2 on relative abundance estimation in goat nemabiome communities.


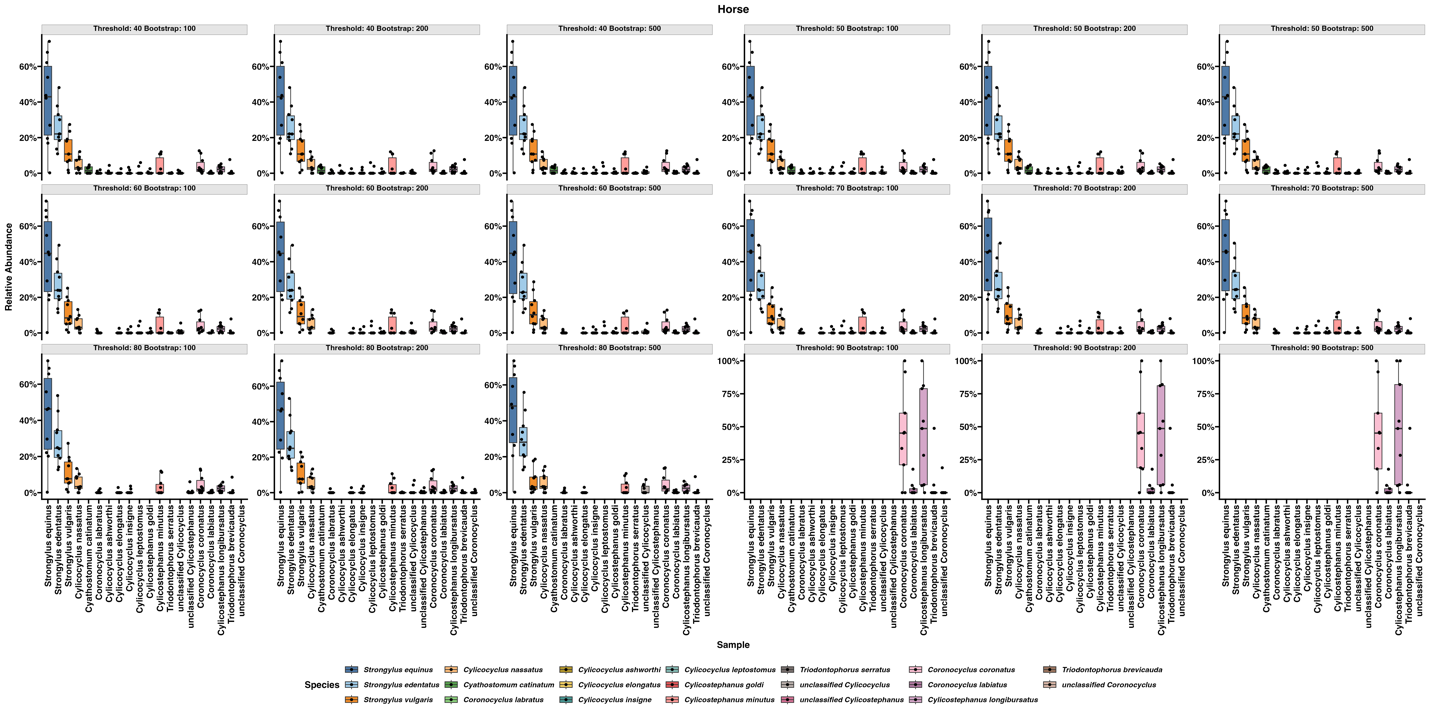


### Figure S28: Boxplot showing the effect of threshold and bootstrap settings in R DADA2 on relative abundance estimation in horse nemabiome communities.


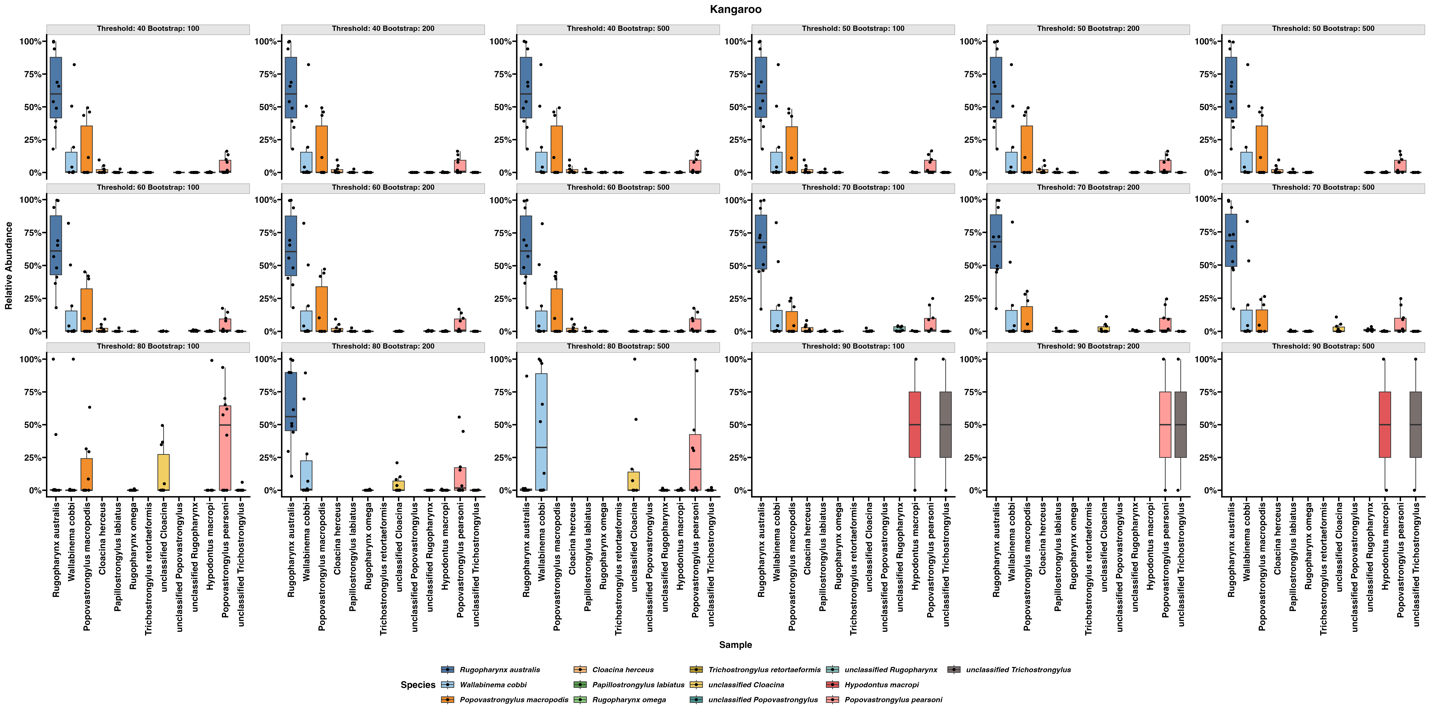


### Figure S29: Boxplot showing the effect of threshold and bootstrap settings in R DADA2 on relative abundance estimation in kangaroo nemabiome communities.


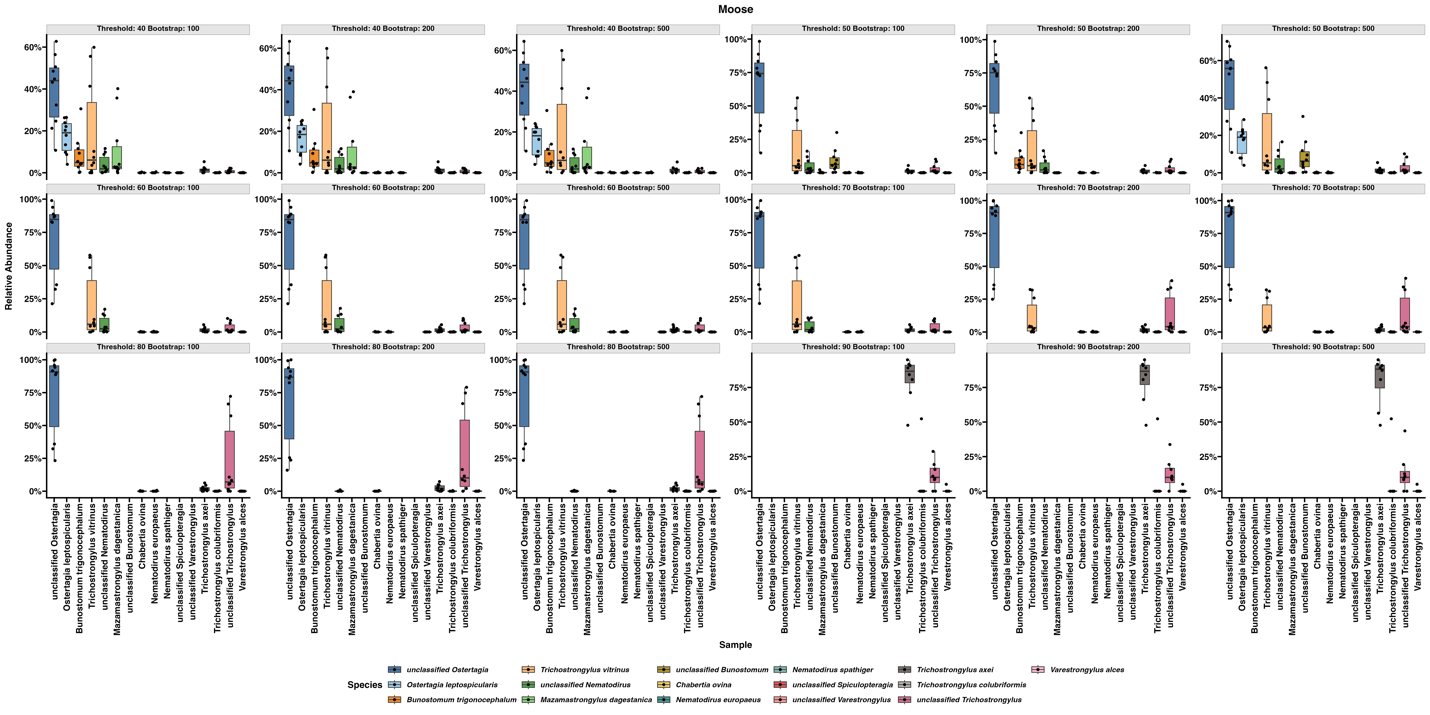


### Figure S30: Boxplot showing the effect of threshold and bootstrap settings in R DADA2 on relative abundance estimation in moose nemabiome communities.


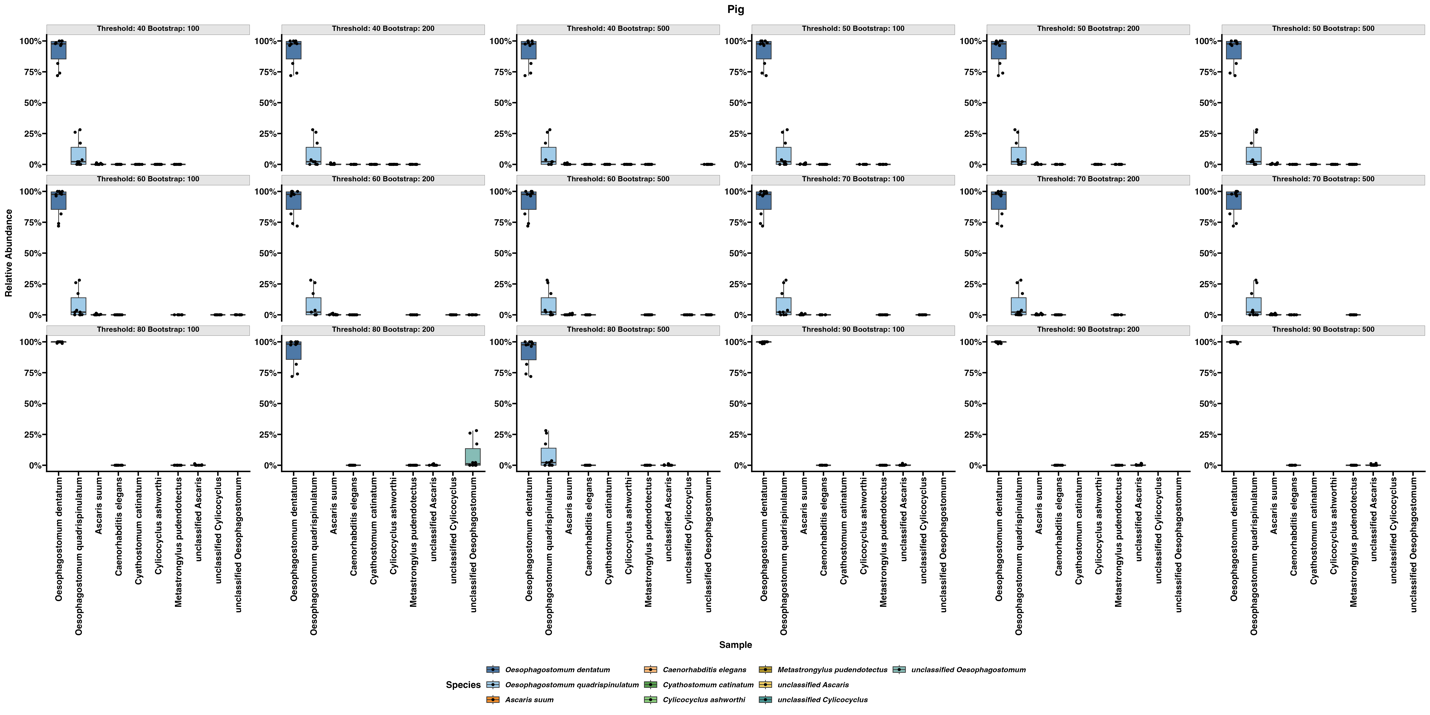


### Figure S31: Boxplot showing the effect of threshold and bootstrap settings in R DADA2 on relative abundance estimation in pig nemabiome communities.


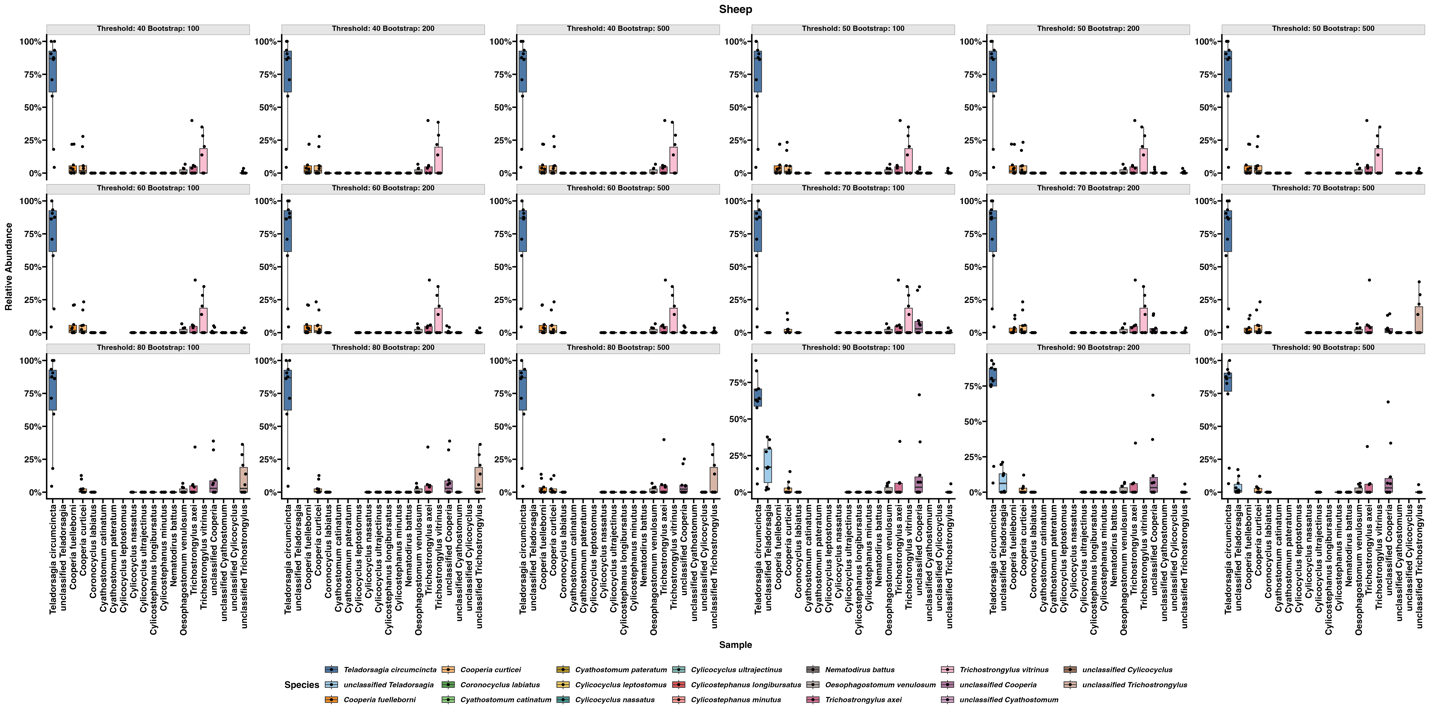


### Figure S32: Boxplot showing the effect of threshold and bootstrap settings in R DADA2 on relative abundance estimation in sheep nemabiome communities.
